# An unexplored coupling process enhances dark Hg(II) reduction in mineral-Hg(II)-DOM ternary systems

**DOI:** 10.1038/s41467-026-72424-6

**Published:** 2026-04-24

**Authors:** Ruiyang Sun, Guoming Lin, Yanping Li, Jinhang Wang, Ziyin Liu, Runsheng Yin, Ruoyu Sun, Lizhong Zhu, Jiating Zhao, Baohua Gu

**Affiliations:** 1 State Key Laboratory of Soil Pollution Control and Safety, Zhejiang University, Hangzhou, China; 2 Zhejiang Provincial Key Laboratory of Organic Pollution Process and Control, Hangzhou, China; 3 State Key Laboratory of Green and Efficient Development of Phosphorus Resources & School of Future Technology, Fuzhou, China; 4 State Key Laboratory of Critical Mineral Research and Exploration, Institute of Geochemistry, Chinese Academy of Sciences, Guiyang, China; 5 Institute of Surface-Earth System Science, School of Earth System Science, Tianjin University, Tianjin, China; 6 ZJU-Hangzhou Global Scientific and Technological Innovation Center, Hangzhou, China; 7 Environmental Sciences Division, Oak Ridge National Laboratory, Oak Ridge, TN USA

**Keywords:** Geochemistry, Environmental impact

## Abstract

Photochemical and microbial-mediated reduction of mercury (Hg) are recognized as key processes and pathways for Hg geochemical cycling and atmospheric emissions. However, these processes cannot fully account for the total budget of global elemental Hg(0) production. Here we show that net Hg(0) production is significantly enhanced in a goethite-Hg(II)-DOM ternary system under dark conditions, representing a ubiquitous mineral-organic interface in surface ecosystems. This enhancement arises from the combined effects of preferential adsorption of monodentate [Hg-DOM]^+^ complexes, retention of electron-donating DOM in the aqueous phase, and lower interfacial energy barriers for Hg(II) reduction by goethite. We estimate that ternary interactions, as an underappreciated source of terrestrial Hg(0) emissions, release about 6.3% of topsoil Hg to the atmosphere. These findings underscore the need to integrate mineral-Hg(II)-DOM ternary systems into predictive models of the global Hg cycle and associated ecological risks.

## Abstract (web summary)

Sun et al. show that a synergistic coupling in soil mineral-Hg(II)-organic matter ternary systems markedly promotes Hg(II) dark reduction, thereby liberating ~6.3% of terrestrial surface Hg and potentially impacting global Hg cycling.

## Introduction

Mercury (Hg) is a toxic pollutant of global concern due to its persistence, long-range transport, and its tendency to accumulate and biomagnify in food webs. In response to these risks, global Hg emissions and releases are governed by the Minamata Convention, which aims to curb anthropogenic Hg inputs and mitigate the resulting ecological and human health impacts. In natural environments, Hg undergoes dynamic redox transformations among divalent mercury (Hg(II)), gaseous elemental Hg (Hg(0)), and organo-metallic methylmercury (MeHg)^1,2^. Its chemical speciation and species distribution, shaped by complex reactions with environmental matrices, determine its mobility, transformation, bioavailability, and bioaccumulation across terrestrial and aquatic ecosystems^3,4^. Soils represent the largest terrestrial Hg sink, retaining about 60–90% of global land-based Hg^5^. The fate and reactivity of Hg are further governed by its interactions with soil organic matter, sulfides, and minerals in natural environments^6^. Over 95% of Hg(II) is usually bound to mineral surfaces or organic matter (OM)-coated minerals via adsorption and complexation^4^. However, the molecular mechanisms and environmental significance underlying these interactions on the fate of Hg remain poorly understood and have limited our ability to apprehend and predict global Hg cycling, behavior, and its associated risks.

Dissolved organic matter (DOM) and iron (Fe) (oxyhydr)oxides represent key reactive components with Hg in terrestrial and aquatic environments, exerting profound controls over Hg biogeochemical cycling and its fate^7,8^. Upon entering the aqueous phase (e.g., pore water, overland runoff, groundwater, river), Hg(II) is expected to be preferentially complexed by free DOM due to the rapid and thermodynamically strong association between dissolved ligands and Hg^9,10^, while DOM immobilized on mineral surfaces may exhibit reduced accessibility for metal binding^11,12^. As a result, DOM-Hg(II) complexes likely represent an important precursor pool before subsequent interactions with mineral phases. Although strong mineral-DOM interactions are well documented, how minerals influence the interfacial structure, mobility, and transformation of DOM-Hg(II) complexes remains poorly constrained. In the absence of Hg, the adsorption of DOM onto Fe (oxyhydr)oxides induces molecular fractionation, preferentially retaining aromatic and carboxylic-rich DOM constituents on mineral surfaces^13,14^. This selective partitioning alters DOM composition and stability^15^, while potentially modulating its photochemical reactivity^16^ and electron transfer capacity^17^. We hypothesize that DOM fractionation can modify Hg coordination conditions and thereby influence subsequent interactions between Hg-DOM complexes and mineral surfaces in “mineral-Hg(II)-DOM” ternary system interactions^1,18,19^. Furthermore, Hg binding affinities with both DOM and minerals may concurrently influence DOM fractionation patterns and mineral reactivity. These intricate, interdependent reaction processes result in diverse Hg coordination modes in ternary surface complexes, which may include a) ionic bridging structures linking DOM and minerals and b) direct adsorption of Hg-DOM complexes onto mineral surfaces or being incorporated into mineral structures^20,21^.

The modes and mechanisms concerning ternary mineral-Hg(II)-DOM interactions in turn affect the environmental fate of Hg and DOM, but these processes and interactions so far have been largely overlooked. Major discrepancies remain regarding their influences on Hg fate and cycles. For instance, while fulvic acid (FA) has been shown to promote Hg(II) adsorption onto goethite due to selective binding of FA with Hg (via thiol complexation) and its sorption on goethite^22^, Liang et al^20^. reported that humic acid (HA) suppressed Hg(II) adsorption onto Fe/Mn oxyhydroxides in freshwater systems due to competitive reactions with limited surface adsorption sites. Other studies, however, found that HA could enhance Hg(II) adsorption on Fe and Mn oxides by forming stable organic halides with Cl^-^, thereby reducing the concentration of non-sorbing HgCl_2_ species^23^. These apparent contradictions underscore an unresolved yet important role of mineral-Hg(II)-DOM ternary interactions in controlling Hg mobilization and transformation processes with direct consequences on Hg fluxes into the atmosphere, hydrosphere, and food webs^21^. Such coupled mineral-Hg(II)-DOM interactions may be especially relevant in dark sediments and subsurface environments, where minerals and DOM are abundant but photochemical Hg reduction is limited. While Hg redox reactions in pure mineral or DOM systems have been studied extensively, the coupled electron-transfer mechanisms within mineral-DOM composites, particularly those involving multiscale proton-electron coupling, remain elusive. Deciphering these interfacial redox reactions and pathways is thus vital for advancing predictive understanding and models of Hg cycling in environmental systems.

Here, we reveal the physicochemical mechanisms governing Hg(II) partitioning and dark reduction in mineral-Hg(II)-DOM ternary systems across environmentally representative Hg/DOC ratios and spanning from pristine to anthropogenically impacted soil-water environments. In porewater, groundwater, and wetland systems, a substantial fraction of DOM remains mobile, and Hg(II) predominantly occurs as DOM-associated species even at low Hg/DOM ratios. This leads to an initial homogeneous complexation between Hg(II) and DOM in solution, followed by heterogeneous interactions of DOM-Hg species with Fe (oxyhydr)oxide surfaces. On this basis, we focus on this typical scenario to investigate how the transition from solution-phase complexation to mineral-water interfacial reactions regulates the transformation of Hg(II). By integrating batch experiments with Fourier transform ion cyclotron resonance mass spectrometry (FT-ICR-MS) and Synchrotron radiation X-ray absorption spectrometry (SR-XAS), we elucidate molecular-level DOM fractionation patterns and their roles on Hg conversion in the ternary systems. Density functional theory (DFT) calculations further unveil Hg(II) adsorption configurations and reduction pathways in the presence or absence of mineral (goethite). This work provides direct mechanistic evidence by explicitly disentangling the coupled roles of solution-phase DOM reactivity and mineral-associated interfacial configurations in controlling dark Hg(II) reduction. Moreover, the combined experimental-theoretical approach presented offers a generally applicable strategy for linking molecular-scale mineral-Hg(II)-DOM interactions to Hg redox behaviors in the environment, and the results are expected to bridge the knowledge gap in understanding Hg cycling dynamics in the terrestrial and aqueous ecosystems.

## Results and discussion

### Mercury partitioning in the Goethite-Hg(II)-DOM Ternary System

**Fig. 1:**
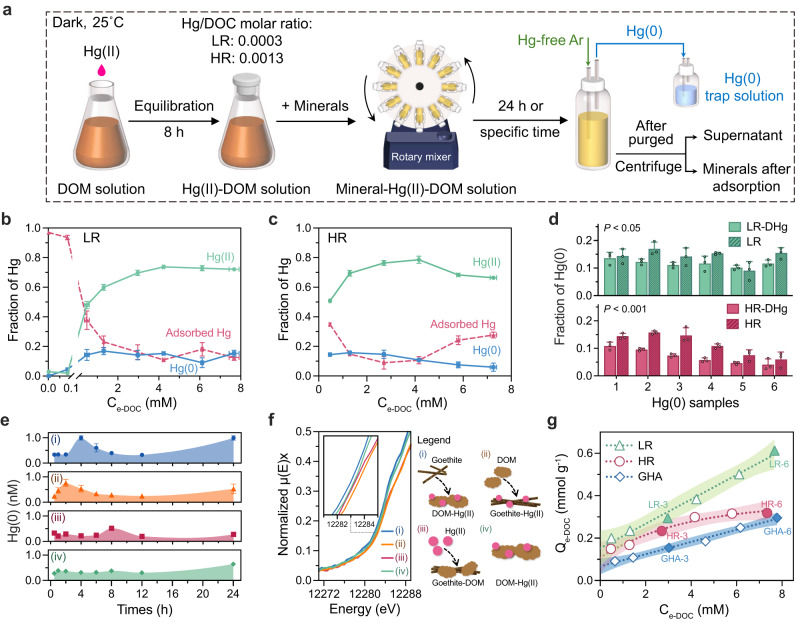
Hg partitioning and reduction in the goethite-Hg(II)-DOM system. **a** Schematic for the sorption experiments. All experiments were carried out at 25°C under dark conditions. **b**, **c** Fractions of Hg(II) in isothermal adsorption experiments of Hg and DOM on goethite at a low Hg/DOC ratio (LR, *n* = 3, **b**) and a high ratio (HR, *n* = 3, **c**). **d** Hg(0) production in LR and HR (with goethite) and in the corresponding control systems without goethite (LR-DHg and HR-DHg, *n* = 3). Numbers 1–6 refer to the six isothermal adsorption experiments and follow the same left-to-right order as the data points in **b** and **c**. Statistical significance was assessed using paired *t*-tests comparing experiments with and without goethite under otherwise identical conditions. **e** Changes in Hg(0) production over time in four sequential kinetic experiments (Hg(II): 7 nM, DOC: 0.83 mM, goethite: 2 g L^-1^). The order of reactant addition is illustrated in the legend in **f**. **f** Hg L_III_ XAS spectra (12270–12290) of four sequential kinetic experiments. The inset shows an enlarged view of the 12281–12284 eV region. **g** Adsorption isotherms of DOM on goethite in LR, HR, and goethite with DOM (GHA) systems. GHA (without Hg(II)) serves as a control experiment of DOM and goethite (*n* = 3). C_e_ represents equilibrium concentrations in solution; Q_e_ represents the adsorbed amount of DOM on goethite. The shaded areas denote the 95% confidence intervals. Color-filled points in **g** are samples selected for FT-ICR-MS analyses, “-3” and “-6” refer to the third and sixth samples obtained from the isothermal adsorption experiments. All graphs are represented as means ± s.d. Source data are provided in the Source Data file.

Isothermal adsorption experiments were conducted to investigate interactions among goethite, DOM (terrestrial humic acid (HA) as a proxy), and Hg(II) under two environmentally relevant Hg/DOC molar ratios: a lower ratio (LR, 0.0003) and a higher ratio (HR, 0.0013) (Fig. 1a). The initial DOC concentration was maintained constant across all experiments to ensure comparability. Goethite was selected as the primary Fe oxyhydroxide in this study owing to its broad occurrence and relative persistence in soils and sediments^24^. Its structural stability constrains the influence of phase transformation and permits clearer resolution of interfacial processes. Extensive use of goethite in studies of DOM molecular fractionation^14,25^ also makes it an appropriate model for examining Hg coordination and reduction in mineral-associated DOM systems and for comparison with other Fe minerals. Control experiments included three binary systems: (1) goethite with DOM (GHA), (2) DOM with Hg(II) (two Hg/DOC ratios: LR-DHg and HR-DHg), and (3) Hg(II) with goethite (GHg). Notably, the collectable Hg(0) reflects the balance of reduction of Hg(II) and re-oxidation of Hg(0).

In the absence of goethite, the Hg(0) yield in the LR-DHg system (LR without goethite) remained relatively stable (13.5% to 10.2%), whereas the HR-DHg system (HR without goethite) exhibited a notable decline in Hg(0) (10.8% to 4.1%) as the Hg(II) and DOC concentrations increased (Fig. 1b, c). Hg(0) production at the LR ratio aligned well with the optimal range (0.24–0.8 × 10^⁻3^) previously identified for DOM-mediated Hg(II) reduction^26^. At a high HR ratio, the reduction of Hg(II) is constrained by both the reducing capacity of DOM available per Hg(II) molecule and the speciation of Hg(II), while redox-active DOM functional groups also contributed to the reoxidation of Hg(0). Meanwhile, more Hg(II) was adsorbed in HR (C_e-DOC_ > 4.17 mM, Fig. 1c) than in LR (Fig. 1b) at increasing DOM concentrations, resulting in further decreased reducible Hg(II) and net Hg(0) yields. Paired *t*-test analysis confirmed that the presence of goethite significantly enhanced Hg(0) production in both LR and HR systems (*p* < 0.05 and *p* < 0.001, respectively) (Fig. 1d), as negligible amounts of Hg(II) reduction were observed in the GHg only system (Supplementary Fig. 3). These findings suggest that goethite indirectly facilitated Hg(0) generation, likely by altering DOM molecular compositions or enabling surface-mediated electron transfers to Hg(II).

We further explored the roles of hematite and magnetite, two other important Fe minerals in natural environments, on Hg(II) dark reduction, comparing their effects to those of goethite in the mineral-Hg(II)-DOM ternary system. The results showed that hematite significantly inhibited Hg(0) production in the hematite-Hg(II)-DOM system, while Hg(0) production in the magnetite-Hg(II)-DOM system was comparable to that in the DOM-Hg(II) binary system (Supplementary Fig. 4a). The supernatant electron-donating capacity (EDC) experiments demonstrate that Hg(II) alters the partitioning and redox availability of DOM at mineral surfaces, which in turn affects Hg speciation and transformation in a mineral-dependent manner. In the hematite-Hg(II)-DOM ternary system, no EDC was detected at pH 5.5 (Supplementary Fig. 4b), which explains why almost no Hg(0) was produced in the hematite-Hg(II)-DOM system, as any Hg(0) generated is readily re-oxidized by the highly electron-accepting solution. The depletion of electron-donating moieties is consistent with previous reports that hematite strongly and almost irreversibly adsorbs carboxyl, phenolic, and other redox-active functional groups at pH 5.5^27^. The presence of Hg(II) appears to amplify this affinity by promoting additional DOM accumulation at the hematite surface (Supplementary Fig. 4b, c), thereby reinforcing electron depletion in the aqueous phase and suppressing sustained Hg(II) reduction. Magnetite shows a weaker affinity for DOM than goethite and hematite, resulting in greater DOM retention in solution and higher supernatant EDC values (Supplementary Fig. 4). In the magnetite-DOM binary system, measurable Fe(II) release was observed (21.96, 15.71, and 17.68 µM at 2, 12, and 24 h, Supplementary Fig. 4e), whereas Fe(II) concentrations were lower in the magnetite-Hg(II)-DOM ternary system (23.75, 13.04, and 10.36 µM, Supplementary Fig. 4f). Similar differences were also observed in the systems of goethite and hematite (Supplementary Fig. 4d, e), with lower Fe(II) concentrations in the ternary systems than in the corresponding mineral-DOM binary systems. However, net Hg(0) production showed a closer relationship with EDC than with aqueous Fe(II), suggesting that Fe(II) release did not account for the mineral-dependent differences in net Hg(0) production. DOM fractionation and consequent changes in redox-active DOM components, as well as interfacial Hg speciation in the mineral-Hg(II)-DOM ternary system, may be the main drivers enhancing net Hg(0) production. As outlined at the beginning of this section, goethite was used as the primary mineral for the mechanistic investigation, whereas hematite and magnetite were included as comparative systems to help interpret mineral-dependent differences in Hg redox behavior.

### Mechanistic insights into goethite-DOM-mediated Hg redox dynamics

To examine the role of goethite in Hg(0) production under environmentally realistic conditions, sequential addition experiments were conducted using 7 nM Hg(II), a concentration representative of background Hg levels in natural soils and porewaters (Fig. 1e and Supplementary Fig. 5). The experiment was performed first in a binary system, and a third reactant was introduced 2 h after their initial reactions as follows: (i) DOM with Hg(II), then goethite, (ii) goethite with Hg(II), then DOM, (iii) goethite with DOM, then Hg(II), and (iv) DOM with Hg(II) (control). We found that Hg(0) levels remained stable (~0.34 nM, average Hg(0) production within 12 h) in the control experiment (iv), similar to that observed in a previous study at comparable Hg concentrations^28^. The introduction of goethite in experiment (i) significantly enhanced Hg(0) production (0.91 nM vs. 0.32 nM at 4 h, in experiment (i) and control experiment (iv)), though yields gradually decreased thereafter. This may reflect the changing balance among DOM-mediated Hg(II) reduction, Hg(0) reoxidation^19^, time-dependent rearrangement of Hg(II) from O/N- to S- containing ligands^26^, and partitioning of Hg(0) between the aqueous phase and the headspace in the closed vial^29^. Hg(II) initially binds preferentially to O/N-rich functional groups, which promotes rapid reduction but also facilitates Hg(0) re-oxidation^19,24^. Measurements of DOC and EDC in the supernatant (Supplementary Fig. 6a) further showed that part of the DOM was released back into solution between 12 and 24 h. This likely resulted from desorption of weakly bound DOM from the mineral surface, a behavior commonly observed for non-carboxyl-rich DOM fractions^11^. Such redistribution may preserve a reactive pool of electron-donating components in solution and sustain further Hg(II) reduction. Together, these coupled processes are consistent with the higher net Hg(0) production in 24 h and support an overall promotive effect of goethite on Hg(0) production. Experiment (ii) showed net Hg(0) generation within the first 2 h (up to 0.76 nM) following DOM addition. In contrast, experiment (iii) exhibited suppressed Hg(0) production compared to the control (iv), suggesting that Hg(II) adsorption onto DOM-coated goethite limited its reducibility, whereas systems in which Hg(II) initially interacted with DOM retained a higher net Hg(0) production rate. X-ray absorption spectroscopy at the Hg L_III_ edge revealed strong correlation between the reaction order of goethite, DOM and Hg and Hg(0) production patterns (Fig. 1f). Specifically, experiment (i) displayed a characteristic energy shift toward the lower energy side compared to the control (iv), indicating the formation of more reduced Hg species, which agrees with the enhanced net Hg(0) production observed in Fig. 1e. Experiment (ii) and (iii) showed higher absorption edge energies than the control, indicating more oxidized Hg species leading to a lower Hg(0) yield. These results demonstrate that the formation of goethite-Hg(II)-DOM ternary complexes played a pivotal role in facilitating Hg(0) production.

In the GHg system, the adsorbed Hg(II) on goethite gradually decreased to about 13% but remained detectable across the tested concentration range (0.32–13.34 µM), indicating a non-saturating adsorption status (Supplementary Fig. 3). Similarly, DOM adsorbed Hg(II) in the GHA system increased continuously across the tested DOC range (0.83–8.75 mM). This suggests that adsorption sites on goethite were not saturated by DOM either (Fig. 1g). Both LR and HR systems showed declining Hg(II) adsorption on goethite (approximately 10%) at the equilibrium DOC concentration of 4.17 mM (Fig. 1b, c). Notably, the HR system exhibited increased Hg adsorption at higher DOC concentrations (4.17–8.33 mM), which was not observed in the LR system. Our study thus suggests that, while stable bidentate complexes (e.g., Hg(SR)_2_ and RS-Hg-O/NR) may dominate at low Hg(II) concentrations^30–32^, Hg likely existed mainly in the form of goethite-Hg(II)-DOM ternary complexes in LR and HR systems, and Hg(II) functioned as an ionic bridge linking goethite and DOM. These results indicate that the association of Hg-DOM complexes with mineral surfaces forms mineral-Hg(II)-DOM ternary complexes that are critical for Hg speciation and its dark reduction.

### Molecular fractionation of DOM on goethite affects Hg(II) redox dynamics

**Fig. 2:**
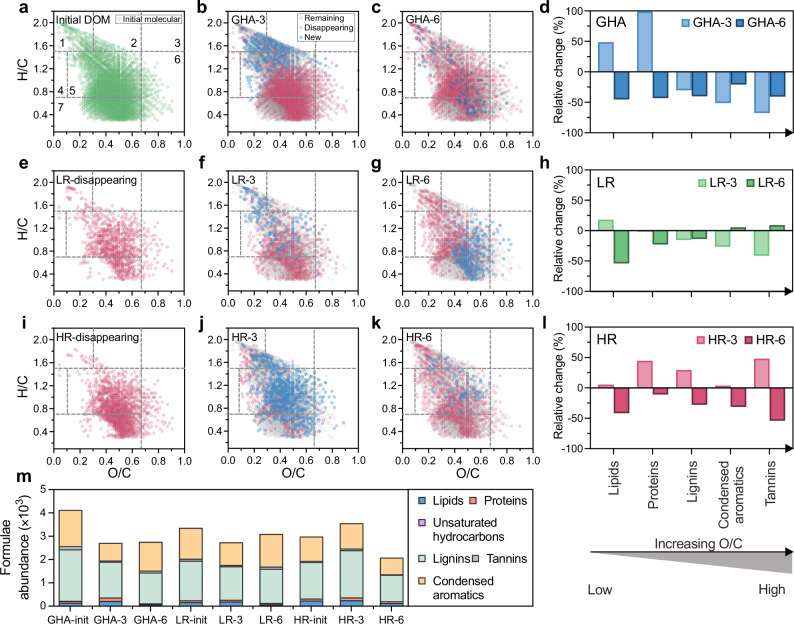
FT-ICR-MS results of the DOM solution after adsorption. van Krevelen diagrams of the initial DOM solution before adsorption (**a**) or removed molecular formulas in LR (**e**) and HR (**i**). The remaining, disappearing, and new formulas in GHA (**b**, **c**), LR (**f**, **g**), and HR (**j**, **k**). Formulas in the van Krevelen diagram are delineated into seven groups, including 1-lipids, 2-proteins, 3-carbohydrates, 4-unsaturated hydrocarbons, 5-lignins, 6-tannins, and 7-condensed aromatics. **d**, **h**, **l** Relative changes in chemical components between the initial DOM and the DOM after adsorption on goethite in GHA (**d**), LR (**h**), and HR (**l**). **m** Abundances of DOM formulas in each formula group after its adsorption in GHA, LR, and HR. Molecules belonging to the carbohydrates (group 3) were not detected in samples and are therefore not shown in plots **d**, **h**, **l**, and **m**. “-init” means initial DOM solutions before adsorption. “-3” and “-6” refer to the third and sixth samples obtained from the isothermal adsorption experiments (see Fig. 1g). Note that the FT-ICR-MS results represent the SPE-amenable and ESI-ionizable fraction of DOM rather than its total molecular composition. Source data are provided in the Source Data file.

Isothermal adsorption experiments revealed significant DOM molecular fractionation on goethite surfaces, as reported in previous studies (on goethite and other minerals)^25,33^. Fe (oxyhydr)oxides are expected to alter DOM’s electron exchange capacity via selective removal of DOM redox-active moieties (e.g., quinone/phenolic-like moieties)^16,25^. Recent findings by Liu et al.^17^ showed that Fe (oxyhydr)oxides slightly decreased DOM’s EDC but significantly enhanced its electron-accepting capacity (EAC). This shift would reduce the availability of electrons for Hg(II) reduction in goethite-Hg(II)-DOM ternary systems, contradicting the enhanced Hg(0) yields we observed. We hypothesize that Hg(II) may affect DOM fractionation during its adsorption on goethite. Indeed, Hg was observed to profoundly influence DOM adsorption in goethite-Hg(II)-DOM ternary systems (Fig. 2). LR groups showed a similar DOM adsorption pattern to that in the GHA system but a greater DOM adsorption. These results suggest that Hg(II) significantly enhanced DOM adsorption on goethite by forming an ionic bridge between DOM molecules and mineral surfaces^20^, which in turn altered DOM adsorption and fractionation in this system. HR systems exhibited distinct DOM adsorption behavior on goethite, with DOM adsorption saturation occurring at C_e-DOC_ > 4.17 mM (Fig. 1g), which is absent in LR and GHA systems. These findings indicate that, upon saturation of DOM thiol groups at high Hg concentration (e.g., in Hg-polluted soils), Hg may increasingly associate with oxygen-containing groups of DOM and reactive sites of mineral surfaces^34^. This competition for Hg attenuates the DOM-goethite ligand exchange^35^ and alters DOM conformation, thereby reducing DOM sorption in HR systems compared to LR systems.

To further understand the molecular mechanisms of the goethite-Hg(II)-DOM reactions, the original DOMs (in GHA, LR, and HR systems) and their fractionated components, including GHA-3 and GHA-6 derived from GHA, LR-3 and LR-6 derived from LR, and HR-3 and HR-6 derived from HR, were determined by FT-ICR-MS during the adsorption isotherm experiments (filled points in Fig. 1g). The molecular compositions of all DOM samples are summarized in Fig. 2a–l and Supplementary Table 2. Notably, DOM samples with and without Hg(II) clustered together in the analysis, indicating that Hg(II) had no significant impact on FT-ICR-MS detectability. Note that since solid-phase extraction (SPE) and negative electrospray ionization (ESI) were used in FT-ICR-MS analyses, this limits the detection to a selected subset of DOM components. Nevertheless, FT-ICR-MS remains a powerful tool for tracking molecular-level DOM changes during its interactions with metal ions and minerals, as previously described^36^. Molecular formulas were assigned based on isotopic patterns (^12^C vs ^13^C, ^32^S vs ^34^S), precise molecular mass, and Kendrick mass defect (KMD) analyses. No Hg-containing formulas were identified in the negative-ion mode, likely due to the positive charge imparted to DOM upon complexation with Hg^36^.

For the original DOM, 4137 mass peaks were assigned, comprising 54.3% CHO, 43.9% CHON, and 1.8% CHOS compounds (Supplementary Table 3). Molecular class distribution for all DOM samples is shown in Fig. 2m. Lignin-like compounds dominated across all systems, consistent with the typical composition of plant-derived DOM^37^. Elevated levels of condensed aromatic-like compounds likely reflect both the maturity of the peat-derived DOM^38^ and nitro-group enrichment under acidic conditions. van Krevelen diagrams (Fig. 2e, i) illustrate DOM components that disappeared or were selectively removed by reacting with Hg(II) before adsorption (i.e., formulas present in the original DOM but absent in Hg-containing LR/HR systems). These Hg-sensitive DOM formulas were predominantly lignin-like, tannin-like, and condensed aromatic-like molecules, and more of these molecules were removed with increasing Hg concentrations.

### DOM molecular fractionation in the Goethite-Hg(II)-DOM ternary system

Changes in the DOM molecular compositions following its adsorption on goethite revealed distinct fractionation patterns either with or without Hg(II). Figure 2a–m illustrates the molecular formulas and compositional changes in DOM after adsorption on goethite, highlighting which components were adsorbed (disappearing) and which remained in the supernatant (non-adsorbed). In the absence of Hg(II), DOM exhibited pronounced fractionation, with preferential removal of aromatic and polyphenolic compounds from solution. As the equilibrium DOC concentration increased, the number of molecules remaining in the DOM supernatant increased slightly, but with fewer lipid-like and protein-like compounds. This trend likely stems from hydrophobic interactions, as aliphatic compounds in hydrophobic fractions were preferentially adsorbed onto goethite^25^. In contrast, the presence of Hg(II) attenuates DOM fractionation on goethite, leaving more lignin-like and condensed aromatic-like components in the supernatant, particularly under the HR conditions. This finding is supported by shifts in relative abundances of O/C, H/C, DBE, and NOSC for molecular formulas in the initial and remaining DOM after adsorption on goethite (Supplementary Fig. 7).

These observations indicate that Hg(II) enhanced DOM adsorption on the goethite surface but decreased its molecular fractionation due to the formation of Hg-DOM complexes. Two primary mechanisms may explain this enhancement: a) Hg(II) acted as an ionic bridge, linking DOM to the goethite surface (DOM-Hg-goethite) and b) DOM adsorbed onto goethite in the form of Hg-DOM complexes (Hg-DOM-goethite). In the LR system, the dominant complexes were RS-Hg-OR and Hg(SR)_2_, with their formation limited by the available RSH groups on DOM. Consequently, most DOM adsorption onto goethite likely occurred via Hg(II) ionic bridging. In the HR system, however, Hg(OR)_2_ formation was favored, and could be adsorbed more readily and stably onto goethite than DOM alone, leading to the quick saturation of DOM adsorption on goethite under HR conditions. At elevated Hg(II) levels, Hg(II) preferentially complexes with DOM in solution rather than adsorbs directly onto goethite. Once DOM adsorption reached saturation, excess free Hg(II) was immobilized via the formation of Fe-[Hg-DOM complex]-Hg(II) structures on goethite. In LR-6 and HR-3, most newly formed DOM formulas matched those in the original DOM formulas (78.1% and 64.9%, respectively), suggesting the cleavage of weak Hg-OR bonds and subsequent release of OR-groups containing DOM ligands (e.g., phenols) back into the solution phase, where electron-donating functional groups remain available. This redistribution of DOM is consistent with the higher EDC measured for residual DOM in solution (Supplementary Fig. 6b). In the ternary goethite-Hg(II)-DOM system, residual DOM in solution exhibited higher EDC than that in the binary DOM-goethite system. Specifically, the LR-6 and HR-3 groups showed higher EDC values than GHA-3 and GHA-6, indicating that DOM remaining in solution retained a greater electron-donating capacity in the presence of Hg. This pattern suggests that Hg affects DOM fractionation during interaction with the mineral surface, leading to preferential retention of redox-active DOM components in the aqueous phase. Consistent with this interpretation, EDC values varied among treatments and showed trends that matched the molecular fractionation patterns observed by FT-ICR-MS (Fig. 2). Therefore, in the ternary (goethite-Hg(II)-DOM) system, residual DOM in solution exhibited higher EDC than that in the binary (DOM-goethite) system. This shift facilitated greater electron transfer to Hg(II) and thus enhanced Hg(0) production, consistent with our experimental results in both isothermal adsorption experiments and sequential kinetic experiments.

**Fig. 3:**
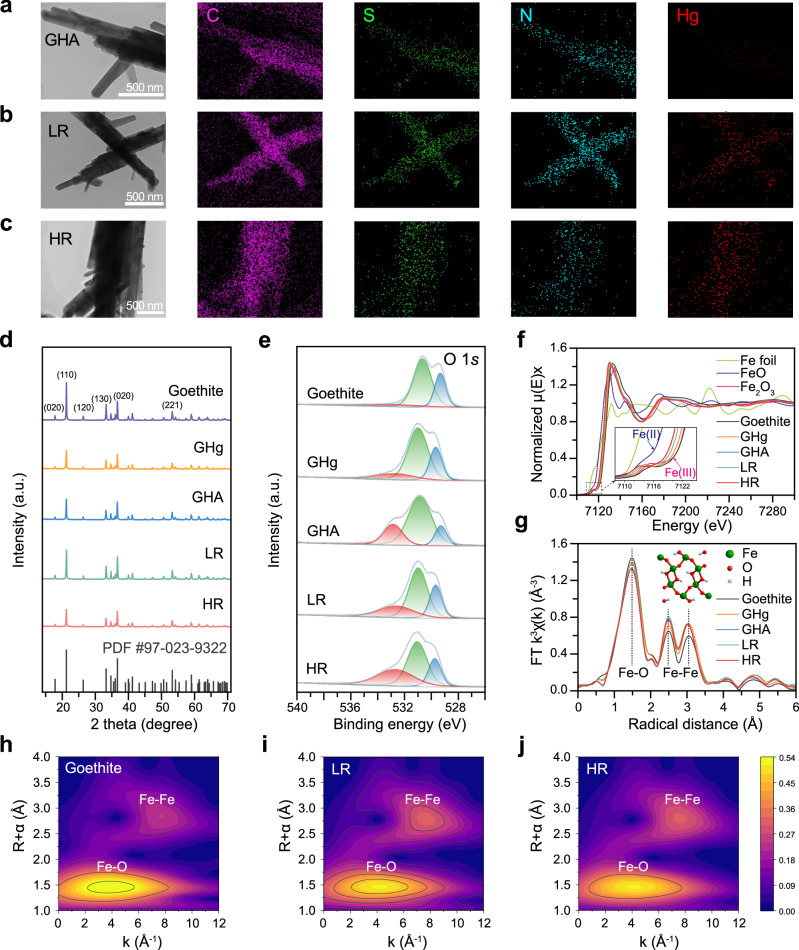
Characterization of goethite before and after adsorption. TEM and elemental mappings of goethite in GHA (**a**), LR (**b**), and HR (**c**). **d** XRD patterns of goethite before and after DOM and/or Hg adsorption. GHg represents the goethite with Hg(II) binary system. The observed diffraction peaks are consistent with the literature values for goethite (PDF #97-023-9322). **e** O 1 *s* XPS spectra of goethite before and after adsorption. **f** Fe K-edge XANES spectra of goethite and standard Fe foil, FeO, and Fe_2_O_3_ as references. **g** FT-EXAFS spectra (inset: the model for goethite). **h–j** WT-EXAFS of goethite before and after adsorption. The color scale represents the wavelet transform modulus intensity. Enlarged versions of **f** and **g** are provided in Supplementary Fig. 9.

We further characterized the mineral phases in the goethite-Hg(II)-DOM ternary systems to elucidate its impact on Hg and DOM fractionation (Fig. 3 and Supplementary Fig. 8). TEM images and EDS elemental mapping of goethite before and after adsorption (Fig. 3a–c) show that the goethite particles retained their characteristic acicular morphology, predominantly exposing (110)/(100) crystal planes with occasional (021) planes. EDS mapping revealed significantly higher surface coverage by Hg on Goe-HR compared to Goe-Hg and Goe-LR. The presence of Hg also correlated with enhanced surface concentrations of C, S, and N, agreeing well with our batch adsorption results and demonstrating increased adsorption of Hg (in HR systems) and DOM (in both LR and HR systems) in goethite-Hg(II)-DOM ternary systems. XRD analysis (Fig. 3d) confirmed the structural stability of goethite, with its diffraction patterns matching the standard goethite reference before and after adsorption. However, a notable reduction in the intensity of (110) and (020) diffraction peaks was observed for goethite in these goethite-Hg(II)-DOM ternary systems, demonstrating multi-faceted adsorption and possible surface dissolution^39,40^. High-resolution XPS analysis of the O 1 *s* spectra (Fig. 3e) identified three oxygen species across all samples: lattice oxygen (529.3 eV), hydroxy oxygen (531.9 eV), and surface-adsorbed oxygen (532.8 eV) species^41^. The adsorbed oxygen species, attributed to interactions between oxygen-containing functional groups and goethite surface hydroxyls, exhibited an inverse correlation with the surface hydroxyl group density (increased adsorbed DOM vs decreased surface hydroxyl groups)^25^. Notably, HR experiments yielded a significantly higher level of adsorbed oxygen than that in LR, reflecting a greater DOM load onto goethite with increasing Hg exposure levels.

The electronic structures and coordination environments of Fe in goethite are closely linked to the transformation of DOM and Hg in the ternary systems and were further investigated using SR-XANES and SR-EXAFS. Before adsorption, the Fe K-edge absorption spectrum of goethite closely matched that of reference Fe_2_O_3_. After adsorption, it shifted to an intermediate position between FeO and Fe_2_O_3_ (Fig. 3f), suggesting a partial reduction of Fe(III) to a mixed Fe(II)/Fe(III) regime. This interpretation is consistent with the measurable Fe(II) production in the ternary systems (Supplementary Fig. 4d). A distinct peak at 7114 eV along the axial direction confirmed the presence of an oxidized surface featuring Fe-O/-OH bonds. The Fourier-transformed k^2^-weighted EXAFS spectrum (Fig. 3g) of goethite displayed weakened peaks at ~1.5 Å after adsorption, suggesting a decrease in the average coordination number of Fe-O bonds, especially for the HR group. This observation was further supported by Fe K-edge wavelet transform (WT)-EXAFS analysis (Fig. 3h–j), which showed a decreased intensity at 3.8 Å^-1^ (characteristic of Fe-O bonds) following adsorption, confirming significant Fe(III) reduction in the goethite-Hg(II)-DOM ternary system. EXAFS curve fitting results (Table 1) indicated minimal changes in Fe-O bond length (from 2.00 to 2.01 Å), while the Fe coordination number decreased slightly (from 5.52 to 5.35) in these ternary systems. The absence of new Fe coordination environments suggests that Fe(III) reduction occurred via electron transfer from DOM, a mechanism consistent with previous studies showing that DOM adsorption can promote Fe release from mineral surfaces^42^. Specifically, DOM rich in oxygen-containing functional groups can act both as an electron donor and a shuttle, facilitating both abiotic and biotic reduction of goethite^43^. The formation of goethite-DOM complexes may further enhance Fe(III) dissolution by both lowering the redox potential of the Fe(III)/Fe(II) couple and mitigating surface passivation by Fe(II) products^43^. Although aqueous Fe(II) can directly reduce Hg(II), the net Hg(0) production correlated more strongly with EDC, suggesting that Fe(II) acted primarily as an indicator of coupled interfacial redox activity, rather than as the dominant electron donor, within the goethite-Hg(II)-DOM systems.

**Table 1:** EXAFS fitting parameters at the Fe K-edge for goethite samples

Sample	Shell	CN	*R* (Å)	*σ*^*2*^ (Å^2^)	∆*E* (eV)	*R*-factor
Goethite	Fe-O	5.52 ± 0.82	2.00 ± 0.02	0.0111 ± 0.0026	-1.45 ± 1.47	0.0087
GHg	Fe-O	5.45 ± 0.96	2.01 ± 0.02	0.0107 ± 0.0031	-2.51 ± 1.74	0.0069
GHA	Fe-O	5.35 ± 0.87	2.01 ± 0.02	0.0102 ± 0.0028	-2.17 ± 1.59	0.0061
LR	Fe-O	5.42 ± 0.71	2.01 ± 0.01	0.0103 ± 0.0022	-1.98 ± 1.31	0.0070
HR	Fe-O	5.36 ± 0.88	2.01 ± 0.02	0.0111 ± 0.0028	-2.26 ± 1.61	0.0131

### DFT simulations of molecular-level interactions

**Fig. 4:**
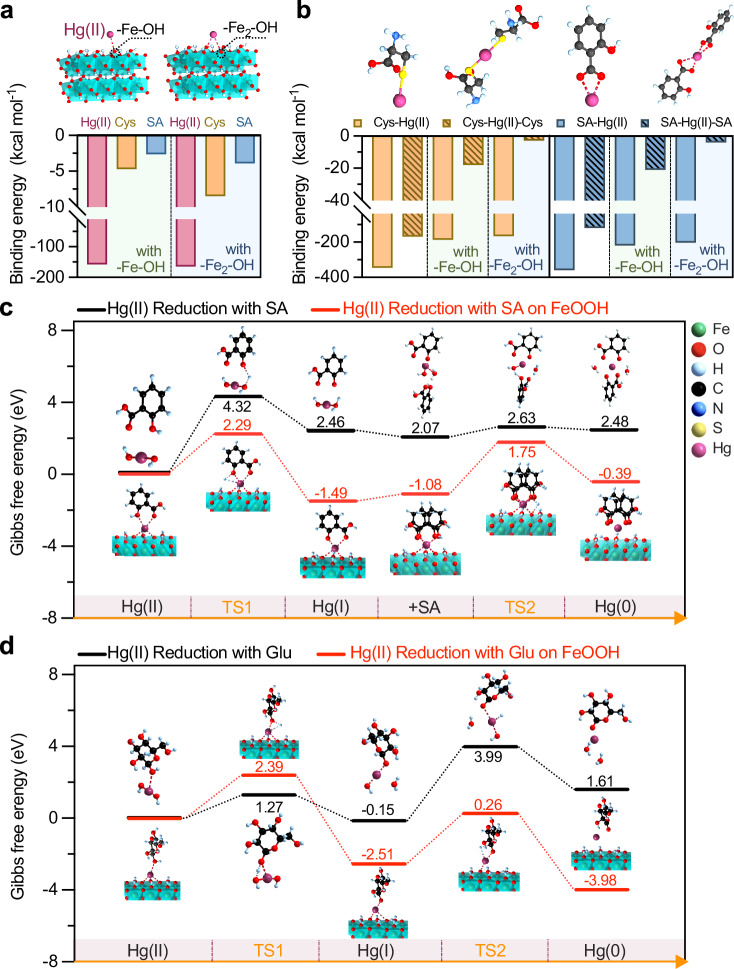
Results of DFT simulations. **a** Adsorption configurations and calculated binding energies for Hg(II), cysteine (Cys), and salicylic acid (SA) adsorption on goethite (FeOOH). **b** Molecular configurations and calculated binding energies of Hg(II) complexes with Cys or SA, and their binding energies on goethite. Two types of hydroxyl groups (-Fe-OH and -Fe_2_-OH) on the goethite surface were considered. Relative Gibbs free energy landscape for the reduction of Hg(II) to Hg(0) by SA (**c**) and glucose (Glu, **d**), with and without goethite. “TS” means transition state. “+SA” means binding to an additional SA molecule. Source data are provided in the Source Data file.

DFT simulations were subsequently performed to elucidate molecular-level interactions among Hg(II), DOM, and goethite in ternary systems, with a focus on thiol and carboxyl groups as primary adsorption sites. The goethite surface was modeled, including both singly (-Fe-OH) and doubly coordinated (-Fe_2_-OH) hydroxyl groups as potential Hg adsorption sites. Cysteine (Cys) and salicylic acid (SA) were selected as model compounds representing thiol- and carboxyl-containing functional groups, respectively. The use of Cys and SA allows specific Hg binding and reduction behaviors to be examined under well-defined conditions. However, these small molecules cannot represent the structural complexity of natural DOM, where steric constraints and cooperative interactions among multiple functional groups may influence binding strengths and reaction pathways. Consequently, binding energies derived from these model systems should be interpreted as reflecting simplified, functional-group-level interactions, and the associated reduction pathways may differ from those occurring within macromolecular DOM matrices. In this sense, results obtained from model compounds are intended to complement and help understand the observations made in complex DOM systems. Our calculations revealed significantly stronger binding energies for Hg(II) with DOM functional groups (-345.25 kcal mol^-1^ with Cys thiol; -359.20 kcal mol^-1^ with SA carboxyl) than that with goethite surface sites (-Fe-OH and -Fe_2_-OH) (Fig. 4a, b), indicating a higher affinity of Hg(II) for DOM in the ternary system. Interestingly, the resulting monodentate complexes, [Cys-Hg]⁺ and [SA-Hg]⁺, exhibited higher binding energies with goethite surface groups than with additional DOM molecules (Cys or SA), suggesting their preferential association with the goethite surface rather than with the second organic ligands. This would favor mineral-associated Hg-ligand interactions at the mineral interface. The stability of these ternary complexes likely also depends on environmental conditions such as pH, because shifts in protonation state can alter the balance between goethite-bound Hg-DOM species and dissolved DOM-bound Hg species. These interactions may in turn influence DOM fractionation patterns and modulate Hg reduction pathways.

Batch adsorption experiments revealed that saturation of DOM adsorption onto goethite was accompanied by increased formation of [Cys-Hg]^+^ and [SA-Hg]⁺ complexes under elevated Hg concentrations. The relatively weak binding of Hg with additional DOM ligands suggests that bidentate structures (e.g., Hg(OR)_2_, RSHgOR, and Hg(SR)_2_) may undergo rearrangement or dissociation into monodentate configurations, which could more readily associate with reactive surface sites on goethite. This mechanistic insight provides a plausible explanation for the formation of new molecular species observed in both LR and HR systems. Additionally, the partial degradation of DOM during adsorption may contribute to the emergence of molecular species differing from the original DOM. In short, these results suggest that Hg(0) production in the goethite-Hg(II)-DOM ternary system reflects the net balance between Hg(II) reduction and Hg(0) reoxidation. The enhanced net Hg(0) production observed in the presence of goethite likely stems from the combined contribution of both aqueous and surface-mediated processes that promote Hg(II) reduction. Redox-active DOM remaining in solution provides a sustained supply of electrons, while the co-adsorption of Hg and DOM at the goethite surface may create a favorable interfacial environment for reduction. Although Hg(0) reoxidation occurs, it does not offset the overall increase in net Hg(0) formation under the experimental conditions employed.

**Fig. 5:**
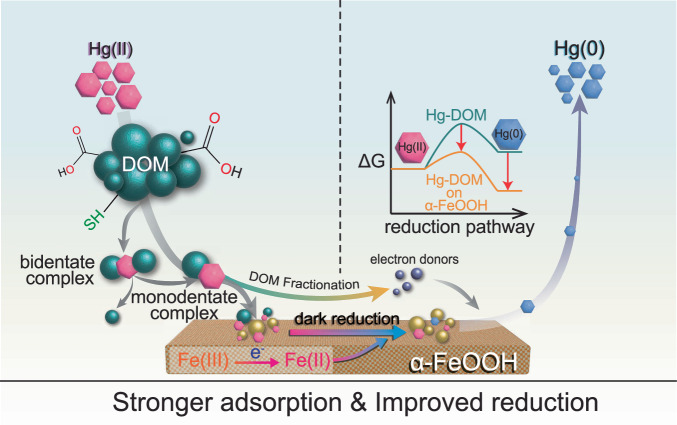
Conceptual diagram of adsorption of Hg(II) and DOM on goethite surface in the goethite-Hg(II)-DOM ternary system. This schematic illustrates the complexation, adsorption, and subsequent dark reduction pathways of Hg(II) in the ternary system.

In natural environments where some Hg(II) may first adsorb onto the mineral surface and then react with DOM, we also considered the reduction energy barriers for the transition from Hg(II) to Hg(0) in these ternary systems (Fig. 4c, d). Computational analyses of the transition states focused on three representative organic reductants (i.e., two SA molecules and one glucose (Glu) molecule) in reducing Hg(II) to Hg(0). The results revealed distinct energy barriers for different Hg(II) reduction scenarios. Specifically, unadsorbed Hg(II) in solution showed substantially higher reduction energy barriers: 4.32 eV for the reduction of Hg(II) to Hg(I) by SA and 4.14 eV for the subsequent reduction of Hg(I) to Hg(0) by Glu. However, the adsorbed Hg(II) on goethite showed significantly lower reduction energy barriers: 2.83 eV for SA and 2.77 eV for Glu, reducing Hg(II) to Hg(0) in their respective steps. These findings indicated that goethite adsorption facilitates Hg(II) reduction by organic compounds, lowering the activation energy by approximately 35–40% compared to free Hg(II) in solution-phase reactions. The enhanced reduction for the goethite adsorbed Hg(II) could be attributed to several factors: (1) stabilization of the transition states of Hg through surface interactions, (2) electronic modification of adsorbed Hg species, and (3) improved electron transfer pathways at the mineral interface. To integrate these results, Fig. 5 summarizes how goethite facilitates the interfacial processes that enhance net Hg(0) production in the ternary system. In this framework, the monodentate [Hg-DOM]^+^ complex exhibits both stronger adsorption onto goethite and more facile reduction kinetics, while the associated fractionation of DOM increases the EDC in the aqueous phase, ultimately leading to greater Hg(0) production. This dual effect of surface stabilization and catalytic promotion explains the observed enhancement of Hg(II) reduction in mineral-Hg(II)-DOM ternary systems.

### Pan-environmental significance of mineral-Hg(II)-DOM ternary systems on the global Hg cycle

**Fig. 6:**
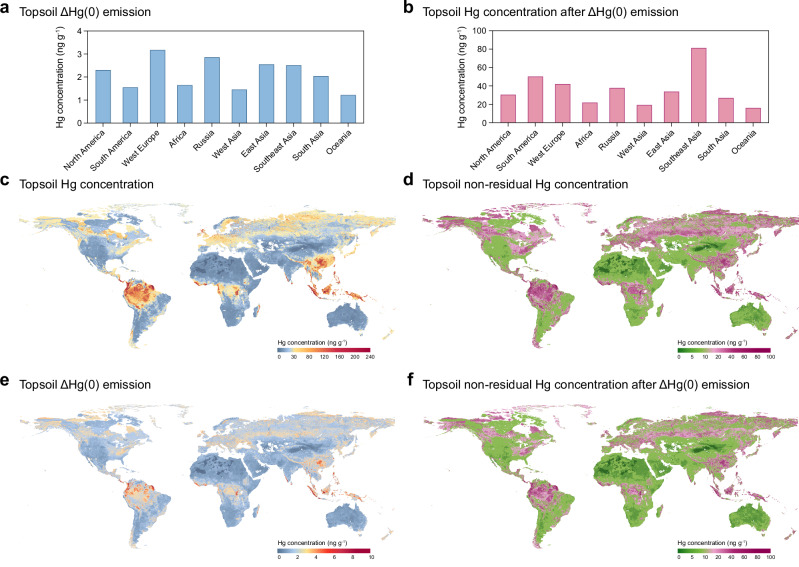
Evaluation of the impact of the ternary effect of goethite-Hg(II)-DOM on global topsoil (0–30 cm) Hg concentration. Mean ΔHg(0) emission (**a**) and post-emission Hg concentration (**b**) resulting from the ternary effect in different geographical regions. Spatial distributions of Hg concentration (**c**) and calculated non-residual Hg concentration in global topsoil (**d**). Spatial distributions of ΔHg(0) emission (**e**) and post-emission non-residual Hg concentration (**f**) resulting from the ternary effect. The topsoil Hg concentration map in **c** was derived from the published geospatial raster dataset of Guo et al.^45^ (available on Figshare under a CC BY 4.0 license) and used as the spatial input for this study. The distributions shown in **d–f** were calculated based on this dataset.

Iron oxyhydroxides and DOM exist ubiquitously in natural soil and water and are known to play pivotal roles in controlling the fate of pollutants (e.g., heavy metals) in aquatic ecosystems^44^. This study provides direct mechanistic evidence for enhanced Hg(II) reduction and net Hg(0) production due to coupled reactions in the goethite-Hg(II)-DOM ternary system. The relevance of this mechanism likely extends beyond soils to sediments and other surficial environments where minerals, DOM, and Hg coexist. In the present study, quantitative estimates were focused on topsoils, as these represent the zone of most active Hg exchange with the atmosphere. The experimental DOC concentrations used (0.83–8.75 mM DOC) encompass values typically found in natural topsoils (0.14–7.36 mM)^45^. The upper limit (8.75 mM) was selected to represent the equilibrium state of adsorption fractionation of DOM on goethite^25^. Global topsoils contain substantial amounts of Fe-bound organic carbon (291.7–1991.7 mM)^46^ and could thus provide abundant adsorption and reaction sites for geologically or anthropogenically derived Hg(II). This widespread geochemical setting provides a basis for estimating Hg(0) release from topsoils that is driven by the ternary interactions identified in this study. Batch experiments revealed that approximately 11% of non-residual Hg could be mobilized via coupled reactions in goethite-Hg(II)-DOM ternary systems, independent of DOM fractionation patterns (Supplementary Fig. 10). DFT simulations further showed catalytic roles of DOM-goethite interactions in promoting Hg(II) reduction. We acknowledge that the residual Hg fraction (*f* = 11%) was classified using a single total Hg threshold (0.25 µM), which represents a necessary simplification for the global scaling exercise. This approach is not intended to capture site-specific Hg speciation, which is known to be strongly influenced by local factors such as soil properties, organic matter composition, and redox conditions. Rather, the threshold is applied to distinguish broad differences in Hg retention behavior at a global scale, while recognizing the associated uncertainty. Based on these findings and recent global soil Hg concentration datasets (see Supplementary Text 7), we estimate global Hg(0) occurrence due to the dark reduction in mineral-Hg(II)-DOM ternary systems in surface soils (0–30 cm) as follows (Fig. 6). The estimated excess Hg(0) emission (ΔHg(0)) resulting from these ternary systems ranged from 0 to 0.06 nM (average: 0.010 ± 0.004 nM), accounting for 6.3 ± 1.6% of topsoil Hg across different geographical regions. Although these ΔHg(0) values are not particularly high, their cumulative impact could be substantial due to the pronounced disparity among Hg stocks in soils and the atmosphere^47^. This disparity suggests that Hg(0) production is sensitive to subtle variations in soil Hg content, DOM concentration, and mineral composition. Moreover, the experimentally observed Hg(0) reflects net accumulation, implying that the extent of Hg(II) reduction represented in this global scaling is likely conservative. Although spatial heterogeneity in soil compositions could introduce unavoidable uncertainty into these estimates, incorporating the ternary effects into Hg redox models, especially under dark conditions, is necessary. Recent studies using Hg isotopes to quantify soil Hg(0) release found that dark reduction through abiotic effects contributes ~25% of total Hg(0) in paddy soils and is crucial to Hg(0) re-emission from deeper forest soils^48,49^. Based on this work, we estimate that up to ~17% of Hg(0) production owes to the mineral-Hg(II)-DOM ternary system, highlighting the significance of these ternary systems to soil Hg(II) dark reduction.

These findings enhance our understanding of soil-air Hg exchange processes across natural ecosystems. The mineral-induced changes in DOM composition could further influence the photochemical transformation of Hg, since DOM photoreactivity, known to vary with molecular composition, is also affected by processes such as molecular fractionation and metal shielding, as demonstrated in earlier studies^50,51^. Furthermore, DOM-goethite-mediated reduction may decrease Hg bioavailability for methylation since adsorbed Hg on minerals and organic matter could potentially be methylated by microorganisms, as previously described^4^. Our research thus represents an important step forward in determining the availability of Hg species in the pan-environment, although natural DOM is highly heterogeneous, and the use of this single HA as a proxy represents an inherent limitation of this study. While this study specifically examined goethite, similar DOM fractionation behavior has been observed with other iron minerals, such as ferrihydrite, hematite, FeS, and FeS_2_, suggesting that ternary interactions may operate more broadly, depending on mineral properties and DOM characteristics^52^. Moreover, all experiments were conducted at pH 5.5, a mildly acidic condition representative of many Fe-rich weathered soils. This fixed pH defines a boundary condition of the present study, as pH can influence both goethite-DOM association and Hg partitioning between DOM and surface-adsorbed organic phases^22^. Moreover, the competition between Hg reduction and microbial methylation in the ternary system requires further investigation. Future field studies across different Fe minerals, DOM types, and soils/water bodies are also suggested to strengthen these findings and provide precise quantifications.

In summary, this study unveils an unexplored coupled process that enhances the dark reduction of Hg(II) in goethite-Hg(II)-DOM ternary systems, characterized by three key features: (1) the preferential adsorption of monodentate [Hg-DOM]^+^ complexes onto goethite, (2) increased retention of electron-donating DOM components in the aqueous phase, and (3) decreased energetic barriers for Hg(II) reduction at the solid-aqueous interface. These findings identify an additional route for Hg species transformations across terrestrial and aquatic environments, thereby advancing our understanding of natural Hg cycling and improving pan-environmental Hg cycle models. Ultimately, our research may lead to targeted mitigation strategies for Hg pollution through the regulation of key environmental conditions and support the implementation of the International Convention related to Hg (e.g., Minamata Convention).

## Methods

### Chemicals

All chemicals used in this study contained nondetectable amounts of Hg, as described in Supplementary Text 1.

### Adsorption experiments

In the ternary isotherm adsorption experiments, goethite was used at 2 g L^−1^, with DOM concentrations of 0.83–8.75 mM and Hg(II) (Hg(NO_3_)_2_) concentrations of 0.32–13.34 µM. The same concentration ranges were used in the corresponding binary control isotherm experiments, including the goethite-Hg(II) and goethite-DOM systems. All experiments were performed in triplicate for reproducibility and carried out in amber quartz vials to prevent light-induced reactions. All experiments were conducted under light-excluded conditions. Centrifuge tubes and related sample containers were shielded from light using amber materials or aluminum foil. Sample transfer and filtration were performed rapidly to minimize incidental light exposure. The pH was adjusted to 5.5 using 0.01 M NaOH and HNO_3_, representing typical acidic environmental conditions. Ultrapure water was used as the background solution for all adsorption experiments, and chloride was avoided throughout to minimize potential interference from Hg-Cl complex formation. To investigate the effect of Hg/DOC ratios on the sorption behavior of Hg(II) and DOM onto goethite, experiments were conducted at two Hg/DOC molar ratios: 0.0003 (lower ratio, LR) and 0.0013 (higher ratio, HR), representing conditions typically observed either in the natural or contaminated environment settings. The abundance of reduced sulfur sites in PPHA was estimated from the humic acid proportions reported by Skyllberg et al.^30^. Reduced organic sulfur (Org-S_red_) accounted for approximately 51% of total S in humic acid, and about 20–30% of Org-S_red_ was attributed to high-affinity S sites corresponding to thiol (RSH) groups with strong Hg binding capacity. Based on the elemental composition of PPHA (Supplementary Table 1) and the Hg/DOC molar ratio, the calculated Hg/RSH molar ratios for LR and HR are 0.42–0.62 and 1.80–2.20, respectively. A Hg/RSH molar ratio near 0.5 corresponds to the stoichiometry of two-coordination binding in Hg(SR)_2_ structure^26^. Thus, Hg mainly binds to thiol groups under LR, but more to O/N-containing functional groups under HR^36,53,54^.

Hg(II) and DOM were allowed to equilibrate for 8 h in the dark before reactions with goethite. Goethite was then added, and the suspensions (20 mL), including goethite, Hg(II), and DOM, were equilibrated on a rotary mixer at 50 rpm at 25°C for 24 h in the dark. Previous studies^14,25^, along with our kinetic measurements (Supplementary Fig. 1), showed that 24 h was sufficient for both DOM and Hg fractions to reach equilibrium on goethite. After 24 h, Hg(0) produced via dark reduction was first purged from the reactors using Hg-free argon (5 N) for 15 min and captured in a trapping solution, consisting of 40% (v/v) inverse aqua regia with an HCl:HNO_3_ ratio of 1:2^19^, before centrifugation and filtration of the suspensions. The ternary suspensions were then centrifuged at 15,550 × *g* for 5 min and filtered through a 0.22-μm pore size filter. A 5 mL aliquot of the filtrate was immediately treated with 0.2 M BrCl and stored at 4 °C for at least 12 h before Hg concentration measurements. The sequential kinetic experiments were carried out using the same procedures but differing orders of reactant additions: (i) DOM with Hg(II), then goethite, (ii) goethite with Hg(II), then DOM, (iii) goethite with DOM, then Hg(II), and (iv) DOM with Hg(II) (control). The concentrations of Hg(II) and DOM were 7 nM and 0.83 mM, respectively. Prior to the addition of the third reactant, the two initial reactants were pre-equilibrated for 2 h. This duration was sufficient for the relevant binary systems to reach equilibrium or a near-steady state, as supported by adsorption kinetics, Hg(0) production trends (Fig. 1e and Supplementary Fig. 1), and previous work on DOM fractionation^14^. Hg(0) produced in the kinetic experiments was analyzed by purging and trapping the vapor-phase Hg(0) onto a gold-coated sand trap, followed by thermal desorption under a flow of argon and detection by cold-vapor atomic fluorescence spectroscopy (CVAFS) (Model III, Brooks Rand, USA)^19^. Additional kinetic experiments were conducted by replacing goethite with magnetite and hematite at the same concentration. The experimental methods are consistent with those performed by the goethite group to compare the roles played by these three minerals in the ternary system.

The Hg concentrations in the Hg(0) trapping solution and the filtered supernatants were measured using cold atomic absorption spectrometry (CVAAS). The amount of adsorbed Hg was calculated by subtracting the measured concentrations of Hg(0) and residual Hg(II) in the supernatant from the initial total Hg concentration in solution. The concentration of dissolved organic carbon (DOC) in the filtered supernatants was determined using a total organic carbon (TOC) analyzer (TOC-L CPH, Shimadzu, Japan). The amount of DOM retained by goethite was calculated from the difference between the initial and equilibrium DOC concentrations in solution. After adsorption, solid samples were freeze-dried and characterized using powder X-ray diffraction (XRD, D8 ADVANCE, Bruker, Germany), X-ray photoelectron spectroscopy (XPS, ESCALAB 250Xi, Thermo Fisher Scientific Inc., USA), and field-emission transmission electron microscopy (TEM, HT-7700, Hitachi, Japan) equipped with energy-dispersive spectroscopy (EDS). The original XRD pattern of goethite is shown in Supplementary Fig. 2.

### FT-ICR-MS analysis

Selected filtered supernatants from the sorption experiments were analyzed using FT-ICR-MS. All samples were stored at 4 °C in the dark before pre-treatment and processed within one week to minimize time-dependent alterations in DOM molecular composition. Before analysis, solid phase extraction (SPE) was performed on all selected samples to desalinate and concentrate DOM using Agilent Bond Elute PPL cartridges (500 mg/6 mL)^55^. Specifically, the selected samples were first acidified with HCl to pH 2. Each cartridge was activated with 6 mL methanol and 6 mL acidified water (0.01 M HCl, pH 2). Then, the acidified samples were loaded onto the cartridges at a flow rate of 5 mL min^−1^. After sample loading, the cartridges were rinsed with 30–50 mL acidified water to remove salts and other impurities. DOM retained on the cartridges was eluted with 6 mL methanol. The eluates were concentrated under a gentle nitrogen stream and then reconstituted with ultrapure water to a final concentration of 8.33 mM (100 mg_C_ L^−1^) in 50% methanol (v/v). All samples were ionized using electrospray ionization (ESI) in negative mode. Additional details of FT-ICR-MS analyses and molecular formula assignments are provided in Supplementary Texts 2–4. The results of FT-ICR-MS analyses can be downloaded from the Figshare website (10.6084/m9.figshare.29095691).

### Chemical analyses

In this study, EDC in the supernatant after adsorption was quantified using a decolorization assay based on the reduction of the ABTS^•+^ radical cation (2,2’-azino-bis[3-ethylbenzothiazoline-6-sulfonate]) adapted from Walpen et al.^56^ and Zhu et al.^57^, with modifications to match our adsorption experiments. All determinations were performed at pH 5.5 (2-Morpholinoethanesulphonic acid, MES), consistent with the reaction conditions in our study. Acetate buffer was avoided because acetate can complex Hg(II), potentially affecting Hg(II) speciation and influencing the results. We additionally calibrated the molar absorptivity of ABTS^•+^ at pH 5.5 to ensure accurate quantification of EDC under our experimental conditions (Supplementary Fig. 11). Additional details and analytical methods are given in Supplementary Text 5. Ferrous iron (Fe(II)) concentrations were determined by the 1,10-phenanthroline method^58^. Samples were mixed with hydroxylamine hydrochloride (1.44 M), 1,10-phenanthroline (6.7 mM), and acetate-ammonium acetate buffer (pH 4.5), and absorbance at 510 nm was measured after 15 min. Fe(II) concentrations were calculated from freshly prepared Fe(II) standards after reagent blank correction. All measurements were performed in triplicate.

### SR-XAFS measurements

Hg L_III_ edge X-ray absorption near edge spectroscopy (XANES) analyses were performed at the XAS beamline BL14W1 of the Shanghai Synchrotron Radiation Facility (SSRF). Spectra were obtained from four freshly prepared solutions/suspensions from the above sequential experiments (i–iv). Each sample was prepared in advance according to the beamtime schedule to ensure the intended reaction stage was reached at the time of analysis. All samples were kept under strict dark conditions before measurement. To avoid Fe interfering with the signal of Hg, 0.1 g L^−1^ goethite, 1 mM DOM, and 5 mM Hg(NO_3_)_2_ were used in the experiments. Synchrotron radiation X-ray absorption fine structure (SR-XAFS) measurements at the Fe K-edge were obtained at the beamline BL14W1 of the SSRF, operating at 3.5 GeV in “top-up” mode at a constant current of 220 mA. The Athena and Artemis codes were employed for XAS data extraction and profile fitting. Additional details and analytical methods are given in Supplementary Text 6.

### DFT calculations

Binding energies of Hg(II) with DOM functional groups and Hg-DOM complexes with goethite were calculated using the CASTEP module in Materials Studio 2018. The generalized gradient approximation (GGA) with the Perdew-Burke-Ernzerhof (PBE) exchange-correlation functional was employed to model electronic interactions. Ultrasoft pseudopotentials and a plane-wave basis set with a 500-eV kinetic energy cutoff ensured accurate energy convergence. To simplify DOM modeling, salicylic acid (SA) and cysteine (Cys) were selected as representative molecules: SA to simulate carboxyl and phenolic hydroxyl groups, while Cys represented thiol, amino, and carboxyl functionalities, capturing key adsorption sites in DOM. To investigate the reaction energy barriers of ligand-mediated Hg(II) reduction in the ternary systems, SA and glucose (Glu) were investigated as reductants. Transition state searches were conducted to calculate the energy barriers and Gibbs free energy changes for the reduction of Hg(II) to Hg(0). Further details are provided in Supplementary Text 7.

### Spatial analysis

The global spatial distribution of Hg in topsoil (0–30 cm) was obtained from the publicly available geospatial raster dataset reported in Guo et al^45^. This dataset integrates the topsoil subset of a large field-observation database (*n* = 11,747) with environmental covariates at a relatively high spatial resolution (0.1° × 0.1°). We used the published 0–30 cm raster layer directly, and all subsequent spatial analyses were based on this dataset. A more detailed description of the dataset is provided in Supplementary Text 1. QGIS (version 3.34) was used to generate a global map of Hg concentration in surface soil. The Hg(0) emission attributable to the goethite-Hg(II)-DOM ternary effect (ΔHg(0)) was estimated using the Eq. (1):

1 $$\Delta {{{\rm{Hg}}}}(0)={C}_{{{{\rm{Hg}}}}}\times (1-f)\times {R}_{\Delta {{{\rm{Hg}}}}(0)}$$

where *C*_Hg_ is the soil Hg concentration, *f* represents the fraction of residual Hg in soil, *R*_ΔHg(0)_ is the coefficient of Hg(0) production driven by the goethite-Hg(II)-DOM ternary interaction as determined in this study. Residual Hg is sulfide-bound Hg (*α*-HgS and *β*-HgS). We defined the ‘non-residual Hg’ as all extractable Hg, including Hg bound to DOM, weakly sorbed, and carbonate-bound Hg species. These non-residual Hg are labile and bioavailable, and we hypothesize that these Hg species can participate in goethite-Hg(II)-DOM reactions. Previous studies indicate that residual Hg comprises approximately 70% in Hg-contaminated soils and 30% in uncontaminated soils^59,60^. Major Hg-contaminated regions include Southeast Asia, Central Africa, and North-South America, where the average topsoil Hg concentration exceeds 0.25 µM^47^. Accordingly, soils with Hg concentrations above 0.25 µM were classified as contaminated (*f* = 70%), and those below as uncontaminated (*f* = 30%). The final Hg concentration database was constructed using 0.1° × 0.1° grid cells. Regional ΔHg(0) values were also calculated based on the average topsoil Hg concentration with the same criteria. The database can be downloaded from the Figshare website (10.6084/m9.figshare.29095691).

## Supplementary information

Supplementary Information

Peer Review file

## Source data

Source data

## Data Availability

The global data generated in this study have been deposited in the Figshare database under accession code 10.6084/m9.figshare.29095691. All other data discussed in the paper are available in the paper, the Supplementary Information file, and the Source Data file. Source data are provided with this paper.
