# Supplementary material for: An unexplored coupling process enhances dark Hg(II) reduction in mineral-Hg(II)-DOM ternary systems
Source: Nat Commun. 2026 Apr 24;17:6554. doi: 10.1038/s41467-026-72424-6 (PMC13381896; doi:10.1038/s41467-026-72424-6)
Supplement: Supplementary file 1 — Supplementary Information [file 41467_2026_72424_MOESM1_ESM.pdf]

Supporting Information for

An unexplored coupling process enhances dark Hg(II) reduction in mineral-Hg(II)-DOM ternary systems

Ruiyang Sun[1,2], Guoming Lin[3,], Yanping Li[1,2], Jinhang Wang[1,2], Ziyin Liu[1,2], Runsheng Yin[4], Ruoyu Sun[5], Lizhong Zhu[1,2,6], Jiating Zhao[1,2,6*], Baohua Gu[7]

[1] State Key Laboratory of Soil Pollution Control and Safety, Zhejiang University, Hangzhou 310058, China.
[2] Zhejiang Provincial Key Laboratory of Organic Pollution Process and Control, Hangzhou 310058, China.
[3] State Key Laboratory of Green and Efficient Development of Phosphorus Resources & School of Future Technology, Fuzhou 350108, China.
[4] State Key Laboratory of Critical Mineral Research and Exploration, Institute of Geochemistry, Chinese Academy of Sciences, Guiyang 550081, China.
[5] Institute of Surface-Earth System Science, School of Earth System Science, Tianjin University, Tianjin 300072, China.
[6] ZJU-Hangzhou Global Scientific and Technological Innovation Center, Hangzhou 311200, China.
[7] Environmental Sciences Division, Oak Ridge National Laboratory, Oak Ridge, TN 37831, USA.
*Corresponding author: zhaojt@zju.edu.cn

This file includes:

Supplementary Figures 1 to 11
Supplementary Tables 1 to 3
Supplementary Text 1 to 8
Supplementary References

Supplementary Figures

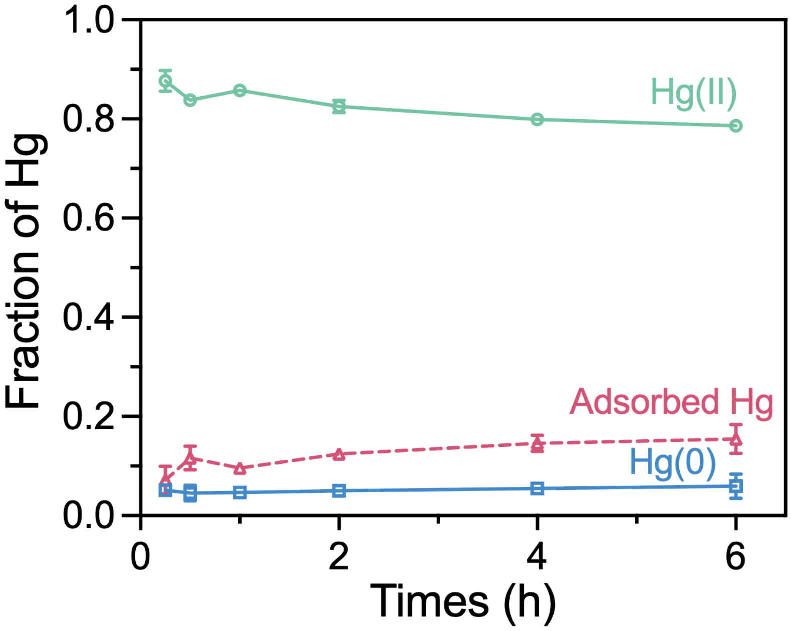

Supplementary Fig. 1 Adsorption of Hg(II) on goethite as a function of time (initial DOC concentration: 1.67 mM; Hg/DOC molar ratio: 0.0013). The results are represented as means ± s.d., n = 3. Source data are provided in the Source Data file.

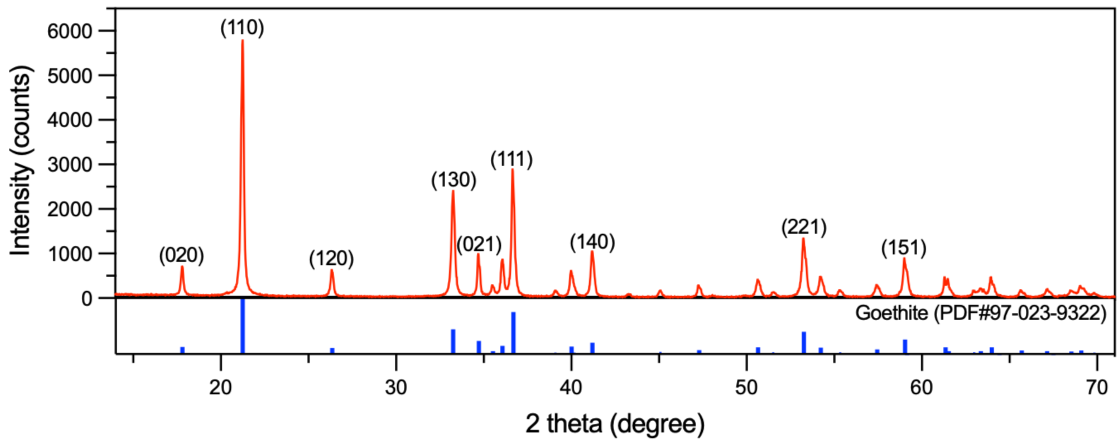

Supplementary Fig. 2 XRD pattern of goethite used in adsorption experiments. The observed diffraction peaks are consistent with the literature values for goethite (PDF #97-023-9322).

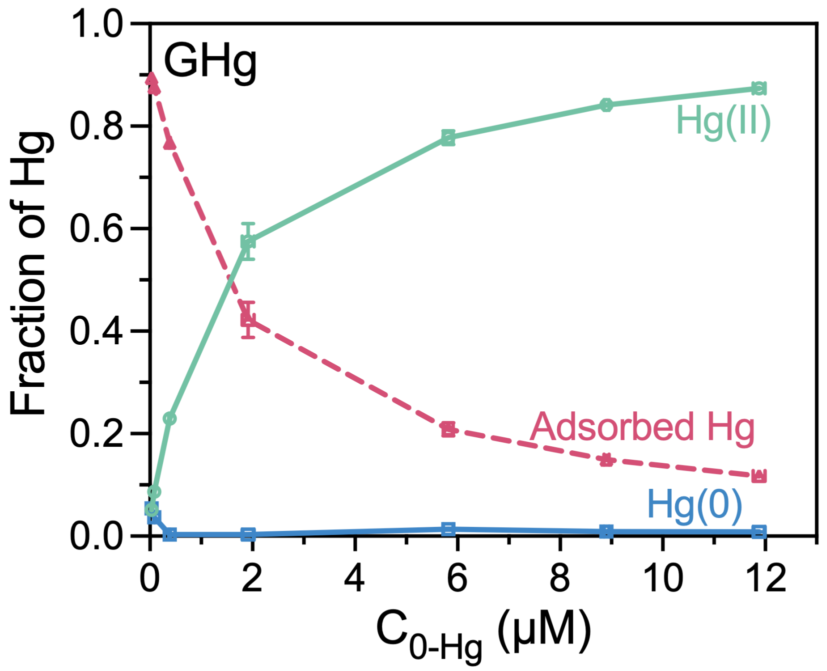

Supplementary Fig. 3 Hg species changes in GHg isothermal adsorption experiments. GHg serves as a control experiment of Hg(II) and goethite, without DOM. The graph is represented as means ± s.d., *n* = 3. Source data are provided in the Source Data file.

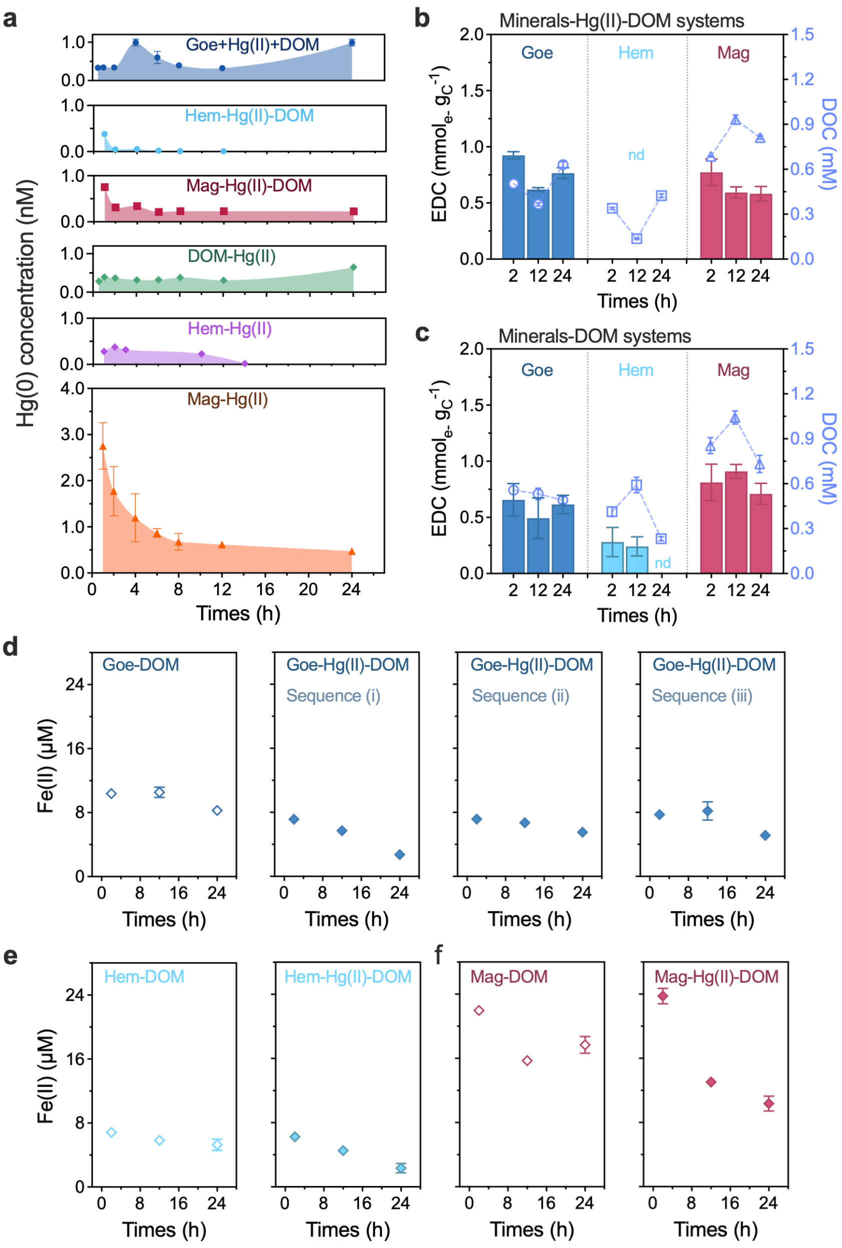

Supplementary Fig. 4 Production of Hg(0) and chemical changes of supernatant in mineral contrast kinetics experiments. a Hg(0) production in mineral-Hg(II)-DOM ternary systems in which goethite (Goe), magnetite (Mag), and hematite (Hem) participate, respectively. Experiments without the addition of DOM served as controls to show the reduction capacity of magnetite and hematite for Hg(II). Experiment without mineral (DOM-Hg(II)) shows the reduction of Hg(II) mediated by DOM. The initial concentrations of DOM and Hg(II) are the same as those in the kinetic experiments as described in Methods. Additions of minerals remain consistent. The presence of Fe(II) in magnetite enables direct reduction of Hg(II), resulting in more production of Hg(0). In the ternary systems, Hg(0) production with magnetite was comparable to that of DOM-Hg, whereas hematite showed a significant inhibitory effect on Hg(II) reduction with much lower Hg(0) production. **b, c** EDC and DOC of the residual DOM in the supernatants at 2, 12, and 24 h in the mineral-Hg(II)-DOM ternary systems (**b**) and the mineral-DOM binary systems (**c**). EDC is calculated from the slope of the linear relationship between $ABTS^{\bullet+}$ loss and DOC concentration, and the error bar was calculated as the standard error of the fitted slope. nd means not detected. **d–f** Fe(II) concentration of the residual DOM in the supernatants at 2, 12, and 24 h in three mineral-DOM binary systems and the mineral-Hg(II)-DOM ternary systems. All graphs are represented as means ± s.d., *n* = 3. Source data are provided in the Source Data file.

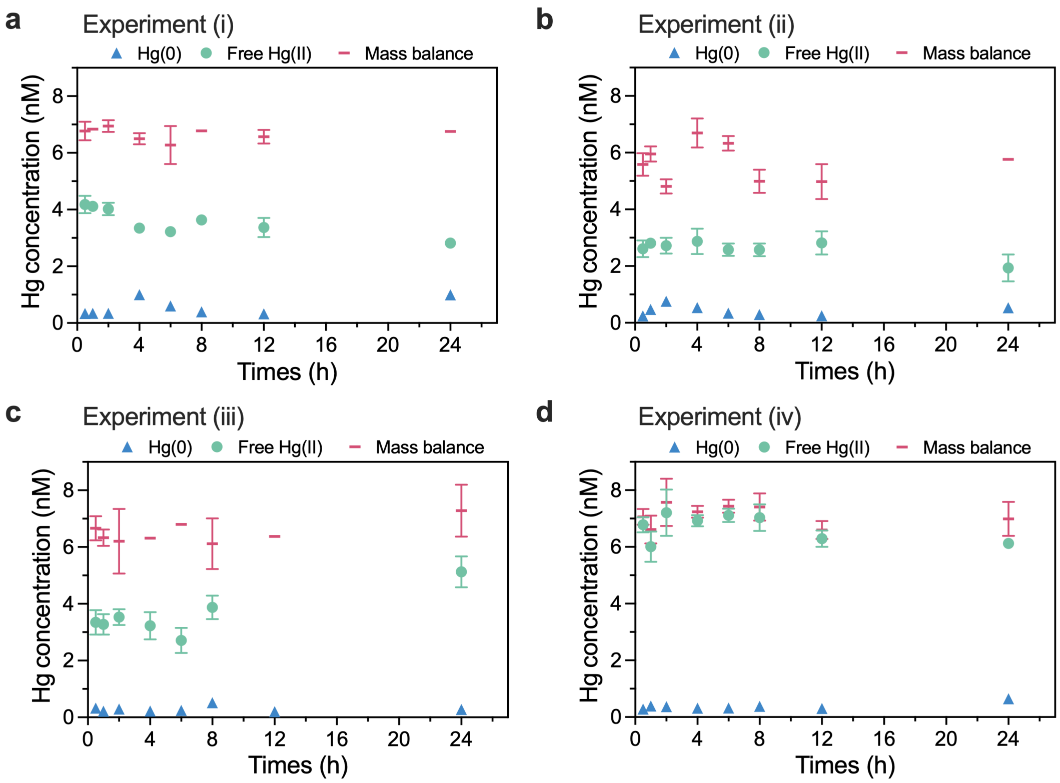

Supplementary Fig. 5 Hg concentration of Hg species over time in four sequential addition kinetic experiments. a experiment (i), DOM with Hg(II), then goethite. **b** experiment (ii), goethite with Hg(II), then DOM. **c** experiment (iii), goethite with DOM, then Hg(II). **d** experiment (iv), DOM with Hg(II). Compared with experiments (i) and (iv), the production of Hg(0) was increased by an average of 27.8% over 24 hours, indicating that goethite promoted the dark reduction of Hg(0). All graphs are represented as means ± s.d., $n = 3$.

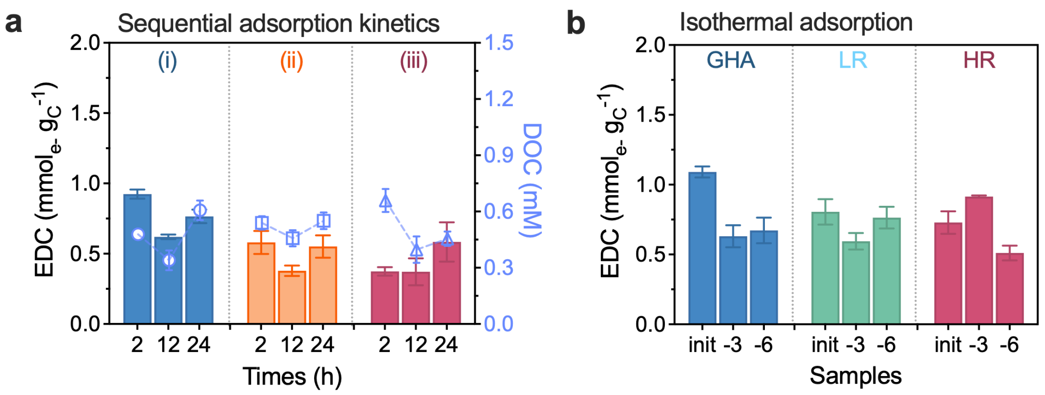

Supplementary Fig. 6 Chemical changes of supernatant in sequential adsorption kinetics experiments and isothermal experiments. a EDC and DOC of the residual DOM in the supernatants of goethite-Hg-DOM ternary systems at 2, 12, and 24 h in the sequential kinetic experiments. **b** EDC of residual DOM in the supernatants from selected isothermal adsorption experiments, corresponding to the same samples analyzed by FT-ICR-MS. "init" means initial DOM solutions before adsorption. EDC is calculated from the slope of the linear relationship between $ABTS^{\bullet+}$ loss and DOC concentration, and the error bar was calculated as the standard error of the fitted slope. "-3" and "-6" refer to the third and sixth samples obtained from the isothermal adsorption experiments. All graphs are represented as means ± s.d., n = 3. Source data are provided in the Source Data file.

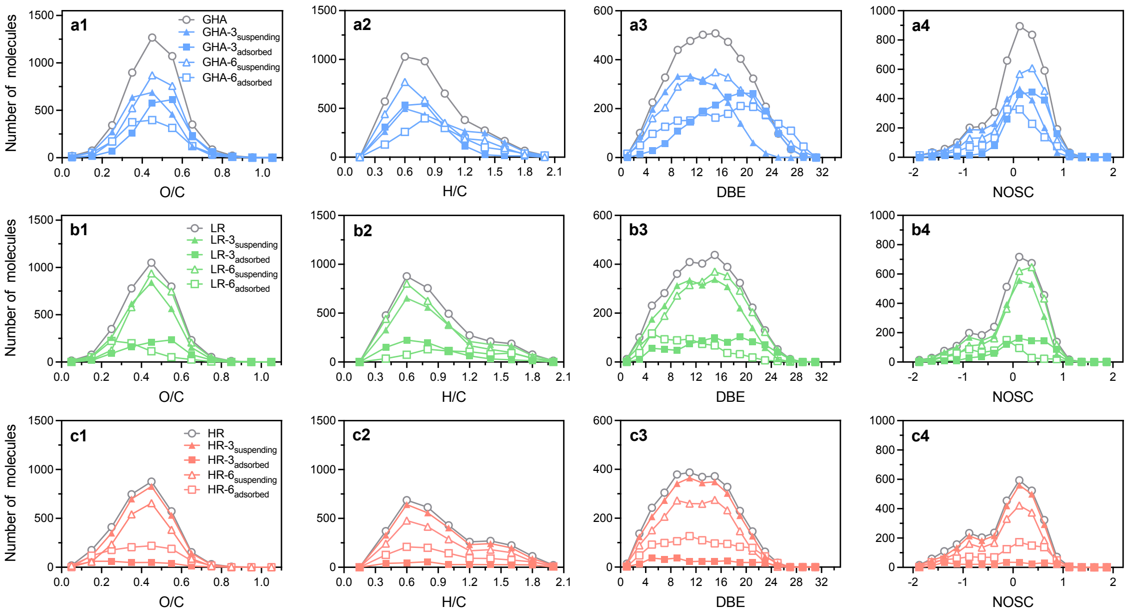

Supplementary Fig. 7 Quantitative distributions of molecular O/C, H/C, DBE, and NOSC in GHA, LR, and HR before and after adsorption on goethite. Distributions of O/C (**1**), H/C (**2**), DBE (**3**), and NOSC (**4**) are shown for molecules in GHA (**a**), LR (**b**), and HR (**c**). subscript$_{suspension}$ denotes molecules of DOM remaining in solution after adsorption; subscript$_{sequestered}$ denotes molecules of DOM sequestered by goethite. Source data are provided in the Source Data file.

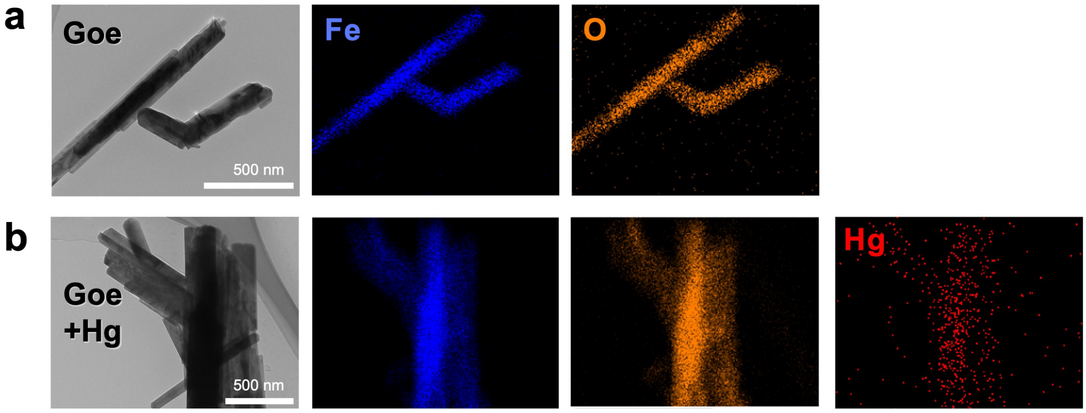

Supplementary Fig. 8 TEM and elemental mappings of goethite before (a) and after Hg adsorption (b).

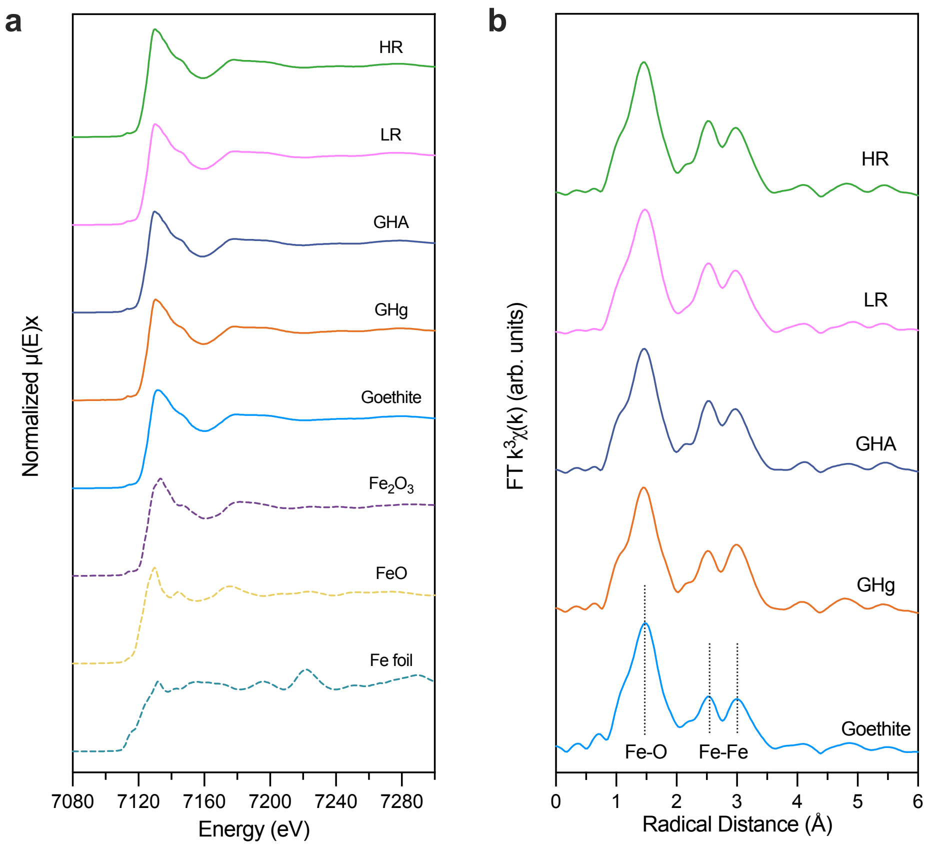

Supplementary Fig. 9 Enlarged versions of Fe K-edge XANES spectra of goethite and standard Fe foil, FeO, and Fe_2O_3 as references (a) and FT-EXAFS spectra of goethite before and after adsorption (b).

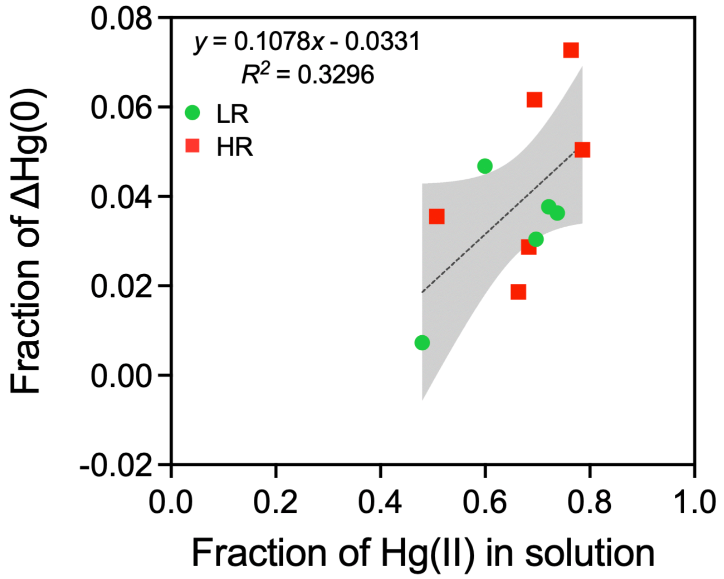

Supplementary Fig. 10 Fraction of ΔHg(0) versus Hg(II) remaining in solution after adsorption on goethite for all samples. The light gray shaded areas denote the 95% confidence intervals. The slope (~0.11) was applied for the spatial analysis.

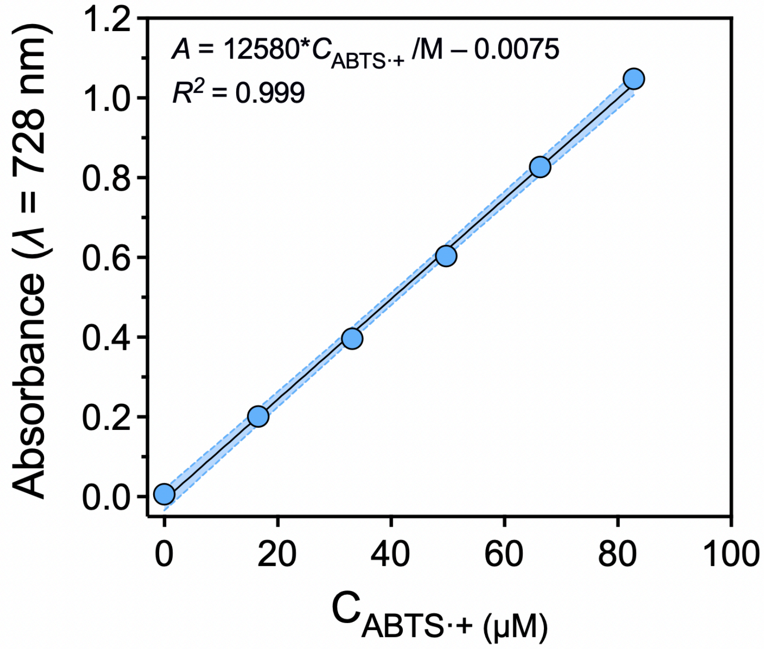

Supplementary Fig. 11 Determination of the molar absorption coefficient of ABTS$^{\bullet+}$ at a wavelength $\lambda = 728$ nm (pH 5.5, $n = 3$). The molar absorption coefficient of ABTS$^{\bullet+}$ was $\varepsilon = 12580$ M^{-1} cm^{-1}. Shading depicts 95% confidence intervals on the slope.

Supplementary Tables

Supplementary Table 1 Elemental compositions of PPHA (1S103H).

Standard Material	C %	H %	O %	N %	S %	P %
PPHA	56.37	3.82	37.34	3.69	0.71	0.03

Data source: https://humic-substances.org/elemental-compositions-and-stable-isotopic-ratios-of-ihss-samples/.

Supplementary Table 2 H/C_w, O/C_w, double bond equivalent (DBE_w), aromaticity index (AI_w), nominal oxidation state of carbon ($NOSC_w$), and molecular weight (MW) of selected DOM samples.

DOM samples	H/C_w	O/C_w	DBE_w	AI_w	$NOSC_w$	MW
HA	0.75	0.46	14.12	0.55	0.14	434.56
GHA-3	1.03	0.41	10.05	0.39	-0.27	386.87
GHA-6	0.81	0.46	13.49	0.52	0.06	428.39
LR-init	0.79	0.44	13.23	0.54	0.06	410.58
LR-3	0.90	0.42	11.66	0.48	-0.10	391.80
LR-6	0.72	0.47	14.43	0.57	0.18	434.44
HR-init	0.90	0.40	11.74	0.48	-0.14	391.98
HR-3	0.93	0.42	11.37	0.45	-0.13	396.39
HR-6	0.93	0.42	11.15	0.45	-0.15	387.43

DOM sample HA is the initial DOM solution (PPHA) used in all adsorption experiments. "-init" means initial DOM solutions before adsorption. "-3" and "-6" refer to the third and sixth samples obtained from the isothermal adsorption experiments (see Fig. 1g).

Supplementary Table 3 Number of the detected compounds in the original DOM sample.

DOM Sample	CHO	CHON	CHOS
original DOM (HA)	2246 (54.3%)	1816 (43.9%)	75 (1.8%)

This distribution diverges from earlier reports[1], which may reflect methodological differences, including the use of a finer 0.22-μm filter (to inhibit microbial activity) and trace HNO_3 additions (for pH adjustment). The nitric acid environment likely promoted nitro-group incorporation, particularly into condensed aromatic structures[2,3], thereby enhancing molecular diversity. While functional group additions could theoretically reduce DOM hydrophobicity and alter goethite adsorption, HNO_3 usage was rigorously minimized in this study. Although some HNO_3-induced artifacts are inevitable, their comparative impact across experimental groups is assumed to be negligible.

Supplementary Text

Supplementary Text 1 Materials and reagents

Goethite, magnetite, and hematite were obtained from Macklin Biochemical Co., Ltd., Shanghai, China. Sodium hydroxide (NaOH, ≥ 96%), nitric acid (HNO_3, analytical reagent grade), hydrochloric acid (HCl, analytical reagent grade), sulfuric acid (95%, w/w), and sodium hypochlorite solution (6–14% active chlorine basis) were purchased from Hushi Chemical Co., Ltd., Shanghai, China. Potassium bromate ($KBrO_3$, 99.8%), potassium bromide (KBr, 99%), and Tin(II) chloride dihydrate ($SnCl_2{\cdot}2H_2O$, ≥ 99.99%), 2,2'-azino-bis(3-ethylbenzothiazoline-6-sulfonic) diammonium salt (ABTS, ≥ 98.0%), 2-morpholinoethanesulphonic acid (MES), 1,10-phenanthroline (≥ 98.0%), hydroxylamine hydrochloride (≥ 99.99%), ammonium acetate (≥ 97.0%) were purchased from Aladdin Biochemical Technology Co., Ltd., Shanghai, China. Ultrapure water was used for all solution preparations. Pahokee peat humic acid (PPHA, 1S103H) was purchased from the International Humic Substances Society (IHSS) and used to prepare the DOM solution by dissolving it in 0.1 M NaOH. Major elemental compositions in PPHA are given in Supplementary Table 1, with the concentration of C and S being 56.37 wt.% and 0.71 wt.%, respectively. The DOM solution was filtered using a 0.22-μm polyethersulfone (PES) filter and then stored in the dark at 4°C. Freshly prepared DOM solutions were used for all batch experiments. Hg(II) solutions were prepared by diluting the SRM NIST 3133 Hg standard solution (50 mM $Hg(NO_3)_2$ in 10% HNO_3). Bromine monochloride (BrCl) solution was used to mineralize DOM before measuring Hg concentrations, while stannous (II) chloride ($SnCl_2$) was used as a reductant in Hg measurements. Both BrCl and $SnCl_2$ solutions were prepared following the U.S. Environmental Protection Agency (EPA) method 1631.

Supplementary Text 2 FT-ICR-MS Analysis Methods

The samples were introduced via a syringe pump at an infusion rate of 200 μL h^{-1}. The spectra were acquired from *m/z* 150 to *m/z* 1000 with a transient size of 4 mega words, and 256 scans were accumulated for each mass spectrum using a 7-T SolariX 2xR FT-ICR-MS (Bruker Daltonik GmbH, Bremen, Germany). The sweep excitation power was 18% for all measurements in this study. The conditions of the nebulizer were as follows: gas pressure 1.8 bar, drying gas temperature 200°C, drying gas flow rate 5.0 L min^{-1}, and ion accumulation time 0.030 s. Standard Suwannee River fulvic acid (SRHA, from IHSS) was used for quality control and calibration for all spectra[4], and the same instrument parameters were used for all samples to ensure analytical consistency.

Supplementary Text 3 Molecular Formula Assignment

Molecular formulas were assigned using the Formula Calculator software, with the condition that the mass error between the measured and calculated mass for a given chemical formula was less than 0.6 ppm. The generated formulas were validated by applying sensible chemical constraints: O/C ≤ 1, 0.3 ≤ H/C ≤ 2, N/C ≤ 0.5, S/C ≤ 0.2, double-bond equivalence (DBE) ≥ 0, element counts: ≤ 80, H unlimited, C ≤ 60, N ≤ 2, S ≤ 2, in conjunction with an automated theoretical isotope pattern comparison. Only elemental compositions in the range of −10 ≤ [DBE-O] ≤ +10 were considered. Kendrick mass (KM) and Kendrick mass defect (KMD) were calculated to identify the CH_2-homologous molecular formulas series[5]. The elemental compositions were based on previously reported values for similar types of organic matter[1].

The following parameters were calculated for data analysis, with equations provided in Supplementary Text 4: DBE; aromaticity index (AI)[6], nominal oxidation state of carbon (NOSC)[7], and magnitude-weighted average parameters, including the numbers of C, H, O, N, as well as O/C_w, H/C_w, DBE_w, $NOSC_w$, and AI_w for each sample. The assigned formulas were grouped into seven molecular groups based on their distributions in the van Krevelen diagram[8], which are lipids (1.5 < H/C ≤ 2, 0 < O/C ≤ 0.3), proteins (1.5 < H/C ≤ 2.2, 0.3 < O/C ≤ 0.67), carbohydrates (1.5 < H/C ≤ 2.2, 0.67 < O/C ≤ 1.2), unsaturated hydrocarbons (0.7 < H/C ≤ 1.5, 0 < O/C ≤ 0.1), lignins (0.7 < H/C ≤ 1.5, 0.1 < O/C ≤ 0.67), tannins (0 < H/C ≤ 1.5, 0.67 < O/C ≤ 1.2), and condensed aromatics (0.2 < H/C ≤ 0.7, 0 < O/C ≤ 0.67).

Supplementary Text 4 Calculations and parameters used in FT-ICR-MS Data Processing

The Kendrick mass defect (KMD) analysis is determined from Eqs. (1) and (2), and compounds of the same -CH_2 homologous series will have identical KMD.

$$\text{Kendrick mass} = \text{IUPAC mass} \times (14/14.01565) \quad (1)$$

$$\text{KMD} = \text{nominal Kendrick mass} - \text{exact Kendrick mass} \quad (2)$$

The weighted mean molecular indices of the formulas, e.g., H/C, O/C, double bond equivalent (DBE), aromaticity index (AI), and nominal oxidation state of carbon (NOSC), were calculated using the equations provided below. In these equations, *C*, *H*, *O*, *N*, and *S* represent the stoichiometric numbers of carbon, hydrogen, oxygen, nitrogen, and sulfur atoms in each formula, respectively.

$$\text{DBE} = 1 + \frac{2 \times C - H + N}{2} \quad (3)$$

$$\text{AI} = \frac{1 + C - 0.5 \times O - 0.5 \times H - S}{C - 0.5 \times O - N - S} \quad (4)$$

$$\text{NOSC} = 4 - \frac{4 \times C + H - 3 \times N - 2 \times O - 2 \times S}{C} \quad (5)$$

The magnitude-weighted average (M_w) was determined by dividing the peak magnitude (I_i) by the total sum of peak magnitudes for each sample.

$$M_w = \frac{\sum_i I_i \times M_i}{\sum_i I_i} \quad (6)$$

where *M* represents parameters *C*, *H*, *O*, *N*, O/C, H/C, DBE, and NOSC, respectively. The subscript *w* signifies a magnitude-weighted calculation, and I_i and M_i are the relative abundance and *M* value of peak *i,* respectively. The relative abundance is calculated as the abundance of the individual peak divided by the maximum abundance in each spectrum.

Supplementary Text 5 EDC Analysis Methods

An ABTS- and a chlorine-containing solution, and a pH buffer solution were prepared weekly and stored at room temperature, protected from light. The ABTS-containing solution was made by dissolving ABTS to a 1 mM final concentration in dilute sulfuric acid (7.5 mM, pH 2.0). The 1 mM chlorine-containing solution was prepared by diluting a concentrated hypochlorite stock solution in water. The stability of the chlorine and ABTS stock solutions was confirmed photometrically (data not shown). The pH buffer solution contained 100 mM of 2-Morpholinoethanesulphonic acid (MES) and resulted in a pH of 5.5 in the final reaction mixture of the EDC measurement[9].

First, the $ABTS^{\bullet+}$-reagent solution was prepared in bulk by adding 0.35 mL 1 mM chlorine solution per milliliter of ABTS solution (1 mM), which oxidized nominally 70% of the ABTS to $ABTS^{\bullet+}$; Second, 0.5 mL of $ABTS^{\bullet+}$ reagent solution was added to the buffered sample solution (3 mL of a DOM sample or DOM-free blank solution and 1.5 mL of pH buffer solution). A blank control was set to exclude the influence of the DOM's color on absorbance measurement (an equal volume of pure water was used instead of $ABTS^{\bullet+}$ solution). After a reaction time of 15 min, the solution was transferred to a disposable cuvette with a 10 mm pathlength (4.5 mL, poly(methyl methacrylate), Brand, Fisher). Finally, the resulting absorbance at 728 nm was measured on a spectrophotometer[9–11].

The EDC of a DOM sample was calculated based on the reductive loss of $ABTS^{\bullet+}$ in the reaction mixtures. $ABTS^{\bullet+}$ concentrations were quantified based on measured absorbances at 728 nm using the molar absorption coefficient ε(728 nm, pH 5.5) = 12580 M^{-1} cm^{-1} (Supplementary Fig. 10). To calculate the EDC, the residual $ABTS^{\bullet+}$ concentration in the reaction mixture containing DOM was compared to that of a reaction mixture containing only the DOM-free blank (i.e., ultrapure water) and normalized to the DOC concentration of the sample in the reaction mixture:

$$\mathrm{EDC} = \frac{A_{\mathrm{blank}} - A_{\mathrm{sample}}}{l \times \varepsilon_{\mathrm{ABTS}^{\cdot+}}} \times \frac{1}{C_{\mathrm{DOC}}} \quad (7)$$

where A_{blank} and A_{sample} are the resulting absorbance values (λ = 728 nm) of the reaction mixtures containing the DOM-free blank and the DOM sample, respectively, $\varepsilon_{\mathrm{ABTS}\cdot+}$ (M^{-1} cm^{-1}) is the molar absorption coefficient of $ABTS^{\bullet+}$, l (cm) is the optical pathlength, and C_{DOC} (mg$_C$ L^{-1}) is the DOC concentration in the final reaction mixture. For samples analyzed in replicates at increasing DOC concentrations, linear regression models were fitted to the measured residual $ABTS^{\bullet+}$ concentration versus the corresponding DOC concentration. The absolute value of the slopes of the fitted models represented the EDC values[9]:

$$c_{\mathrm{ABTS}\cdot+}^{\mathrm{sample}} = -\,\mathrm{EDC} \times C_{\mathrm{DOC}} + c_{\mathrm{ABTS}\cdot+}^{\mathrm{blank}} \quad (8)$$

where $c_{\mathrm{ABTS}\cdot+}^{\mathrm{sample}}$ and $c_{\mathrm{ABTS}\cdot+}^{\mathrm{blank}}$ are the $ABTS^{\bullet+}$ concentrations of the reaction mixtures containing the DOM-free blank and the DOM sample, respectively. EDC (mmol$_{e-}$ g$_C$$^{-1}$) is the electron-donating capacity, and C_{DOC} (mg$_C$ L^{-1}) is the DOC concentration in the final reaction mixture. Thus, the reported EDC is calculated from the slope of the linear relationship between $ABTS^{\bullet+}$ loss and DOC concentration, and the error bar was calculated as the standard error of the fitted slope.

Supplementary Text 6 SR-XAFS measurements

The data were recorded in fluorescence mode using a 32-element Ge solid-state detector, with energy calibration performed based on the absorption edge of pure Fe powder. The Athena and Artemis codes were employed for data extraction and profile fitting. For X-ray absorption near-edge structure (XANES) spectra, the experimental absorption coefficients as a function of energies $\mu(E)$ were processed using background subtraction and normalization procedures, resulting in "normalized absorption". The reference materials of Fe K-edge XANES included FeO, Fe_2O_3, and Fe foil. For extended X-ray absorption fine structure (EXAFS) analysis, Fourier-transformed (FT) data in R space were analyzed by applying a first-shell approximate or metallic Fe model for the Fe-O shell. Passive electron factors (S_0^2) were determined by fitting the experimental data of the Fe foil and fixing the coordination number (CN) of Fe-Fe for subsequent analysis of the measured samples. Key parameters, including corrections to the electronic photoelectron energy origin (E_0), CN, bond distance (R), and Debye-Waller factor (σ^2), which describe the electronic properties and local structural environment around the absorbing atoms, were allowed to vary during the fitting process.

Supplementary Text 7 DFT Calculations

The geometric optimizations were performed under periodic boundary conditions with a k-point sampling grid of 2 × 2 × 1 for the 1 × 4 supercell structure of the 110 facet of goethite, ensuring convergence of forces below 0.03 eV $Å^{-1}$. A vacuum layer of 25 Å was introduced in the z-direction to prevent interactions between periodic images. For the calculation of binding energies, both the isolated and complex systems were optimized separately to obtain the total energies, from which the binding energy was determined as:

$$E_{\text{binding}} = E_{\text{complex}} - (E_{\text{Hg(II)}} + E_{\text{functional moiety/goethite}}) \quad (9)$$

where E_{binding} is the total energy of the Hg-functional moiety or Hg-Cys/SA-goethite complex, and E_{complex}, $E_{\text{Hg(II)}}$, and $E_{\text{functional moiety/goethite}}$ are the total energies of isolated Hg and their respective counterparts. Spin-polarization was considered in all calculations due to the presence of unpaired electrons in the system. Convergence criteria for energy and forces were set to 10^{-6} eV and 0.03 eV $Å^{-1}$, respectively. The convergence of the total energy was monitored to ensure the reliability of the binding energy values obtained.

Supplementary Text 8 Source of Dataset for Spatial Analysis

The global topsoil Hg dataset used in this study was obtained from the published geospatial raster dataset of Guo et al.[12] This gridded dataset was developed from a global soil Hg database compiled from previously published measurements together with additional observations incorporated in the original study[13–38]. The full database included 18,907 soil Hg measurements across global land areas excluding Antarctica, and the 0–30 cm layer used here corresponded to the topsoil subset (n = 11,747). As described by Guo et al.[12], the dataset is an observation-driven global estimate generated at 0.1° × 0.1° spatial resolution and accompanied by grid-level uncertainty information; the original study further incorporated additional measurements from previously underrepresented tropical and arid regions to reduce extrapolation bias. Additional details and the original data are available from the original publication and its associated repository. In this study, we directly used the published 0–30 cm gridded layer for subsequent spatial analyses. The raster dataset of Guo et al. is publicly available on Figshare under a CC BY 4.0 license and was used here with source attribution. It is also provided in Supporting Data 1 of this study in the Figshare website (https://doi.org/10.6084/m9.figshare.29095691).

Supplementary References

1. Subdiaga, E. *et al.* Preferential sorption of tannins at aluminum oxide affects the electron exchange capacities of dissolved and sorbed humic acid fractions. *Environ. Sci. Technol.* **54**, 1837–1847 (2020).
2. Schnitzer, M. & Wright, J. R. Nitric acid oxidation of the organic matter of a Podzol. *Soil Sci. Soc. Am. J.* **24**, 273–276 (1960).
3. Priest, A. S. Molecular characterization of the soluble fraction of atmospheric particulate matter obtained by chemical oxidation with nitric acid. Dissertation, Old Dominion University (2010).
4. Perdue, E. M. Standard and reference samples of humic acids, fulvic acids, and natural organic matter from the Suwannee River, Georgia: thirty years of isolation and characterization. in *Functions of Natural Organic Matter in Changing Environment* (eds Xu, J., Wu, J. & He, Y.) 85–88 (Springer Netherlands, Dordrecht, 2013). https://doi.org/10.1007/978-94-007-5634-2_15.
5. Hughey, C. A., Hendrickson, C. L., Rodgers, R. P., Marshall, A. G. & Qian, K. Kendrick Mass Defect Spectrum: A compact visual analysis for ultrahigh-resolution broadband mass spectra. *Anal. Chem.* **73**, 4676–4681 (2001).
6. Koch, B. P. & Dittmar, T. From mass to structure: an aromaticity index for high-resolution mass data of natural organic matter. *Rapid Commun. Mass Spectrom.* **20**, 926–932 (2006).
7. Riedel, T., Biester, H. & Dittmar, T. Molecular fractionation of dissolved organic matter with metal salts. *Environ. Sci. Technol.* **46**, 4419–4426 (2012).
8. Hockaday, W. C., Purcell, J. M., Marshall, A. G., Baldock, J. A. & Hatcher, P. G. Electrospray and photoionization mass spectrometry for the characterization of organic matter in natural waters: a qualitative assessment. *Limnol. Oceanogr.: Methods* **7**, 81–95 (2009).
9. Walpen, N., Houska, J., Salhi, E., Sander, M. & von Gunten, U. Quantification of the electron donating capacity and UV absorbance of dissolved organic matter during ozonation of secondary wastewater effluent by an assay and an automated analyzer. *Water Res.* **185**, 116235 (2020).
10. Walpen, N., Getzinger, G. J., Schroth, M. H. & Sander, M. Electron-donating phenolic and electron-accepting quinone moieties in peat dissolved organic matter: quantities and redox transformations in the context of peat biogeochemistry. *Environ. Sci. Technol.* **52**, 5236–5245 (2018).
11. Zhu, S. *et al.* Chemically recalcitrant molecules are the primary components regulating the electron-donating capacity (EDC) of dissolved organic matter. *Water Res.* **287**, 124316 (2025).
12. Guo, W. *et al.* Warming-induced vegetation greening may aggravate soil mercury levels worldwide. *Environ. Sci. Technol.* **58**, 15078–15089 (2024).
13. Smith, D. B., Cannon, W. F., Woodruff, L. G., Solano, F., Kilburn, J. E. & Fey, D. L. Geochemical and mineralogical data for soils of the conterminous United States. U.S. Geological Survey, https://pubs.usgs.gov/ds/801/ (2013).
14. Campbell, L., Dixon, D. G. & Hecky, R. E. A review of mercury in Lake Victoria, East Africa: implications for human and ecosystem health. *J. Toxicol. Environ. Health B* **6**, 325–356 (2003).
15. Lado, L. R., Hengl, T. & Reuter, H. I. Heavy metals in European soils: a geostatistical analysis of the FOREGS geochemical database. *Geoderma* **148**, 189–199 (2008).
16. Xing, D. H., Liang, C. H., Wang, S. F. & Jia, Y. F. Concentration and speciation distribution of mercury in forest soils on northern slope of Changbai Mountain. *Chin. J. Ecol.* **30**, 106 (2011).
17. de Caritat, P. & Cooper, M. National geochemical survey of Australia: the geochemical atlas of Australia. Geoscience Australia, https://doi.org/10.11636/Record.2011.020 (2011).
18. Nartey, V. K., Klake, R. K., Doamekpor, L. K. & Sarpong-Kumankomah, S. Speciation of mercury in mine waste: case study of abandoned and active gold mine sites at the Bibiani–Anwiaso–Bekwai area of South Western Ghana. *Environ. Monit. Assess.* **184**, 7623–7634 (2012).
19. Tomiyasu, T., Kono, Y., Kodamatani, H., Hidayati, N. & Rahajoe, J. S. The distribution of mercury around the small-scale gold mining area along the Cikaniki river, Bogor, Indonesia. *Environ. Res.* **125**, 12–19 (2013).
20. Ma, M. *et al.* Mercury dynamics and mass balance in a subtropical forest, southwestern China. *Atmos. Chem. Phys.* **16**, 4529–4537 (2016).

21. Mungai, T. M. *et al.* Occurrences and toxicological risk assessment of eight heavy metals in agricultural soils from Kenya, Eastern Africa. *Environ. Sci. Pollut. Res.* **23**, 18533–18541 (2016).
22. Abeysinghe, K. S. *et al.* Total mercury and methylmercury concentrations over a gradient of contamination in earthworms living in rice paddy soil. *Environ. Toxicol. Chem.* **36**, 1202–1210 (2016).
23. Song, G. *et al.* Soil environmental element content dataset of Chinese ecosystem research network (1995–2011). China Sci. Data, https://doi.org/10.11922/csdata.170.2016.0102 (2017).
24. Gordeeva, O. N., Belogolova, G. A. & Pastukhov, M. V. Mercury speciation and mobility in soils of industrial areas in the Baikal region, Southern Siberia, Russia. *Environ. Earth Sci.* **76**, 558 (2017).
25. Schuster, P. F. *et al.* Permafrost stores a globally significant amount of mercury. *Geophys. Res. Lett.* **45**, 1463–1471 (2018).
26. Subhavana, K. L., Qureshi, A., Chakraborty, P. & Tiwari, A. K. Mercury and organochlorines in the terrestrial environment of Schirmacher Hills, Antarctica. *Bull. Environ. Contam. Toxicol.* **102**, 13–18 (2019).
27. Kang, H. *et al.* Characterization of mercury concentration from soils to needle and tree rings of Schrenk spruce (Picea schrenkiana) of the middle Tianshan Mountains, northwestern China. *Ecol. Indic.* **104**, 24–31 (2019).
28. Lim, A. G. *et al.* A revised pan-Arctic permafrost soil Hg pool based on Western Siberian peat Hg and carbon observations. *Biogeosciences* **17**, 3083–3097 (2020).
29. Wolswijk, G. *et al.* Mercury concentration data from Matang Mangrove Forest Reserve, Malaysia. *Data Br.* **29**, 105134 (2020).
30. Kapwata, T. *et al.* Spatial assessment of heavy metals contamination in household garden soils in rural Limpopo Province, South Africa. *Environ. Geochem. Health* **42**, 4181–4191 (2020).
31. Lei, D. *et al.* Soil-atmosphere exchange of gaseous elemental mercury in three subtropical forests with different substrate Hg concentrations. *Atmos. Environ.* **244**, 117869 (2021).
32. Alekseev, I. & Abakumov, E. Polycyclic aromatic hydrocarbons, mercury and arsenic content in soils of Larsemann Hills, Pravda Coast and Fulmar Island, Eastern Antarctica. *Bull. Environ. Contam. Toxicol.* **106**, 278–288 (2021).
33. Sahakyan, L., Tepanosyan, G., Maghakyan, N., Melkonyan, G. & Saghatelyan, A. Mercury contents and potential risk levels in soils and outdoor dust from kindergartens of the city of Vanadzor (Armenia). *Hum. Ecol. Risk Assess.: Int. J.* **27**, 1258–1275 (2021).
34. Velásquez Ramírez, M. G. *et al.* Mercury in soils impacted by alluvial gold mining in the Peruvian Amazon. *J. Environ. Manag.* **288**, 112364 (2021).
35. Ansah, E., Bak, J. L., Sørensen, P. & Darko, G. Modelling mercury concentration in Ghanaian soil. *Chemosphere* **307**, 135553 (2022).
36. Lima, F. R. D. *et al.* Geochemistry signatures of mercury in soils of the Amazon rainforest biome. *Environ. Res.* **215**, 114147 (2022).
37. Olson, C. I. *et al.* Mercury in soils of the conterminous United States: patterns and pools. *Environ. Res. Lett.* **17**, 074030 (2022).
38. Liu, Y.-R. *et al.* Multidimensional drivers of mercury distribution in global surface soils: insights from a global standardized field survey. *Environ. Sci. Technol.* **57**, 12442–12452 (2023).
