# Supplementary material for: An unexplored coupling process enhances dark Hg(II) reduction in mineral-Hg(II)-DOM ternary systems
Source: Nat Commun. 2026 Apr 24;17:6554. doi: 10.1038/s41467-026-72424-6 (PMC13381896; doi:10.1038/s41467-026-72424-6)
Supplement: Supplementary file 2 — Peer Review file [file 41467_2026_72424_MOESM2_ESM.pdf]

nature portfolio

Peer Review File

An unexplored coupling process enhances dark Hg(II) reduction in mineral-Hg(II)-DOM ternary systems

Corresponding Author: Professor Jiating Zhao

This file contains all reviewer reports in order by version, followed by all author rebuttals in order by version.

Version 0:

Reviewer comments:

Reviewer #1

(Remarks to the Author)
This manuscript describes that in the Goethite–Hg–DOM ternary system, goethite can facilitate the reduction of Hg(II) through mechanisms attributed to goethite transformation and DOM fractionation during their interactions. While this is an interesting finding, the promotional effect was observed to be highly dependent on the binding sequence: a significant enhancement in Hg(II) reduction occurred only when Hg(II) was pre-complexed with DOM prior to its introduction to goethite. Conversely, when DOM was pre-adsorbed onto the goethite surface, Hg(II) reduction was strongly inhibited. This sequence dependence raises concerns regarding the environmental relevance of the phenomenon, as iron oxides in natural systems are typically pre-associated with DOM. Furthermore, the proposed mechanistic link between Hg(II) reduction and shifts in DOM molecular composition is not robustly supported by the experimental data. For these reasons, I cannot recommend this manuscript for publication in Nature Communications.

Specific comments:
1. Figure 1 contains several presentational issues that hinder interpretation. First, the statistical analysis in Fig. 1c is unclear; although a significance level ($p < 0.05$) is noted, the specific pairwise comparisons are not identified. Furthermore, the origin of the 6 Hg(0) samples is not explained, and the abbreviations "LR-DHg" and "LR" are undefined. Second, the labels for experiments (ii) and (iii) in Figures 1d and 1e are inconsistent with their descriptions in the main text.

2. Line 113-117 Investigating the underlying mechanisms is needed to explain why hematite and magnetite do not facilitate Hg(II) reduction, and why hematite even inhibits Hg(II) reduction. Understanding these processes is critical for deciphering the role of mineral-Hg-DOM ternary system on the cycling of Hg.

3. Line 126-129 Please clarify the measured value of Hg(0) in the control group; is it 0.34 or 0.32 nM?

4. Line 128-131 In experiment (i), the production of Hg(0) gradually decreased after 4 h, a trend the authors attributed to reoxidation. However, the subsequent increase in Hg(0) at 24 hours contradicts this explanation, suggesting that reoxidation was not the primary cause.

5. Line 123-141 The significant influence of the addition sequence in the Goethite–Hg–DOM ternary system necessitates a more thorough investigation than the preliminary analysis of Hg redox states provided by the authors. In my view, a comprehensive analysis of the transformations of goethite, the fractionation of DOM, and their resultant electron-donating/accepting capacities in these different groups is essential. Such an inquiry is critically important because, in natural environments, these components do not interact in a fixed order.

6. Line 158-160 Liu et al reported that the Fe (oxyhydr)oxides increased the electron-accepting capacity of unbound DOM, and the authors claimed this process diminished the electron availability for Hg(II) reduction in Goethite–Hg–DOM ternary system. This attribution of Hg(II) reduction to solution-phase DOM seems to contradict the authors' later emphasis on interfacial interaction between DOM and goethite.

7. The term "initial DOM" in Figure 2 requires a more precise definition. Specifically, it should be clarified whether this refers

to a newly prepared solution, as the molecular composition of freshly dissolved organic matter (DOM) can evolve over time. Furthermore, the sample preparation protocol for FT-ICR-MS analysis, as described in the Methods section, lacks sufficient detail and should be elaborated to ensure reproducibility.

8. Section "DOM Molecular Fractionation in the Goethite-Hg-DOM Ternary System", identifies the specific functional groups lost from DOM in the solution phase following its interaction with goethite. The observed fractionation suggests that DOM undergoes a structural rearrangement upon adsorption, resulting in the formation of additional electron-donating groups. However, this mechanism remains speculative without direct evidence. To confirm it, the EDC of the goethite-adsorbed DOM must be measured directly.

9. Line 236-240 The emphasis on the increased EDC of the residual DOM in the solution promoting Hg(0) formation appears to contradict the authors' subsequent emphasis on the interfacial interactions between DOM and goethite.

Reviewer #2

(Remarks to the Author)
The authors studied the reduction of Hg in a goethite-Hg-DOM ternary system by using experimental and DFT calculations. This work is well-designed with full discussion. Nevertheless, the novelty or significance of this work in the field of mercury emission control is not very clear, which seems to be a routine work or an incremental work. Thus, it is not suitable for publication in Nat. Commun.
1.Introduction section, the hazard of mercury should be introduced. The latest laws or regulation for mercuy emission or discharge could be discussed as well.
2.The authors should add more texts to introduce or emphasize the novelty of this work, especially in new fundamental knowledge, new general methods, etc.
3.Line 251, the reason for the intensity reduction of the (110) and (020) diffraction peaks could be discussed more by citing pertinent literature.
4.Fig. 3 f and g, there are too many curves being compacted in the figures, which are too blurry and hard to follow. It is better to scale up them to make it clearer.
5.It is suggested to add a schematic or photo for the sorption experiments.

Reviewer #3

(Remarks to the Author)
Overall Summary:
This manuscript presents a comprehensive investigation into a previously overlooked pathway for dark reduction of Hg(II) in mineral-Hg-DOM ternary systems. The study is well-structured, employing a combination of batch experiments, advanced spectroscopic techniques (FT-ICR-MS, XAS), and DFT calculations. Overall, I think the findings are significant and could substantially advance our understanding of the global mercury cycle. However, several aspects regarding the mechanistic evidence, experimental design, and data interpretation require clarification before the manuscript can be accepted for publication.
Specific comments:
1. Lines 177-180: The terms "GHA-3", "GHA-6", "LR-3", "LR-6", "HR-3", and "HR-6" are used to denote specific samples for FT-ICR-MS analysis but are not explicitly defined in the main text or figure caption for Fig. 1f. Please clearly state what these suffixes (e.g., -3, -6) represent.
2. Lines 128-130: The decline in Hg(0) yield after 4 hours in sequential experiment (i) is attributed to "DOM-mediated reoxidation." While this is a hypothesis, no direct evidence is provided to confirm that the decrease in Hg(0) is due to its re-oxidation back to Hg(II) (e.g., by tracking the re-appearance of aqueous Hg(II) or identifying the oxidized Hg species). Please provide supporting data for this claim or temper the language to present it as a potential explanation among others.
3. Lines 265-275: The XAS data (XANES and EXAFS) suggest a partial reduction of Fe(III) in goethite. It appears that the formation of Fe(II), which is a critical product of the proposed electron transfer from DOM, is not directly confirmed. To solidify this key part of the mechanism, it would be highly beneficial to provide direct evidence for Fe(II) presence on the solid phase after reaction or by marking the characteristic pre-edge features of Fe(II) and Fe(III) in the XANES spectra (Fig. 3f).
4. Lines 86-91 & 427-429: The study focuses on goethite and briefly compares it with hematite and magnetite. Given the diversity of iron (oxyhydr)oxides in the environment (e.g., ferrihydrite, lepidocrocite), it would be nice to discuss the environmental implications where other minerals dominate.
5. Lines 86-87: The use of a single type of humic acid (HA) as a DOM proxy may limit the scope of the findings. Natural DOM is highly heterogeneous. Please acknowledge this limitation in the discussion and suggest that future work should validate these processes with DOM from various environmental sources.

6. Lines 289-291: The use of cysteine and salicylic acid as model compounds for DOM is a necessary simplification. However, the discussion should more explicitly state the limitations of this approach, as it cannot capture the complexity, cooperative effects, and steric constraints of a macromolecular DOM matrix. Please elaborate on how these simplifications might affect the interpretation of the binding energies and reduction pathways.
7. Lines 357-369 & 471-484: The global scaling exercise is a valuable addition. However, the parameterization introduces significant uncertainty. The binary classification of the residual Hg fraction (f) solely based on a total Hg concentration threshold of 0.25 μM is a major simplification, as Hg speciation is influenced by numerous local factors (soil type, organic matter, redox conditions). The discussion should clearly acknowledge this aspect.
8. Lines 196-197 & Fig. 2: The non-detection of carbohydrate-like molecules (group 3) is noted. This might be an artifact of the sample preparation (PPL solid-phase extraction) and ionization technique (negative ESI). Please add a sentence in the

methods or figure caption to clarify that the FT-ICR-MS results represent the “SPE-amenable, ESI-ionizable” fraction of DOM, not its total composition.
9. Fig.6a and b: Please check the font of the axes.

Version 1:

Reviewer comments:

Reviewer #4

(Remarks to the Author)
This study identifies a missing dark mercury reduction pathway operating in mineral-Hg-DOM ternary systems and fills an important gap in our understanding of the global mercury cycle. The authors present a clear and rigorous body of work that combines laboratory experiments, FT-ICR-MS, synchrotron-based XAS, DFT calculations, and global-scale modeling. The study is well organized, the figures are clear and informative, and the proposed coupling between goethite transformation and DOM fractionation is strongly supported by the data.
I have personally been involved in research on dark mercury reduction. In previous efforts to quantify microbial-mediated Hg reduction under dark conditions, we repeatedly encountered mass-balance discrepancies in which experimental results failed to match with field observations. The field has explored microbial pathways and gene-related processes, such as the possible roles of merA, but their contribution under dark conditions appears limited and often inconsistent. In both aquatic and soil systems, these processes have not provided a clear explanation for observed mercury reduction. This study offers a simple and convincing abiotic explanation. It shows that mercury can be reduced through coupled reactions between iron mineral transformation and DOM fractionation at the solid-water interface. This mechanism can operate in the absence of light and does not rely on specific biological conditions. It therefore provides a realistic explanation for mercury reduction in environments such as deep-sea sediments and subsurface soils, where iron minerals and DOM are common.
The novelty and broad environmental relevance of this mechanism are significant. Importantly, it provides a robust and environmentally realistic explanation that is applicable across both soil and aquatic systems, substantially advancing our predictive understanding of mercury cycling under dark conditions. The comments raised by the previous reviewers were generally constructive and appropriate for a study of this scope. The authors have responded well and have revised the manuscript accordingly, resulting in a clearer, more coherent, and more persuasive presentation of the work. The remaining issues are minor and primarily relate to clarity and framing rather than scientific validity. I therefore recommend acceptance of this manuscript after minor revision.

Specific Comments:
1. Fig. 5 has a strong summary of this work. Adding a short guiding sentence in the text when first referring to the conceptual figure would improve readability.
2. Please clarify whether surface Fe(II) is expected to directly reduce Hg(II) or mainly support DOM-mediated electron transfer.
3. The experiments were conducted at pH 5.5. A brief note on how higher or lower pH might affect ternary complex stability would help extend the environmental relevance.
4. A short clarification on whether light exclusion was strictly maintained throughout the experiments would improve transparency.
5. The mechanism you’ve discovered has clear implications beyond topsoil. A short sentence highlighting why this mechanism matters beyond soils, for example in sediments or subsurface systems, would strengthen the opening.
6. The study convincingly demonstrates that mineral transformation and DOM fractionation are coupled rather than independent processes. The manuscript already provides a clear explanation of this coupling, which effectively prevents readers from interpreting these effects as parallel but unrelated events.
7. The comparison between different iron minerals is informative. A short clarification on whether mineral reactivity or surface chemistry is the dominant control would strengthen the mechanistic interpretation.

Reviewer #5

(Remarks to the Author)
This manuscript investigates Hg redox transformations in a Hg–DOM–goethite ternary system and provides interesting insights into the coupled interactions among Hg, dissolved organic matter (DOM), and Fe (oxyhydr)oxides. In particular, the study suggests that adsorption of DOM onto goethite can modify the redox characteristics of the remaining aqueous DOM, which may subsequently influence Hg redox transformations. The authors also propose that Hg–goethite–DOM ternary complexes may be more susceptible to Hg(II) reduction compared with Hg–DOM complexes alone. These observations are potentially important because they highlight how mineral–DOM interactions can indirectly regulate Hg speciation and redox behavior in natural environments.

However, several conceptual interpretations, experimental design choices, and descriptions of Hg speciation require further clarification and discussion. In particular, the novelty of the proposed “missing reduction pathway”, the role of thiol-controlled Hg speciation, and the interpretation of Hg reduction and reoxidation processes in the experimental system need to be more carefully evaluated.

Major comments

1. The title “A missing reduction pathway of Hg by coupled reactions in mineral–Hg–DOM ternary systems” is somewhat misleading. I do not think the process described here represents a “missing” reduction pathway. Surface-mediated reduction of heavy metals on Fe (oxyhydr)oxides, both in the absence and presence of DOM, has been widely studied in environmental geochemistry, for example, As, Cr, and Cd on Fe minerals have been extensively reported. In many of these systems, Fe(II) plays a key catalytic role by promoting mineral transformation of Fe (oxyhydr)oxides and facilitating electron transfer processes that lead to metal reduction.

Therefore, the mechanism discussed in this manuscript is not fundamentally new. The apparent novelty may arise from the fact that similar processes have been much less studied for Hg compared with other heavy metals. Nevertheless, the underlying mechanisms are likely similar.

By the way, one important reason why fewer studies on Hg compared with other metals is the typically low environmental concentration of Hg. For many metals, their speciation in nature is largely controlled by surface reactions with Fe (oxyhydr)oxides, including adsorption or surface precipitation. In contrast, Hg speciation is more strongly controlled by complexation with DOM. Because Hg concentrations are usually extremely low, even relatively small amounts of DOM can dominate Hg speciation. Hg has a very strong affinity for thiol groups in DOM. Under environmentally relevant Hg concentrations, Hg typically forms Hg(DOM-RS)2 complexes with thiol groups. At higher Hg concentrations where thiol groups become saturated, Hg may also bind to oxygen-containing functional groups (such as carboxyl groups). In sulfidic environments, Hg forms HgS phases. Only under very certain conditions does Hg significantly interact with Fe (oxyhydr)oxide surfaces.

2. The authors used goethite as the Fe mineral in their experiments. However, in natural environments different Fe minerals dominate under different redox conditions. Under oxic surface conditions, ferrihydrite, goethite, and hematite are commonly present. Among which, ferrihydrite is much more abundant than goethite. Ferrihydrite is poorly crystalline and typically exhibits much higher adsorption capacity and greater reactivity. It also undergoes Fe(II)-catalyzed mineral transformations more readily, making it highly relevant for redox reactions involving electron transfer. Under more reducing conditions, Fe sulfides such as FeS and FeS_2 are typically dominant, while intermediate redox conditions often contain magnetite or green rust phases. These minerals can be more environmentally relevant for dark reduction processes. In contrast, Hg(II) reduction under oxic surface conditions is often associated with photochemical processes. Given these considerations, it would be useful for the authors to justify the choice of goethite. It is also worth discussing why ferrihydrite or other more reactive Fe minerals were not selected, as they may play a more important role in environmental Hg transformations.

3. The Hg–DOM–goethite ternary system is very complex. In the simpler Hg–DOM system, multiple processes occur simultaneously. Hg can complex with DOM through both thiol (RSH) and oxygen-containing functional groups (ROH), depending on the Hg/DOM ratio. These complexes have very different thermodynamic stabilities and reactivity especially on Hg(II) reduction. DOM can also act as both electron donors and electron acceptors, can reduce Hg(II) to Hg(0) but also in parallel, oxidize Hg(0)

In the Hg–goethite system, Hg(II) may form surface complexes with goethite. When Fe(II) is present, Fe(II) can catalyze mineral transformation of goethite into mixed Fe(II)–Fe(III) (oxyhydr)oxide phases, which may enhance electron transfer and Hg(II) reduction. Bridging structures involving metal–DOM–Fe (oxyhydr)oxides have been widely reported for other metals. Therefore, when studying Hg–DOM–Fe mineral systems, it is important to first understand the simpler subsystems and how DOM and Fe minerals evolve during the reaction. For example, Fe mineral transformations should be monitored by tracking both aqueous and adsorbed Fe(II), since Fe(II) strongly influences mineral transformation pathways. The relative abundance of Fe(II) and mixed Fe(II)–Fe(III) phases can significantly influence the redox properties of Fe minerals. It said in the Abstract “resulting in newly formed electron shuttles, electron donors, and iron redox couples” but the electron shuttles, Fe(II) concentration and Fe-mineral transformation, etc were not displayed.

In addition, the production of Hg0 observed in the experiments represents the net result of reduction and oxidation processes. Therefore, the potential reoxidation of Hg0 should also be considered when interpreting the results. For example, the observed Hg0 production may underestimate the actual extent of Hg(II) reduction, because a portion of the generated Hg0 could have been reoxidized rather than indicating that Hg(II) (in the presence of goethite) was less stable.
Specific comments
Line 108–109: The authors define two Hg/DOC molar ratios. However, because Hg speciation is largely controlled by thiol groups, the Hg/thiol ratio would be a more relevant parameter, therefore should be displayed also.
Line 120: The reduction of Hg(II) by DOM is regulated by both the reducing capacity of DOM (electron-donating functional groups) and the speciation of Hg(II).

Figure 1: In panels b and c, does Ce-DOC represent the DOC concentration in the supernatant after the 24-hour reaction shown in Fig. 1a? If so, the authors should demonstrate that 24 h is sufficient to reach equilibrium; otherwise these concentrations cannot be interpreted as equilibrium values.

Line 173: “A third reactant was introduced 2 h after their initial reactions.” Why was 2 h selected? A longer equilibration time (e.g., 24 h) might be more appropriate to ensure that the initial reactions have reached equilibrium.

Line 184: “slows both Hg reduction and re-oxidation, allowing Hg(0) to accumulate over time”. This interpretation may be incorrect. The system is closed, and the Hg(II) ⇌ Hg(0) transformation is a reversible reaction. The reduction of Hg(II) depends on electron donors and Hg(II) speciation (e.g., Hg-thiol vs Hg-carboxyl). Meanwhile, DOM may also contain

electron-accepting groups capable of oxidizing Hg(0). The oxidation of Hg(0) depends on both the concentration of electron acceptors and the concentration of dissolved Hg(0). Dissolved Hg(0) is governed by Henry's law equilibrium with Hg(0) in the headspace. When Hg(0) accumulates in the headspace of the reaction vial, the concentration of dissolved Hg(0) may increase, potentially promoting re-oxidation to Hg(II).

Line 195–197: "a characteristic energy shift toward lower energy indicating more reduced Hg species". If this is correct, it would imply the presence of Hg(0) in the EXAFS samples. Therefore, the authors should provide detailed information on sample preparation and preservation for XAS measurements. For example: Were the samples frozen immediately after collection? Were the XAS measurements conducted at cryogenic temperatures (e.g., liquid nitrogen or liquid helium)? Furthermore, the presence of Hg(0) should be confirmed using XANES analysis or EXAFS fitting.

Line 211–212: "the mineral-Hg-DOM ternary complex formation controls the fate of Hg in natural environments". This statement seems inconsistent with the earlier claim that thiol groups in DOM are not saturated and that Hg forms Hg(RS)2 complexes. Because Hg has extremely strong affinity for thiol groups, Hg(RS)2 complexes should dominate over Hg–mineral interactions. The stability constants differ by many orders of magnitude. Therefore, the estimation of thiol concentrations in DOM should be carefully evaluated (and the estimation of thiol concentration should be detailed described).

Line 230–233: The statement regarding Hg(OR)2 complexes hindering ligand exchange is likely incorrect. Hg(RS)2 complexes are the most stable species and would be expected to inhibit ligand exchange. In contrast, Hg(OR)2 complexes compete with mineral surface binding. If thiol groups are not saturated, Hg(DOM-RS)2 complexes should dominate. Only when thiol groups become saturated would excess Hg bind to oxygen-containing groups in DOM or mineral surfaces. A thermodynamic speciation model should be used to estimate the relative abundance of Hg(DOM-RS)2, Hg–DOM–mineral complexes, and Hg–mineral complexes.

Line 375–379: The calculation results are interesting. However, more experimental evidence is required to support the conclusion that mineral-Hg-DOM complexes dominate over Hg(Cys/SA)2-like complexes (in lab experiment and natural environments).

Line 382–384: The statement that bidentate structures such as Hg(OR)2, RSHgOR, and Hg(SR)2 are thermodynamically less stable requires clarification. In addition, the experimental results show enhanced Hg(0) production in the presence of goethite. If Hg(OR)2, RSHgOR, and Hg(SR)2 are less stable than Hg–goethite–DOM ternary complex, this interpretation may appear contradictory.

Line 387–391: The discussion should include a more detailed consideration of Hg^0 reoxidation, since the observed Hg^0 production represents a net result of reduction and oxidation processes.

Line 482–483: Batch experiments were conducted to examine the sorption of DOM (0.83–8.75 mM) and Hg(II) (Hg(NO3)2, 0.32–13.34 μM) on goethite (2 g/L)." This description is unclear. Does it refer to:
1. DOM adsorption on goethite
2. Hg adsorption on goethite
3. adsorption of the DOM–Hg mixture on goethite?
The concentrations listed here also differ from those described in the following paragraph.

Line 489–493: More information should be provided regarding the concentration of thiol groups in the PPHA. Table S1 only reports total sulfur concentrations, which do not necessarily represent thiol groups. Without quantitative information on thiol concentration, it is premature to conclude that Hg(II) is primarily complexed with thiol groups.

Adsorption experiments: more experimental details should be provided, particularly for systems containing DOM. The adsorption behavior of DOM is strongly influenced by pH, ionic strength, and other solution parameters.

Line 494: Why were Hg(II) and DOM equilibrated for only 8 h before adding goethite? Hg–DOM complexation is often kinetically controlled, and many studies use 24 h equilibration.
The procedure described in this paragraph is not entirely clear. For example, was the sequence: DOM + Hg(II) → 8 h equilibration → addition of goethite? Was purging performed before or after goethite addition?
Line 506: low Hg(II) concentrations instead of low Hg(0) concentrations?

Line 546–547: The ratio used in the model system (1 mM DOM with 5 mM Hg) is extremely different from the experimental conditions.

Line 555–556: SA and cysteine cannot represent natural DOM. Although these molecules contain carboxyl and thiol functional groups, natural DOM has complex three-dimensional structures and heterogeneous functional group distributions that cannot be mimicked by simple molecules. Is it possible to use big molecules, for instance, proteins?

Open Access This Peer Review File is licensed under a Creative Commons Attribution 4.0 International License, which permits use, sharing, adaptation, distribution and reproduction in any medium or format, as long as you give appropriate credit to the original author(s) and the source, provide a link to the Creative Commons license, and indicate if changes were made.
In cases where reviewers are anonymous, credit should be given to 'Anonymous Referee' and the source.
The images or other third party material in this Peer Review File are included in the article's Creative Commons license, unless indicated otherwise in a credit line to the material. If material is not included in the article's Creative Commons license and your intended use is not permitted by statutory regulation or exceeds the permitted use, you will need to obtain permission directly from the copyright holder.
To view a copy of this license, visit https://creativecommons.org/licenses/by/4.0/

Response to the reviewers

On behalf of all co-authors, I would like to thank the reviewers for dedicating time to provide these valuable comments and criticisms. The reviewers raised issues that have helped greatly to improve our manuscript. According to these comments, we have completed a major revision with substantial efforts. For clarity, we present reviewer comments in *blue italics*, our response to comments in normal text, and a description of the changes to the manuscript in underlined text, extended data and figures in blue underlined text. The revisions in the manuscript and supplementary materials are highlighted in blue.

Reviewer #1 (Remarks to the Author)

This manuscript describes that in the Goethite–Hg–DOM ternary system, goethite can facilitate the reduction of Hg(II) through mechanisms attributed to goethite transformation and DOM fractionation during their interactions. While this is an interesting finding, the promotional effect was observed to be highly dependent on the binding sequence: a significant enhancement in Hg(II) reduction occurred only when Hg(II) was pre-complexed with DOM prior to its introduction to goethite. Conversely, when DOM was pre-adsorbed onto the goethite surface, Hg(II) reduction was strongly inhibited. This sequence dependence raises concerns regarding the environmental relevance of the phenomenon, as iron oxides in natural systems are typically pre-associated with DOM. Furthermore, the proposed mechanistic link between Hg(II) reduction and shifts in DOM molecular composition is not robustly supported by the experimental data.

Response: Many thanks for the constructive comments regarding the sequence dependence and environmental relevance of Hg(II) reduction in the goethite-Hg-DOM system. Yes, we acknowledge that DOM in many ecosystems is typically pre-associated with iron and other metal oxides, while free DOM and iron oxides (e.g., goethite) are also widely present and constitute significant forms in natural environments. For instance, in porewater, groundwater, and wetland systems, a substantial fraction of DOM remains mobile, and Hg(II) predominantly occurs as DOM-associated species even at low Hg/DOM ratios. This leads to an initial homogeneous complexation between Hg(II) and DOM, followed by heterogeneous interactions of DOM-Hg species with Fe (oxyhydr)oxide surfaces. In these scenarios, Hg(II) tends to associate first with free DOM rather than with mineral-adsorbed

DOM for the following reasons:

1) Complexation between Hg(II) and free DOM is a homogeneous reaction that proceeds rapidly and is often close to diffusion-controlled rates. By contrast, reactions involving mineral-bound DOM occur through heterogeneous pathways that depend on transport of Hg(II) to the surface and are therefore limited by boundary-layer and surface-accessibility constraints (Weber & Morris, 1963; Willkins, 1991).
2) Thermodynamic measurements further show that free DOM binds Hg(II) very strongly, with conditional stability constants on the order of log K ≈ 21–23 under environmentally relevant Hg/DOM ratios, meaning that dissolved Hg is dominated by Hg-DOM complexes in most natural waters (Benoit et al., 2001; Haitzer et al., 2002; Ravichandran, 2004).
3) In addition, DOM that has been immobilized on mineral surfaces does not retain the same reactivity as dissolved DOM: spectroscopic studies indicate that adsorption reduces the accessibility of functional groups that could participate in metal binding (Oren and Chefetz, 2012; Avneri-Katz et al., 2017).

Taken together, these factors make it more likely that Hg(II) is captured by free DOM before it interacts with DOM constrained on mineral surfaces.

Therefore, we clarify that the sequence in which Hg(II) is pre-complexed with DOM before interaction with goethite represents a common and environmentally realistic pathway in the environment, particularly DOM-rich environments such as wetlands, peatlands, and anoxic sediments. DOM-Hg complexation proceeds rapidly in homogeneous solution and is thermodynamically dominant, with dissolved Hg occurring predominantly as DOM-associated species under environmentally relevant Hg/DOM ratios. In contrast, reactions involving mineral-bound DOM are heterogeneous and constrained by surface accessibility, and adsorption of DOM onto mineral surfaces substantially reduces the availability of functional groups involved in metal binding and electron transfer.

Moreover, although Fe oxides are often pre-associated with organic matter in soils, a significant fraction of DOM remains mobile in porewaters and surface waters, where Hg(II) is transported primarily as DOM-bound species. Our data show that even after Hg-DOM equilibration, free DOM, iron(oxyhydr)oxides, and DOM-Hg complexes coexist in solution. In this system, goethite enables dynamic competition for surface sites and distinct interfacial reaction pathways. The inhibition of

Hg(II) reduction observed when DOM is pre-adsorbed onto goethite indicates that surface immobilization of DOM limits the availability of redox-active functional groups and constrains interfacial electron transfer. Conversely, the enhanced Hg(II) reduction observed in the Hg→DOM→goethite sequence highlights the critical role of DOM mobility and molecular accessibility. We have revised the manuscript to clarify that the observed sequence dependence provides mechanistic insight into coupled mineral-Hg-DOM interactions by distinguishing solution-phase processes from mineral-associated interfacial reactions. (Line 52–59 & 88–94)

To further strengthen the mechanistic link between Hg(II) reduction and DOM molecular properties, we have conducted additional electron-donating capacity (EDC) measurements, which provide independent support for our interpretation. These results are presented and discussed in detail below in response to the reviewer's subsequent comments. (Line 296–304)

Reference

Avneri-Katz, S. et al. Adsorptive fractionation of dissolved organic matter (DOM) by mineral soil: Macroscale approach and molecular insight. Organic Geochemistry 103, 113–124 (2017).

Benoit, J. M., Mason, R. P., Gilmour, C. C. & Aiken, G. R. Constants for mercury binding by dissolved organic matter isolates from the Florida Everglades. Geochimica et Cosmochimica Acta 65, 4445–4451 (2001).

Haitzer, M., Aiken, G. R. & Ryan, J. N. Binding of Mercury(II) to Dissolved Organic Matter: The Role of the Mercury-to-DOM Concentration Ratio. Environ. Sci. Technol. 36, 3564–3570 (2002).

Oren, A. & Chefetz, B. Sorptive and Desorptive Fractionation of Dissolved Organic Matter by Mineral Soil Matrices. Journal of Environmental Quality 41, 526–533 (2012).

Ravichandran, M. Interactions between mercury and dissolved organic matter—a review. Chemosphere 55, 319–331 (2004).

Weber, W. J. & Morris, J. C. Kinetics of Adsorption on Carbon from Solution. J. Sanit. Engrg. Div. 89, 31–59 (1963).

Wilkins, R. G. Kinetics and Mechanism of Reactions of Transition Metal Complexes. (Wiley, 1991). doi:10.1002/3527600825.

1. Figure 1 contains several presentational issues that hinder interpretation. First, the statistical analysis in Fig. 1c is unclear; although a significance level ($p < 0.05$) is noted, the specific pairwise comparisons are not identified. Furthermore, the origin of the 6 Hg(0) samples is not explained, and the abbreviations "LR-DHg" and "LR" are undefined. Second, the labels for experiments (ii) and (iii) in Figures 1d and 1e are inconsistent with their descriptions in the main text.

Response: Thank you for pointing out the presentation issues in Fig. 1. The statistical analysis in Fig. 1c (Modified Fig. 1d) was designed to assess whether the introduction of goethite alters Hg(0) production under otherwise identical conditions. A paired *t-test* was therefore applied to directly compare experiments conducted with and without goethite, which is appropriate and sufficient for isolating the effect of goethite. This comparison has now been explicitly clarified in the revised figure caption. The origin of the six Hg(0) samples and the definitions of "LR-DHg" and "LR" have now been clearly defined in the main text and the figure legend. In addition, the labels for experiments (ii) and (iii) in Fig. 1d and 1e (Modified Fig. 1e and 1f) have also been checked and corrected accordingly in the revised figures to ensure consistency throughout the manuscript.

(Line 115–117)

Modified Fig. 1:

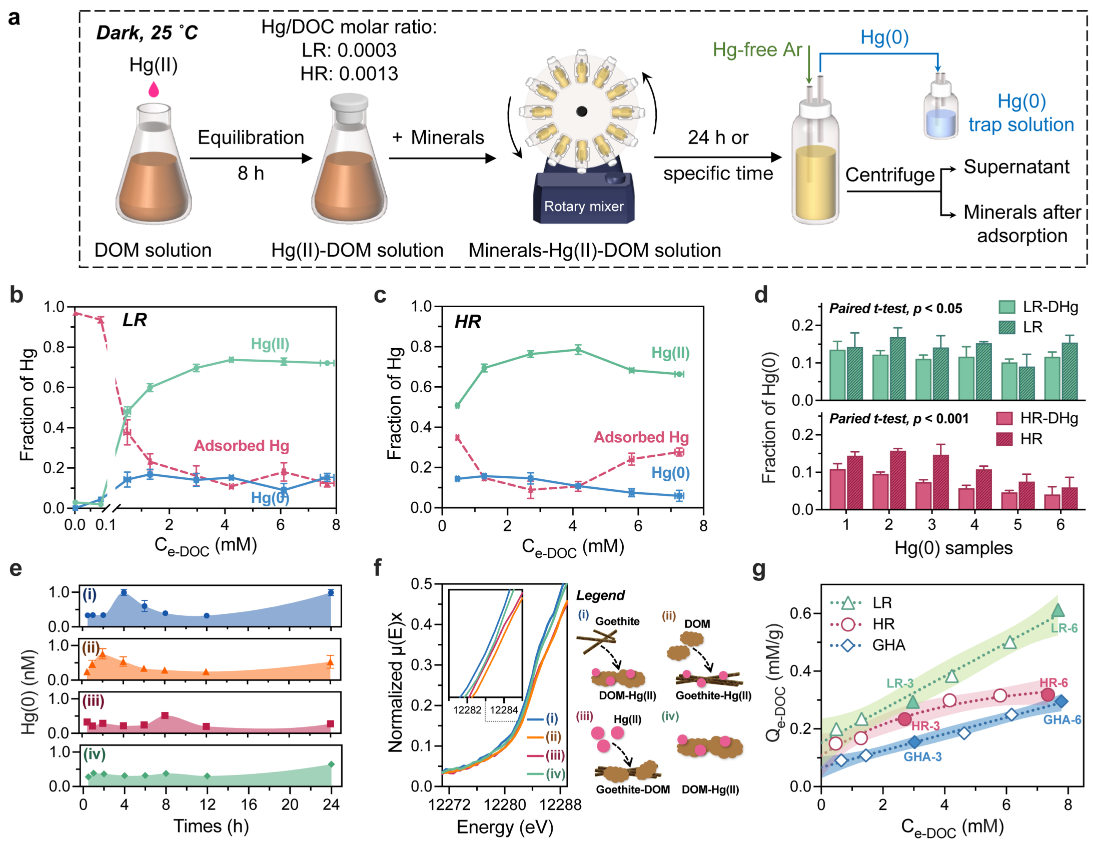

2. Line 113-117 Investigating the underlying mechanisms is needed to explain why hematite and magnetite do not facilitate Hg(II) reduction, and why hematite even inhibits Hg(II) reduction. Understanding these processes is critical for deciphering the role of mineral-Hg-DOM ternary system on the cycling of Hg.

Response: Thanks for the suggestion. Indeed, elucidating why the three minerals exert such distinct effects on Hg(II) reduction is a key component of our next-stage research. To begin addressing this point within the scope of the current study, we conducted additional kinetic experiments with all three minerals and measured the EDC and DOC of residual DOM in the supernatants at multiple reaction time points (Extended Data Supplementary Fig. S4b and S4c). The results show that Hg enhanced DOM adsorption onto all three minerals. However, in the hematite-Hg-DOM system, no measurable EDC was detected at pH 5.5. Instead, repeated measurements consistently showed an increase in $ABTS^{\bullet+}$ absorbance, indicating that the solution behaved predominantly as an electron acceptor rather than an electron donor. This behavior explains the much lower production of Hg(0) in the hematite system, as any transiently formed Hg(0) would be readily reoxidized. This interpretation is consistent with previous studies showing that hematite strongly and effectively scavenges carboxyl, phenolic, and other redox-active functional groups at pH 5.5 (Fu and Quan, 2006), thereby depleting solution-phase electron-donating moieties more efficiently than goethite.

In contrast, magnetite exhibited a weaker affinity for DOM, resulting in greater DOM retention in solution and correspondingly higher EDC values. However, supplementary Fe^{2+} measurements revealed that Fe(II) release from magnetite was suppressed in the presence of Hg (Extended Data Supplementary Fig. S4d and S4e), consistent with DOM adsorption limiting magnetite dissolution and restricting access to structural Fe(II) sites. This decrease indicates that the presence of Hg(II) suppresses mineral-associated redox processes, likely by limiting access to structural Fe(II) sites. Consequently, although more DOM remains in solution, the effective electron availability relevant to Hg(II) reduction is not enhanced in the ternary system. This interpretation aligns with the observation that Hg(0) production in the magnetite-Hg-DOM system is comparable to that in the DOM-Hg binary system.

Together, these additional measurements provide mechanistic support for the distinct Hg(II) reduction behaviors observed among hematite, magnetite, and goethite. We have incorporated these results and their interpretation into the revised Results and Discussion sections to address your concern regarding mineral-specific controls on Hg(II) reduction. (Line 147–168)

Extended Data Supplementary Fig. S4:

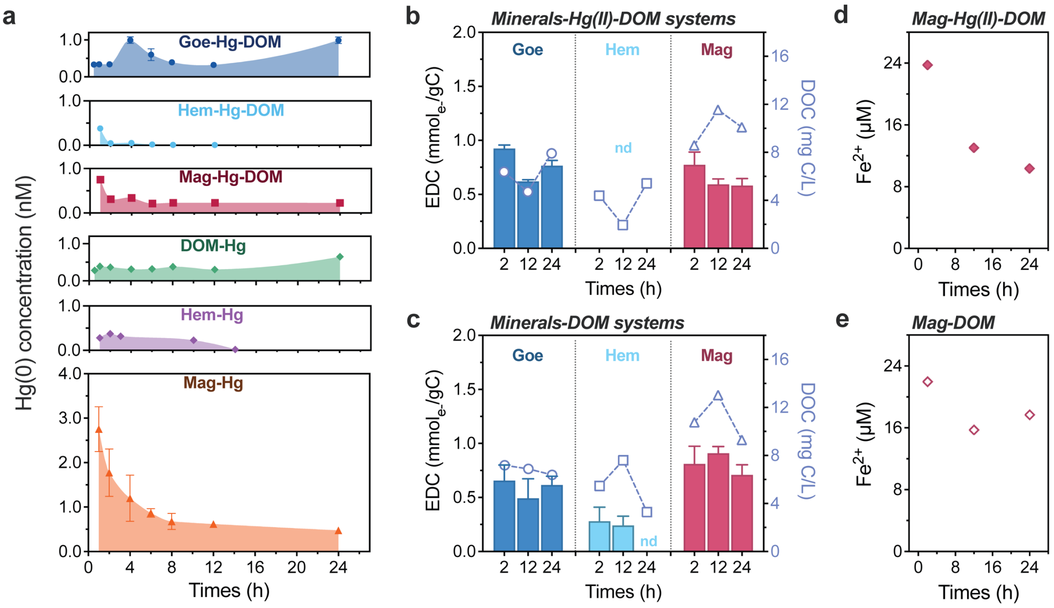

Reference

Fu, H. & Quan, X. Complexes of fulvic acid on the surface of hematite, goethite, and akaganeite: FTIR observation. Chemosphere 63, 403–410 (2006).

3. Line 126-129 Please clarify the measured value of Hg(0) in the control group; is it 0.34 or 0.32 nM?

Response: We thank the reviewer for pointing out this ambiguity. The value of 0.34 nM represents the average Hg(0) production over the first 12 h of reaction, whereas 0.32 nM corresponds to the Hg(0) concentration measured at the specific 4 h time point. We have revised the text to clearly distinguish between time-averaged production and point-in-time concentration to avoid confusion. (Line 115)

4. Line 128-131 In experiment (i), the production of Hg(0) gradually decreased after 4 h, a trend the authors attributed to reoxidation. However, the subsequent increase in Hg(0) at 24 hours contradicts this explanation, suggesting that reoxidation was not the primary cause.

Response: Thanks for the question. Yes, we repeated this experiment several times and confirmed the phenomenon as a complex reaction process. In our kinetic setup, Hg(II), DOM, and minerals reacted for 24 h before Hg(0) was purged, rather than under continuous aeration. Thus, the measured

Hg(0) reflects the net effect of simultaneous Hg(II) reduction and Hg(0) re-oxidation, along with ongoing structural evolution of Hg-DOM complexes. Given the complexity of these coupled processes, we may provide a plausible interpretation based on the available data and published research. According to Jiang et al. (2015), in the DOM-Hg system, the late-stage Hg(0) increase can be explained by time-dependent structural rearrangement of Hg-DOM complexes. Specifically, Hg(II) initially binds to O/N-rich functional groups, leading to relatively rapid reduction but also rapid Hg(0) re-oxidation. As these O/N sites are progressively consumed, Hg(II) becomes increasingly coordinated by S-containing groups, which slows reduction yet also diminishes Hg(0) re-oxidation, allowing Hg(0) to accumulate in the later reaction interval. In the ternary systems, the behavior is further influenced by DOM-mineral interactions. We performed additional measurements of EDC and DOC at 2, 12, and 24 h, the results show that all addition sequences exhibit an increase in solution-phase EDC and DOC at 24 h (Extended Data Supplementary Fig. S6a). This pattern suggests that a portion of DOM returned to the aqueous phase at the later stage of reaction, which is consistent with previous findings that non-carboxyl-rich DOM fractions readily desorb from mineral surfaces (Oren and Chefetz, 2012). Such late-stage DOM release increases the pool of electron-donating functional groups in solution and provides a plausible basis for the renewed Hg(0) formation.

Given the inherent complexity of the system, to be more rigorous, we have added the related discussion representing the reasonable interpretation grounded in our experimental observations and the literature. The relevant EDC/DOC data and explanatory text have been incorporated into the Results and Discussion of the revised manuscript. (Line 179–189)

Extended Data Supplementary Fig. S6:

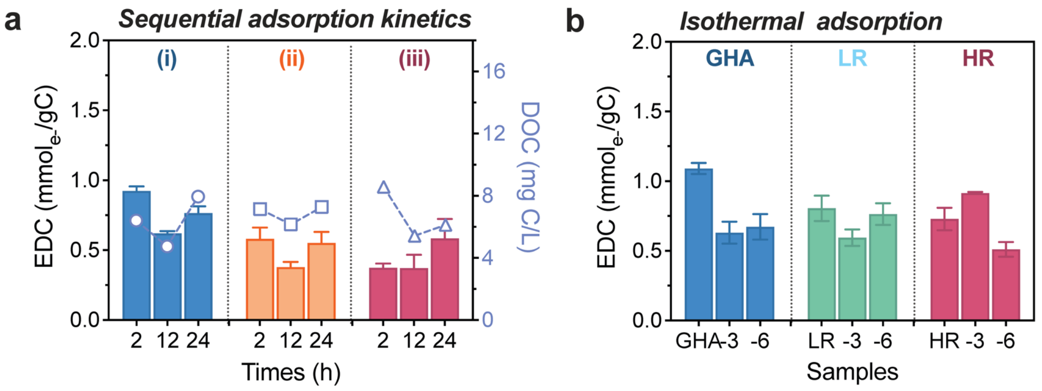

Reference

Jiang, T. et al. Modeling of the structure-specific kinetics of abiotic, dark reduction of Hg(II) complexed by O/N and S functional groups in humic acids while accounting for time-dependent structural rearrangement. Geochim. Cosmochim. Acta 154, 151–167 (2015).

Oren, A. & Chefetz, B. Sorptive and Desorptive Fractionation of Dissolved Organic Matter by Mineral Soil Matrices. Journal of Environmental Quality 41, 526–533 (2012).

5. Line 123-141 The significant influence of the addition sequence in the Goethite–Hg–DOM ternary system necessitates a more thorough investigation than the preliminary analysis of Hg redox states provided by the authors. In my view, a comprehensive analysis of the transformations of goethite, the fractionation of DOM, and their resultant electron-donating/accepting capacities in these different groups is essential. Such an inquiry is critically important because, in natural environments, these components do not interact in a fixed order.

Response: We thank the reviewer for approving the importance of the reaction sequence in the goethite-Hg-DOM ternary system and for the constructive suggestions. In response, we have conducted **additional measurements of the EDC of the solution phase in the addition-sequence experiments, as described in our previous response**. These data provide quantitative support that different addition orders lead to distinct DOM redox properties, consistent with the observed differences in Hg(0) production. Yes, the comprehensive characterization of goethite transformation (SRXAS), DOM fractionation (FT-ICR-MS), and full EDC/EAC evolution across all sequences is scientifically valuable. As discussed previously, we consider the sequence (i), in which Hg(II) first complexes with DOM before mineral interaction, to be an important environmentally relevant pathway, and our conclusions are therefore centered on this sequence. Detailed investigation of alternative pathways and another story will be carefully addressed in future studies.

6. Line 158-160 Liu et al reported that the Fe (oxyhydr)oxides increased the electron-accepting capacity of unbound DOM, and the authors claimed this process diminished the electron availability for Hg(II) reduction in Goethite–Hg–DOM ternary system. This attribution of Hg(II) reduction to solution-phase DOM seems to contradict the authors' later emphasis on interfacial interaction between DOM and goethite.

Response: Thanks. We clarify that the perceived inconsistency results from imprecise wording

rather than a conceptual contradiction. The discussion referring to Liu et al. describes DOM redox behavior in the absence of Hg, whereas in the ternary system considered here, **the presence of Hg modifies DOM redox properties** and favors Hg(II) reduction. In parallel, we explicitly consider **interfacial processes at the mineral surface**. Experimental observations (batch experiments and FT-ICR-MS) together with DFT simulations indicate that goethite surfaces can directly facilitate Hg(II) reduction within Hg-DOM complexes adsorbed at the mineral interface. **These two mechanisms, solution-phase DOM redox modulation and mineral-surface-mediated reduction, are therefore complementary rather than mutually exclusive.** We have revised the manuscript to clarify this distinction and to avoid potential misunderstanding. (Line 387–391 & 470–473)

7. The term "initial DOM" in Figure 2 requires a more precise definition. Specifically, it should be clarified whether this refers to a newly prepared solution, as the molecular composition of freshly dissolved organic matter (DOM) can evolve over time. Furthermore, the sample preparation protocol for FT-ICR-MS analysis, as described in the Methods section, lacks sufficient detail and should be elaborated to ensure reproducibility.

Response: Thank you for this helpful suggestion. We clarify that the term “initial DOM” refers to the DOM solution prepared for the adsorption experiments, which underwent the same preparation procedures as all other samples, including filtration and pH adjustment, before any interaction with minerals or Hg. It does not refer to freshly dissolved DOM without treatment. We acknowledge that DOM composition may evolve and have considered this. All samples analyzed by FT-ICR-MS were stored at 4°C in the dark and measured as soon as possible following preparation to minimize compositional changes. Detailed FT-ICR-MS analysis procedures are provided in the Supporting Information Texts S2–S4. We have revised the Fig. 2 caption and the Methods section to provide additional details on sample preparation for FT-ICR-MS analysis. (Line 250 & 524–525)

8. Section “DOM Molecular Fractionation in the Goethite-Hg-DOM Ternary System”, identifies the specific functional groups lost from DOM in the solution phase following its interaction with goethite. The observed fractionation suggests that DOM undergoes a structural rearrangement upon adsorption, resulting in the formation of additional electron-donating groups. However, this mechanism remains speculative without direct evidence. To confirm it, the EDC of the goethite-

adsorbed DOM must be measured directly.

Response: Thank you very much for this thoughtful comment. We acknowledge that the statement regarding the formation of additional electron-donating groups reflects a mechanistically reasonable inference rather than a direct measurement, even when analyzed by FT-ICR-MS. Previous studies have shown that adsorption of DOM onto Fe (oxyhydr)oxides can induce molecular rearrangement and alter the accessibility of redox-active functional groups, often leading to an increase in electron-accepting or donating capacities (e.g., Liu et al., 2024; Liu et al., 2025). We have conducted additional experiments in the revision to understand the changes in EDC in the solution of this tannery system, and the result aligns with our hypothesis. (Line 296–304) We also recognize that direct measurement of the EDC of mineral-adsorbed DOM is experimentally challenging and beyond the scope of the present study, which focuses on solution-phase DOM fractionation and Hg redox behavior under environmentally relevant conditions. To avoid potential ambiguity and overinterpretation, we have removed the statement regarding the formation of additional electron-donating groups. The revised text now focuses on experimentally supported observations. (Line 296–298)

Reference

Liu, Y. et al. Asymmetrical changes of electron-donating and electron-accepting capacities of natural organic matter during its interaction with Fe oxyhydroxides. Chem. Geol. 661, 122189 (2024).

Liu, K. et al. Ferrous Oxidation-Driven Adsorptive and Oxidative Transformation Boosts Electron Exchange Capacity of Dissolved Organic Matter: Environmental Implications. Environ. Sci. Technol. acs.est.5c13400 (2025).

9. Line 236-240 The emphasis on the increased EDC of the residual DOM in the solution promoting Hg(0) formation appears to contradict the authors' subsequent emphasis on the interfacial interactions between DOM and goethite.

Response: Thanks. This point has been addressed in our response to Comment 6. As clarified there, the perceived inconsistency arises from imprecise wording rather than a conceptual contradiction. Solution-phase DOM redox modulation and interfacial interactions between DOM and goethite represent complementary processes contributing to Hg(II) reduction rather than mutually exclusive mechanisms. We have revised the manuscript accordingly to clearly distinguish these processes and to avoid potential misunderstanding. (Line 387–391 & 470–473)

Thank you very much again for all the constructive comments.

Reviewer #2 (Remarks to the Author)

The authors studied the reduction of Hg in a goethite-Hg-DOM ternary system by using experimental and DFT calculations. This work is well-designed with full discussion. Nevertheless, the novelty or significance of this work in the field of mercury emission control is not very clear, which seems to be a routine work or an incremental work.

Response: Thank you very much for your helpful and constructive comments. We think that some of our initial descriptions may have led to misunderstanding and ambiguity. In the revised manuscript, we have supplemented additional experiments in response to all the reviewers' comments to substantiate our new findings and have improved the rigor of our presentation. Actually, this study does not aim to develop a technology for Hg emission control or to provide an immediate engineering solution. Instead, it seeks to improve the mechanistic understanding of Hg redox behavior in mineral-Hg-DOM systems under environmentally relevant conditions. Although Hg reduction in the presence of DOM or minerals has been reported previously, most existing studies have examined these interactions separately or focused on bulk observations, without considering how reaction sequence, DOM redistribution, and mineral interfaces act together to influence Hg redox pathways. **In this study, we have identified a novel pathway for dark Hg reduction, demonstrating that Hg bound to organic matter in water and soil can be extensively reduced to elemental Hg via the action of minerals (e.g., goethite) under dark conditions, along with elucidating the underlying mechanism. The enhanced Hg(II) reduction observed in the Hg→DOM→goethite sequence highlights the critical role of DOM mobility, fractionation, and molecular accessibility when it interacts with iron(oxyhydr)oxides.** Further, we quantified the global Hg reduction flux contributed by this hidden process. **This discovery fills a gap in the current understanding of Hg dark reduction pathways (except for the widely recognized photochemical and microbial reduction) and may hold important implications for advancing the knowledge of terrestrial Hg cycling and assessing environmental Hg risks.**

Moreover, an adsorption sequence-dependent Hg(II) reduction pathway was revealed in this study. Free DOM and iron oxides (e.g., goethite) are widely present and constitute significant forms in natural environments. For instance, in porewater, groundwater, and wetland systems, a substantial fraction of DOM remains mobile, and Hg(II) predominantly occurs as DOM-associated species even at low Hg/DOM ratios. This leads to an initial homogeneous complexation between Hg(II) and DOM, followed by heterogeneous interactions of DOM-Hg species with Fe (oxyhydr)oxide surfaces. In this work, we clarify that the sequence in which Hg(II) is pre-

complexed with DOM before interaction with goethite represents a common and environmentally realistic pathway in the environment, particularly in DOM-rich environments such as wetlands, peatlands, and anoxic sediments. DOM-Hg complexation proceeds rapidly in homogeneous solution and is thermodynamically dominant, with dissolved Hg occurring predominantly as DOM-associated species under environmentally relevant Hg/DOM ratios. In contrast, reactions involving mineral-bound DOM are heterogeneous and constrained by surface accessibility, and adsorption of DOM onto mineral surfaces substantially reduces the availability of functional groups involved in metal binding and electron transfer.

Our data show that even after Hg-DOM equilibration, free DOM, iron(oxyhydr)oxides, and DOM-Hg complexes coexist in solution. In this context, goethite enables dynamic competition for surface sites and distinct interfacial reaction pathways. The inhibition of Hg(II) reduction observed when DOM is pre-adsorbed onto goethite indicates that surface immobilization of DOM limits the availability of redox-active functional groups and constrains interfacial electron transfer. Conversely, the enhanced Hg(II) reduction observed in the Hg→DOM→goethite sequence highlights the critical role of DOM mobility and molecular accessibility. By providing a clearer process-level framework for interpreting Hg stabilization and reduction at mineral-organic interfaces, our results may inform assessments of Hg mobility and Hg(0) formation in soils and aquatic environments. We have revised the manuscript accordingly to better reflect this novelty and to avoid overstating broader implications.

1.Introduction section, the hazard of mercury should be introduced. The latest laws or regulation for mercuy emission or discharge could be discussed as well.

Response: Thank you for the suggestion. The Introduction has been revised to include a clearer description of the environmental and health hazards associated with Hg. (Line 36–41)

2.The authors should add more texts to introduce or emphasize the novelty of this work, especially in new fundamental knowledge, new general methods, etc.

Response: The Introduction has been revised to better emphasize the novelty of this study, particularly the new mechanistic insights into Hg redox behavior in mineral-Hg-DOM systems and the broader relevance of the experimental-theoretical framework employed. (Line 52–59 & 88–94

& 98–104)

3.Line 251, the reason for the intensity reduction of the (110) and (020) diffraction peaks could be discussed more by citing pertinent literature.

Response: Thanks for the suggestion. Pertinent literature has been added to explain the observed reduction in the intensity of the (110) and (020) diffraction peaks. (Line 318–320)

4.Fig. 3 f and g, there are too many curves being compacted in the figures, which are too blurry and hard to follow. It is better to scale up them to make it clearer.

Response: Thanks a lot for the suggestion. Figs 3f and 3g are presented in a stacked format to facilitate direct comparison of peak positions and relative intensities across treatments. To be clearer, we have provided an enlarged, scaled-up version of these plots in the Supporting Information (Extended Figure Supplementary Fig. S9), where the curves can be more clearly distinguished.

Extended Figure Supplementary Fig. S9:

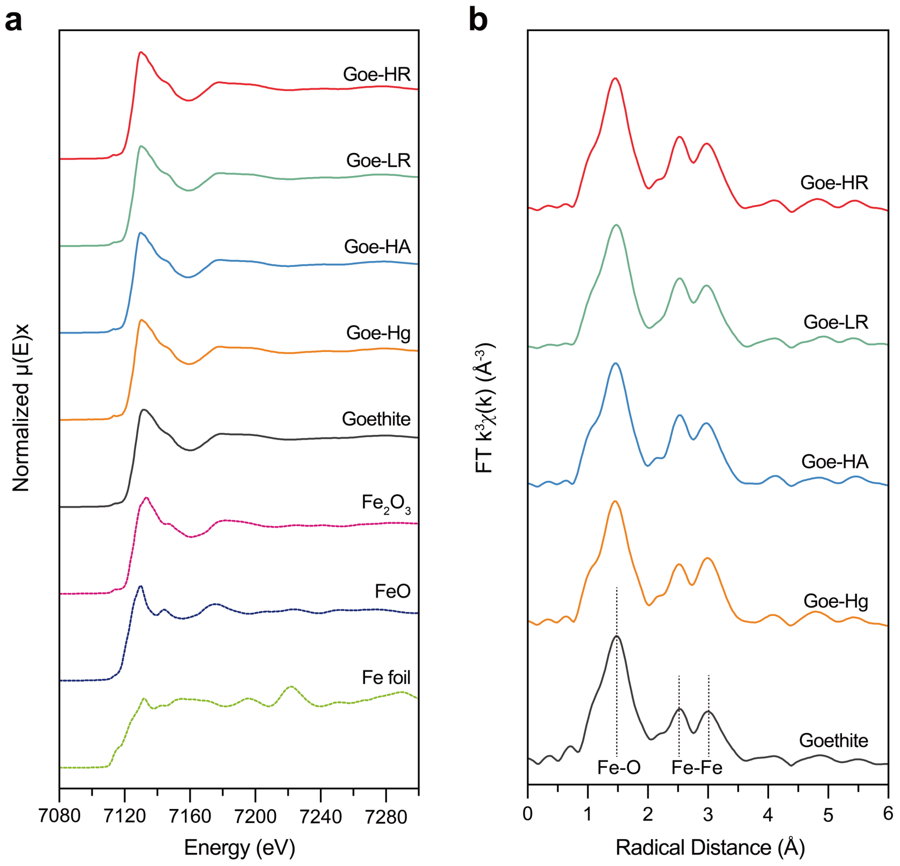

5.It is suggested to add a schematic or photo for the sorption experiments.

Response: Thanks. A schematic illustration of the sorption experiments has been added as Modified Fig. 1a to improve clarity.

Modified Fig. 1:

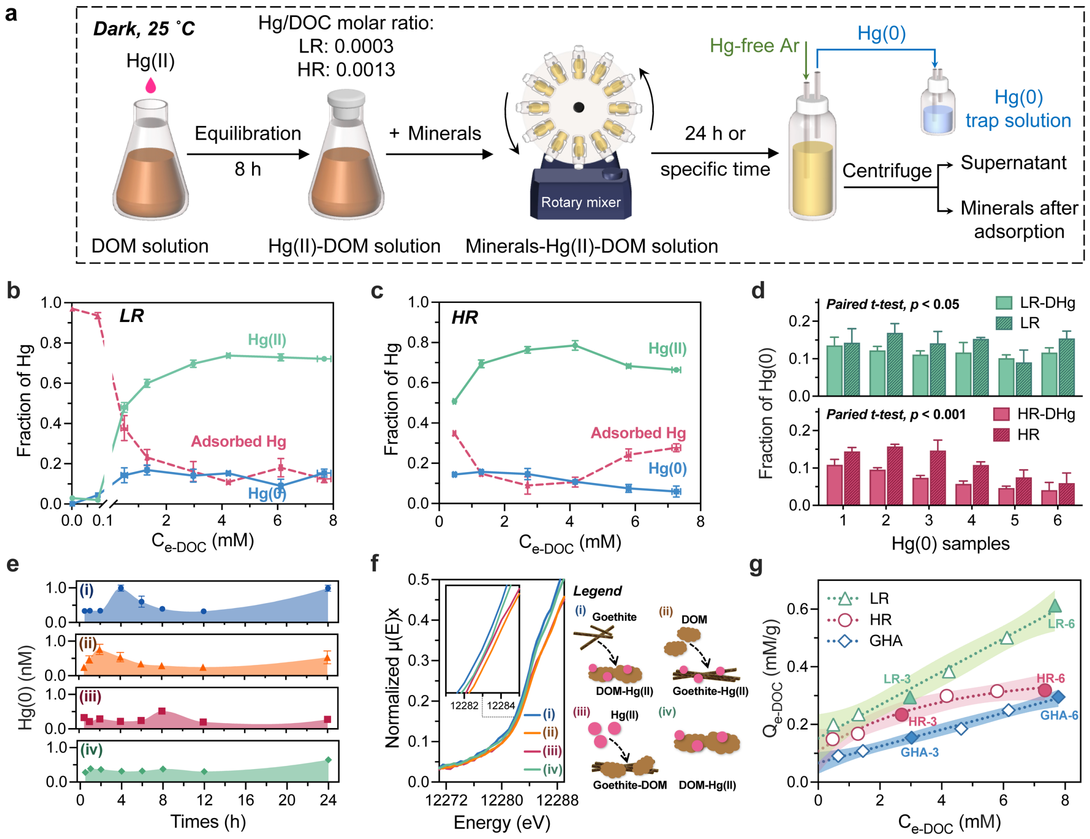

Thank you very much again for all the helpful and constructive comments.

Reviewer #3 (Remarks to the Author)

Overall Summary:

This manuscript presents a comprehensive investigation into a previously overlooked pathway for dark reduction of Hg(II) in mineral-Hg-DOM ternary systems. The study is well-structured, employing a combination of batch experiments, advanced spectroscopic techniques (FT-ICR-MS, XAS), and DFT calculations. Overall, I think the findings are significant and could substantially advance our understanding of the global mercury cycle. However, several aspects regarding the mechanistic evidence, experimental design, and data interpretation require clarification before the manuscript can be accepted for publication.

Response: Thank you very much for your time and helpful comments in improving our manuscript.

We have addressed all the concerns raised regarding mechanistic interpretation, experimental design, and data presentation in the revised manuscript and the Supporting Information. Specific responses to each comment are provided below.

Specific comments:

1. Lines 177-180: The terms "GHA-3", "GHA-6", "LR-3", "LR-6", "HR-3", and "HR-6" are used to denote specific samples for FT-ICR-MS analysis but are not explicitly defined in the main text or figure caption for Fig. 1f. Please clearly state what these suffixes (e.g., -3, -6) represent.

Response: Thanks. The definitions of the sample labels have now been explicitly clarified in both the main text and the Fig. 1 caption. (Line 132–135 & 141–142) Numbers 1–6 refer to the six isothermal adsorption experiments and follow the same left-to-right order as the data points shown in panels b and c, while the suffixes "-3" and "-6" denote the third and sixth samples collected from the isothermal adsorption experiments, respectively.

2. Lines 128-130: The decline in Hg(0) yield after 4 hours in sequential experiment (i) is attributed to "DOM-mediated reoxidation." While this is a hypothesis, no direct evidence is provided to confirm that the decrease in Hg(0) is due to its re-oxidation back to Hg(II) (e.g., by tracking the re-appearance of aqueous Hg(II) or identifying the oxidized Hg species). Please provide supporting data for this claim or temper the language to present it as a potential explanation among others.

Response: Thank you for the suggestion. According to this comment, we need to explain that the measured Hg(0) reflects the net effect of simultaneous Hg(II) reduction and Hg(0) re-oxidation, along with ongoing structural evolution of Hg-DOM complexes. Given the complexity of these coupled processes, we have provided a plausible interpretation based on the available data and published research. According to Jiang et al. (2015), in the DOM-Hg system, the late-stage Hg(0) increase can be explained by time-dependent structural rearrangement of Hg-DOM complexes. Hg(II) initially binds to O/N-rich functional groups, leading to relatively rapid reduction but also rapid Hg(0) re-oxidation. As these O/N sites are progressively consumed, Hg(II) becomes increasingly coordinated by S-containing groups, which slows reduction yet also diminishes Hg(0) re-oxidation, allowing Hg(0) to accumulate in the later reaction interval. In the ternary systems, the behavior is further influenced by DOM-mineral interactions.

Additionally, we performed measurements of EDC and DOC at 2, 12, and 24 h, the results show that all addition sequences exhibit an increase in solution-phase EDC and DOC at 24 h (Extended Data Supplementary Fig. S6a). This pattern suggests that a portion of DOM molecules returned to the aqueous phase at the later stage of reaction, which is consistent with previous findings that non-carboxyl-rich DOM fractions readily desorb from mineral surfaces (Oren and Chefetz, 2012). Such late-stage DOM release increases the pool of electron-donating functional groups in solution and provides a plausible basis for the renewed Hg(0) formation. Overall, we emphasize that these explanations represent the most reasonable interpretation grounded in our experimental observations and the literature, given the inherent complexity of the system. The relevant EDC/DOC data and explanatory text have been incorporated into the Results and Discussion of the revised manuscript. (Line 179–189)

Extended Data Supplementary Fig. S6:

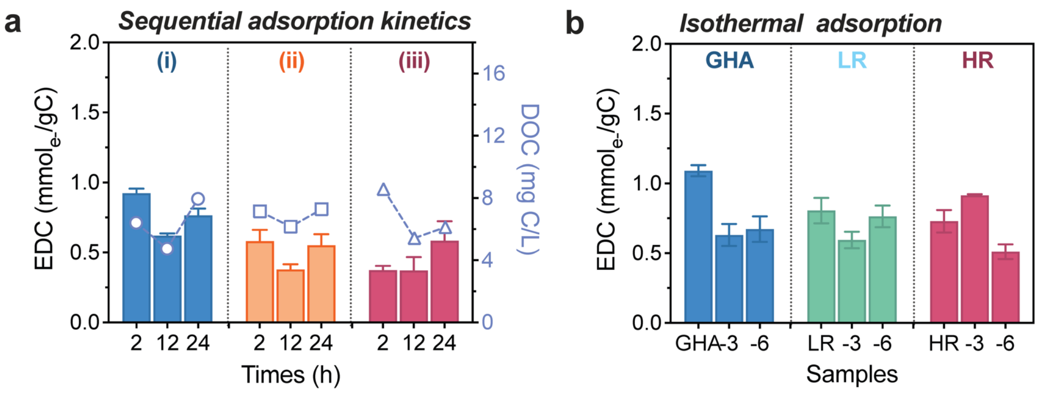

Reference

Jiang, T. et al. Modeling of the structure-specific kinetics of abiotic, dark reduction of Hg(II) complexed by O/N and S functional groups in humic acids while accounting for time-dependent structural rearrangement. Geochim. Cosmochim. Acta 154, 151–167 (2015).

Oren, A. & Chefetz, B. Sorptive and Desorptive Fractionation of Dissolved Organic Matter by Mineral Soil Matrices. Journal of Environmental Quality 41, 526–533 (2012).

3. Lines 265-275: The XAS data (XANES and EXAFS) suggest a partial reduction of Fe(III) in goethite. It appears that the formation of Fe(II), which is a critical product of the proposed electron transfer from DOM, is not directly confirmed. To solidify this key part of the mechanism, it would be highly beneficial to provide direct evidence for Fe(II) presence on the solid phase after reaction

or by marking the characteristic pre-edge features of Fe(II) and Fe(III) in the XANES spectra (Fig. 3f).

Response: Thanks for the constructive suggestion. Yes, the XAS data (XANES and EXAFS) show a clear decrease in the average Fe oxidation state in goethite after reaction, which aligns with the formation of Fe(II) in the solid phase. To make this point clearer, <u>we have revised Fig. 3f by marking the characteristic spectral features associated with Fe^{2+} and Fe^{3+} in the XANES region</u>. These additions help clarify the interpretation of the XAS results and the associated Fe redox changes discussed in the text.

Revised Fig. 3:

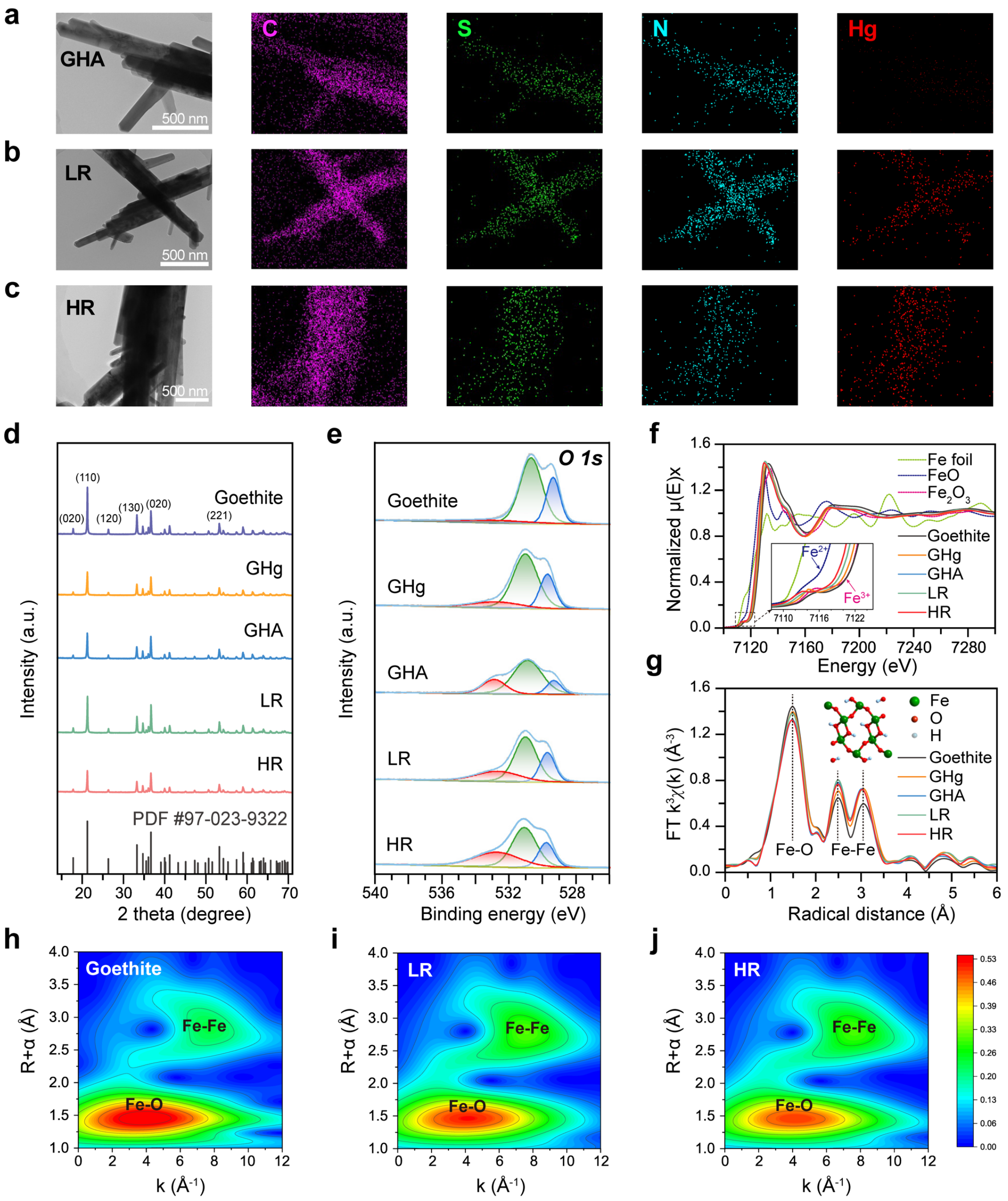

4. Lines 86-91 & 427-429: The study focuses on goethite and briefly compares it with hematite and magnetite. Given the diversity of iron (oxyhydr)oxides in the environment (e.g., ferrihydrite, lepidocrocite), it would be nice to discuss the environmental implications where other minerals dominate.

Response: Nice suggestion and thanks. We have added a brief discussion at the beginning of the Results and Discussion section to clarify the environmental relevance of focusing on goethite. (Line 110–114) In particular, we emphasize that goethite is a widespread and commonly dominant Fe oxyhydroxide in weathered soils and sediments, which makes it a representative mineral phase for the processes examined in this study.

5. Lines 86-87: The use of a single type of humic acid (HA) as a DOM proxy may limit the scope of the findings. Natural DOM is highly heterogeneous. Please acknowledge this limitation in the discussion and suggest that future work should validate these processes with DOM from various environmental sources.

Response: Thanks for the helpful and constructive suggestion. We have acknowledged this limitation in the section "Pan-environmental significance of mineral-Hg-DOM ternary systems on the global Hg cycle", (Line 457–462), noting that the use of a single humic acid as a DOM proxy does not capture the full heterogeneity of natural DOM. We also emphasize that future studies should validate the observed processes using DOM from diverse environmental sources.

6. Lines 289-291: The use of cysteine and salicylic acid as model compounds for DOM is a necessary simplification. However, the discussion should more explicitly state the limitations of this approach, as it cannot capture the complexity, cooperative effects, and steric constraints of a macromolecular DOM matrix. Please elaborate on how these simplifications might affect the interpretation of the binding energies and reduction pathways.

Response: Thanks for the suggestion. This limitation has been explicitly acknowledged in the revised Discussion. (Line 361–369) We clarify that Cys and SA are used as simplified model compounds to probe functional-group-level interactions, and that the resulting binding energies and reduction pathways should not be directly extrapolated to macromolecular DOM systems.

7. Lines 357-369 & 471-484: The global scaling exercise is a valuable addition. However, the parameterization introduces significant uncertainty. The binary classification of the residual Hg fraction (f) solely based on a total Hg concentration threshold of 0.25 μM is a major simplification, as Hg speciation is influenced by numerous local factors (soil type, organic matter, redox conditions). The discussion should clearly acknowledge this aspect.

Response: We do agree with this, and thanks for the kind suggestion. We have revised the Discussion to explicitly acknowledge that the binary classification of the residual Hg fraction represents a necessary simplification for the global scaling analysis. (Line 434–439) We also clarify that this approach is not intended to capture site-specific Hg speciation, which is influenced by local environmental factors such as soil properties, organic matter, and redox conditions, and that the associated uncertainty is recognized.

8. Lines 196-197 & Fig. 2: The non-detection of carbohydrate-like molecules (group 3) is noted. This might be an artifact of the sample preparation (PPL solid-phase extraction) and ionization technique (negative ESI). Please add a sentence in the methods or figure caption to clarify that the FT-ICR-MS results represent the "SPE-amenable, ESI-ionizable" fraction of DOM, not its total composition.

Response: Thanks for the suggestion. A clarifying statement has been added to the Fig. 2 caption indicating that the FT-ICR-MS data represent the SPE-amenable, ESI-ionizable fraction of DOM. (Line 256–258)

9. Fig.6a and b: Please check the font of the axes.

Response: Thanks. The axis fonts in the original figures are correct; the distortion occurred during PDF generation from the Word file. We have revised the figure insertion format to prevent this issue.

Point-by-Point Response to the Reviewers

(For manuscript *NCOMMS-25-79362A-Z*)

We are grateful for the valuable comments provided by the reviewers and appreciate your constructive suggestions for further improving this manuscript. Below, we present our point-by-point responses. Please note that our responses to the reviewers' comments are indicated by blue text, reviewer comments are in *italics*, and changes to the manuscript are in the normal 11pt font. Major revisions are highlighted in yellow in the manuscript.

Reviewer #4 (Remarks to the Author)

This study identifies a missing dark mercury reduction pathway operating in mineral-Hg-DOM ternary systems and fills an important gap in our understanding of the global mercury cycle. The authors present a clear and rigorous body of work that combines laboratory experiments, FT-ICR-MS, synchrotron-based XAS, DFT calculations, and global-scale modeling. The study is well organized, the figures are clear and informative, and the proposed coupling between goethite transformation and DOM fractionation is strongly supported by the data.

I have personally been involved in research on dark mercury reduction. In previous efforts to quantify microbial-mediated Hg reduction under dark conditions, we repeatedly encountered mass-balance discrepancies in which experimental results failed to match with field observations. The field has explored microbial pathways and gene-related processes, such as the possible roles of merA, but their contribution under dark conditions appears limited and often inconsistent. In both aquatic and soil systems, these processes have not provided a clear explanation for observed mercury reduction. This study offers a simple and convincing abiotic explanation. It shows that mercury can be reduced through coupled reactions between iron mineral transformation and DOM fractionation at the solid-water interface. This mechanism can operate in the absence of light and does not rely on specific biological conditions. It therefore provides a realistic explanation for mercury reduction in environments such as deep-sea sediments and subsurface soils, where iron minerals and DOM are common.

The novelty and broad environmental relevance of this mechanism are significant. Importantly, it

provides a robust and environmentally realistic explanation that is applicable across both soil and aquatic systems, substantially advancing our predictive understanding of mercury cycling under dark conditions. The comments raised by the previous reviewers were generally constructive and appropriate for a study of this scope. The authors have responded well and have revised the manuscript accordingly, resulting in a clearer, more coherent, and more persuasive presentation of the work. The remaining issues are minor and primarily relate to clarity and framing rather than scientific validity. I therefore recommend acceptance of this manuscript after minor revision.

Response: Thank you for your recognition of the novelty of this work and the constructive suggestions. We have revised the manuscript accordingly and addressed the specific points below.

Specific Comments:

1. Fig. 5 has a strong summary of this work. Adding a short guiding sentence in the text when first referring to the conceptual figure would improve readability.

Response: We added a short guiding sentence when Fig. 5 is first introduced to orient the reader to the conceptual summary. The revised text is shown in Lines 414–416.

2. Please clarify whether surface Fe(II) is expected to directly reduce Hg(II) or mainly support DOM-mediated electron transfer.

Response: Based on the added aqueous Fe(II) data and the mineral comparison, we do not interpret Fe(II) as the primary direct reductant responsible for the enhanced Hg(0) production in the goethite-Hg(II)-DOM ternary system. Instead, Fe redox changes appear to accompany the coupled interfacial process, whereas the enhancement in net Hg(0) production is more consistent with mineral-induced DOM fractionation and Hg speciation changes. This interpretation is supported by two observations. First, net Hg(0) production across the mineral comparison and sequential-addition experiments tracked EDC more closely than aqueous Fe(II). Second, goethite enhanced Hg(0) production in the ternary system despite lacking structural Fe(II) before reaction, whereas the stronger Fe(II) signal in the magnetite system did not produce a corresponding ternary enhancement. We have added the aqueous Fe(II) data to revised Supplementary Fig. 4

and clarified this point in Lines 162–173.

3. The experiments were conducted at pH 5.5. A brief note on how higher or lower pH might affect ternary complex stability would help extend the environmental relevance.

Response: We note that the stability and partitioning of mineral-Hg(II)-DOM associations may vary with pH, because protonation and surface-charge changes can shift the balance between mineral-associated Hg-DOM species and dissolved Hg-DOM complexes. We also clarify that all experiments were conducted at pH 5.5, which should be treated as a boundary condition of the present study. These revisions are included in Lines 381–384 and 477-483.

4. A short clarification on whether light exclusion was strictly maintained throughout the experiments would improve transparency.

Response: We have clarified in the revised Methods that all reactions were carried out under light-protected conditions, with rapid sample transfer and filtration to minimize incidental exposure. We also state explicitly that Hg(0) was first purged and trapped before centrifugation and filtration, so the reported Hg(0) production was not affected by the later handling steps. The revised text can be found in Lines 511–513.

5. The mechanism you've discovered has clear implications beyond topsoil. A short sentence highlighting why this mechanism matters beyond soils, for example in sediments or subsurface systems, would strengthen the opening.

Response: We added a sentence in the Introduction noting that the same interfacial mechanism may also be relevant in sediments and subsurface environments where Fe minerals, DOM, and Hg coexist. We also clarify that our quantitative estimate was restricted to topsoils because they represent the main zone of soil-atmosphere Hg exchange. These revisions are included in Lines 77–79 and 435–437.

6. The study convincingly demonstrates that mineral transformation and DOM fractionation are coupled rather than independent processes. The manuscript already provides a clear explanation of

this coupling, which effectively prevents readers from interpreting these effects as parallel but unrelated events.

Response: We appreciate this comment and are glad that the coupling between mineral transformation and DOM fractionation is now clear in the revised manuscript.

7. The comparison between different iron minerals is informative. A short clarification on whether mineral reactivity or surface chemistry is the dominant control would strengthen the mechanistic interpretation.

Response: Based on the mineral comparison, we do not attribute the observed differences in the ternary system to intrinsic mineral reactivity alone. Instead, mineral-specific surface chemistry appears to be the more immediate control because it governs how DOM and Hg interact with the surface, how DOM is fractionated, and ultimately how Hg(II) reduction proceeds in the ternary system. We have clarified this point in Lines 386–390 and 393–400.

Reviewer #5 (Remarks to the Author)

This manuscript investigates Hg redox transformations in a Hg–DOM–goethite ternary system and provides interesting insights into the coupled interactions among Hg, dissolved organic matter (DOM), and Fe (oxyhydr)oxides. In particular, the study suggests that adsorption of DOM onto goethite can modify the redox characteristics of the remaining aqueous DOM, which may subsequently influence Hg redox transformations. The authors also propose that Hg–goethite–DOM ternary complexes may be more susceptible to Hg(II) reduction compared with Hg–DOM complexes alone. These observations are potentially important because they highlight how mineral–DOM interactions can indirectly regulate Hg speciation and redox behavior in natural environments.

However, several conceptual interpretations, experimental design choices, and descriptions of Hg speciation require further clarification and discussion. In particular, the novelty of the proposed "missing reduction pathway", the role of thiol-controlled Hg speciation, and the interpretation of Hg reduction and reoxidation processes in the experimental system need to be more carefully evaluated.

Response: Thank you very much for your constructive comments on this work and for your recognition of its importance. We believe that by incorporating your suggestions, the article is now further enhanced in both rigor and scientific quality. In particular, in the revised manuscript, we have moderated the wording related to novelty, clarified the basis for our discussion of Hg binding under different Hg to DOM conditions, revised the discussion of Hg(0) production to distinguish more clearly between net accumulation and the underlying balance of reduction and reoxidation, and improved the description of the experimental design in the Methods. Overall, we have further highlighted the novelty of this study and tightened the wording in some places to enhance the rigor based on the available data, as per your suggestion. These points are addressed individually below.

Major comments

1. The title "A missing reduction pathway of Hg by coupled reactions in mineral–Hg–DOM ternary systems" is somewhat misleading. I do not think the process described here represents a "missing" reduction pathway. Surface-mediated reduction of heavy metals on Fe (oxyhydr)oxides, both in the absence and presence of DOM, has been widely studied in environmental geochemistry, for example, As, Cr, and Cd on Fe minerals have been extensively reported. In many of these systems, Fe(II) plays a key catalytic role by promoting mineral transformation of Fe (oxyhydr)oxides and facilitating electron transfer processes that lead to metal reduction. Therefore, the mechanism discussed in this manuscript is not fundamentally new. The apparent novelty may arise from the fact that similar processes have been much less studied for Hg compared with other heavy metals. Nevertheless, the underlying mechanisms are likely similar.

By the way, one important reason why fewer studies on Hg compared with other metals is the typically low environmental concentration of Hg. For many metals, their speciation in nature is largely controlled by surface reactions with Fe (oxyhydr)oxides, including adsorption or surface precipitation. In contrast, Hg speciation is more strongly controlled by complexation with DOM. Because Hg concentrations are usually extremely low, even relatively small amounts of DOM can dominate Hg speciation. Hg has a very strong affinity for thiol groups in DOM. Under environmentally relevant Hg concentrations, Hg typically forms $Hg(DOM\text{-}RS)_2$ complexes with

thiol groups. At higher Hg concentrations where thiol groups become saturated, Hg may also bind to oxygen-containing functional groups (such as carboxyl groups). In sulfidic environments, Hg forms HgS phases. Only under very certain conditions does Hg significantly interact with Fe (oxyhydr)oxide surfaces.

Response: Thank you for this important and insightful comment. We agree that the phrase "missing reduction pathway" could imply a fundamentally new class of surface-mediated metal reduction, and that was not our intention. We have therefore revised the title to "An unexplored coupled process enhancing dark Hg(II) reduction in mineral-Hg(II)-DOM ternary systems." This wording better reflects the scope of our study and avoids overstating the broader novelty of the process.

As you aptly noted, unlike other heavy metals, Hg is unique in its multiple valence states and physical phases, and quite sensitive to environmental conditions. This leads to highly complex environmental processes of Hg, which have long made it difficult to fully understand Hg transformation and the key impactors. Thus, the transformation processes and mechanisms described for other metal systems could not be directly transferred to Hg. We guess Hg interacts with DOM and mineral surfaces in a different way in soils, leading to its unique mode of migration and transformation. Hg is commonly bound first by DOM, especially by thiol-containing sites, before further association with mineral surfaces becomes important. Our intention in this study, therefore focus on clarifying how this coupled process operates specifically in the complex Hg system. In most natural settings, Hg speciation is controlled primarily by complexation with DOM, especially at low and environmentally relevant Hg concentrations. This consideration was already built into our experimental design. We were not assuming that free Hg(II) commonly interacts directly with mineral surfaces as the dominant environmental pathway. Instead, we focused on a more environmentally realistic situation in which Hg is first associated with DOM in solution, and these Hg-DOM complexes then encounter mineral surfaces. This is also why we examined two Hg/DOC conditions and included both sulfur-containing and oxygen-containing ligands in the DFT calculations.

To make this point clearer and avoid misunderstanding, we revised the related text

throughout the manuscript, including the Title, Abstract, Introduction (Lines 58–61 and 96–98), and the Discussion sections (Lines 216–218, 414–416, 493, and 497). All the revisions are highlighted.

2. The authors used goethite as the Fe mineral in their experiments. However, in natural environments different Fe minerals dominate under different redox conditions. Under oxic surface conditions, ferrihydrite, goethite, and hematite are commonly present. Among which, ferrihydrite is much more abundant than goethite. Ferrihydrite is poorly crystalline and typically exhibits much higher adsorption capacity and greater reactivity. It also undergoes Fe(II)-catalyzed mineral transformations more readily, making it highly relevant for redox reactions involving electron transfer. Under more reducing conditions, Fe sulfides such as FeS and FeS_2 *are typically dominant, while intermediate redox conditions often contain magnetite or green rust phases. These minerals can be more environmentally relevant for dark reduction processes. In contrast, Hg(II) reduction under oxic surface conditions is often associated with photochemical processes. Given these considerations, it would be useful for the authors to justify the choice of goethite. It is also worth discussing why ferrihydrite or other more reactive Fe minerals were not selected, as they may play a more important role in environmental Hg transformations.*

Response: Thank you for the helpful comment. We agree that different Fe minerals can influence Hg transformation differently across redox gradients, and that ferrihydrite, mixed valence Fe minerals, and Fe sulfides are all important in natural systems. This comment helps us further clarify how the environmental relevance of our mechanism should be framed.

Goethite was selected because it is widespread and persistent in soils and sediments, and its structural stability allows interfacial mineral-Hg(II)-DOM reactions to be examined without the additional complexity introduced by rapid phase transformation. This was important for the present study, which aimed to resolve how DOM fractionation, Hg coordination, and mineral association are coupled in this ternary system. We agree that ferrihydrite is environmentally important and often more reactive than goethite in typical conditions. In the presence of Fe(II) and organic matter, ferrihydrite can readily undergo transformation, surface restructuring, and

coprecipitation. In such a system, changes in Hg behavior would reflect not only mineral-Hg(II)-DOM coupling, but also concurrent mineral evolution, surface restructuring, and Fe-OM coprecipitation. In the revision, we have enhanced the related discussion on this point based on the available data.

Goethite is one of the most well-established mineral systems for studying DOM adsorption and molecular fractionation. It also provides a useful reference for interpreting how mineral-associated DOM fractionation may influence Hg transformation in other systems. In our view, the differences among minerals arise not only from their structural properties and intrinsic reactivity, but also from their selective retention of specific DOM components. Such differences can alter the composition of the remaining aqueous DOM, its electron-donating and electron-accepting capacities, its photo-reactivity, and its coordination with Hg, which may in turn lead to different extents of Hg redox transformation. Our comparison with hematite and magnetite within the same ternary framework is consistent with this interpretation. The observed differences in Hg(0) production were closely related to changes in DOM electron-donating capacity, suggesting that different mineral surfaces produced different DOM partitioning patterns and, in turn, different Hg coordination and redox behavior. In this context, the goethite system provides a clear baseline for understanding how mineral-specific DOM fractionation may translate into mineral-specific Hg redox behavior. We have added a clarification of the rationale for selecting goethite in the revised manuscript in Lines 108–113 and 171–173.

Moreover, we agree that FeS and FeS_2 are important in more reducing environments. From the perspective of DOM partitioning and molecular fractionation, similar effects may occur across different Fe mineral interfaces. A previous study reported that Fe sulfides and Fe hydroxides can produce broadly similar DOM fractionation patterns (Wang et al., 2019). Fe sulfides also involve distinct Hg-S coordination and interfacial pathways that differ from those of Fe oxyhydroxides. It is another complex system, differing from this study, that needs further investigation.

Regarding photochemical reduction, all experiments here were conducted under dark conditions to isolate the contribution of mineral-DOM interfacial reactions. This

was not intended to downplay the importance of photoreduction in oxic surface environments. Rather, the present study focuses on a complementary dark pathway that may operate where Fe minerals and DOM coexist (e.g., in soils and sediments scenarios). We would also note that the mechanism identified here may be relevant to photo-reduction, because mineral adsorption changes DOM composition, and different DOM fractions differ in photo-reactivity and coordination with Hg. We have clarified this point in the revised manuscript in Lines 469–472 and 477–479.

Finally, we thank the reviewer for raising the questions of ferrihydrite and more reduced Fe mineral systems. The ferrihydrite-related aspect is already part of our ongoing work, particularly in relation to Hg speciation and transformation during Fe-OM coprecipitation (may be more challenging). We see this as a natural extension of the present study, as these systems likely involve additional controls from mineral instability, phase transformation, and organic matter association. Hg reactions at Fe sulfide interfaces may also follow pathways distinct from those at Fe oxyhydroxide interfaces and are well worth further investigation. Your comments have helped us clarify the scope of the present study and identify useful directions for future work.

Reference: Wang, Y. et al. Preferential molecular fractionation of dissolved organic matter by iron minerals with different oxidation states. Chem. Geol. 520, 69–76 (2019).

3. The Hg–DOM–goethite ternary system is very complex. In the simpler Hg–DOM system, multiple processes occur simultaneously. Hg can complex with DOM through both thiol (RSH) and oxygen-containing functional groups (ROH), depending on the Hg/DOM ratio. These complexes have very different thermodynamic stabilities and reactivity especially on Hg(II) reduction. DOM can also act as both electron donors and electron acceptors, can reduce Hg(II) to Hg(0) but also in parallel, oxidize Hg(0).

In the Hg–goethite system, Hg(II) may form surface complexes with goethite. When Fe(II) is present, Fe(II) can catalyze mineral transformation of goethite into mixed Fe(II)–Fe(III) (oxyhydr)oxide phases, which may enhance electron transfer and Hg(II) reduction. Bridging structures involving metal–DOM–Fe (oxyhydr)oxides have been widely reported for other metals. Therefore, when studying Hg–DOM–Fe mineral systems, it is important to first understand the simpler subsystems

and how DOM and Fe minerals evolve during the reaction. For example, Fe mineral transformations should be monitored by tracking both aqueous and adsorbed Fe(II), since Fe(II) strongly influences mineral transformation pathways. The relative abundance of Fe(II) and mixed Fe(II)–Fe(III) phases can significantly influence the redox properties of Fe minerals. It said in the Abstract "resulting in newly formed electron shuttles, electron donors, and iron redox couples" but the electron shuttles, Fe(II) concentration and Fe-mineral transformation, etc were not displayed. In addition, the production of Hg0 observed in the experiments represents the net result of reduction and oxidation processes. Therefore, the potential reoxidation of Hg0 should also be considered when interpreting the results. For example, the observed Hg0 production may underestimate the actual extent of Hg(II) reduction, because a portion of the generated Hg0 could have been reoxidized, rather than indicating that Hg(II) (in the presence of goethite) was less stable.

Response: Thank you for these thoughtful comments. We agree that the Hg(0) observed in our experiments should be interpreted as the net result of coupled reduction and oxidation processes. This point had already been considered in the manuscript, but we agree that it was not stated clearly enough. We have therefore revised the relevant sentence, together with the associated discussion and description of the adsorption results (e.g., Lines 115–116), to make this interpretation more explicit in the revised manuscript. In the revised text, the higher Hg(0) observed at 24 h is interpreted as net Hg(0) accumulation arising from the evolving balance between Hg(II) reduction and Hg(0) reoxidation, together with time-dependent rearrangement of Hg(II) among different ligand environments and changes in DOM-mineral interactions.

We agree that simpler subsystems contribute to the interpretation of the mechanisms of the ternary system. For this purpose, our experimental design includes a complete control experiment of the binary system, and separately characterized both the DOM and Fe minerals to clarify their evolution after reaction. For DOM, we used FT-ICR-MS to examine molecular composition changes and measured EDC to evaluate changes in redox-donating capacity. For minerals, we performed complementary characterization using XRD, XPS, XAS, TEM, and EDS. However, we were not able to continuously monitor and quantify the relative abundance of mineral-associated Fe(II) and Fe(III) throughout the reaction process, because time-resolved tracking of surface

Fe speciation across multiple minerals would require repeated advanced analyses beyond the scope of this study.

Following the reviewer's suggestion, we have added aqueous Fe(II) data from the kinetic experiments to the Supporting Information (revised Supplementary Fig. 4 d–f). While these data do not fully resolve the Fe redox process, **they help to indicate that changes in aqueous Fe(II) are not the main factor controlling the net Hg(0) production**. In both the mineral comparison and sequential addition experiments, the trend in net Hg(0) production was more closely related to EDC than to aqueous Fe(II). In this context, the role of minerals is better interpreted as facilitating Hg(II) reduction through changes in DOM redox availability and interfacial Hg configuration, rather than through Fe(II) accumulation itself. This interpretation is also consistent with our DFT results, which show that goethite lowers the reaction energy barrier for Hg(II) reduction. We have revised the manuscript accordingly (in Lines 162–171 and 352–355) to make this point clearer and to avoid overstating the role of Fe redox transformation. We have also revised the corresponding wording in the Abstract to avoid potential misunderstanding. In addition, following your suggestion, we have revised the discussion of the environmental implications (in Lines 184–187 and 460-461) to better reflect the complexity of Hg(0) production in natural systems and to frame these implications more cautiously.

Specific comments

1. Line 108–109: The authors define two Hg/DOC molar ratios. However, because Hg speciation is largely controlled by thiol groups, the Hg/thiol ratio would be a more relevant parameter, therefore should be displayed also.

Response: Thanks. We have provided a more detailed description in the revised manuscript and made changes to the text for clarity. The revised text now more clearly explains how the abundance of reduced sulfur sites and thiol groups was estimated and how this information was used to interpret Hg binding under the LR and HR conditions. Please find the revised text in Lines 519–527.

2. Line 120: The reduction of Hg(II) by DOM is regulated by both the reducing capacity of DOM (electron-donating functional groups) and the speciation of Hg(II).

Response: This sentence has been revised in Lines 121–123 to clarify the complete factors governing Hg(II) reduction by DOM.

3. Figure 1: In panels b and c, does Ce-DOC represent the DOC concentration in the supernatant after the 24-hour reaction shown in Fig. 1a? If so, the authors should demonstrate that 24 h is sufficient to reach equilibrium; otherwise, these concentrations cannot be interpreted as equilibrium values.

Response: Thank you for pointing this out. Yes, $C_{e\text{-DOC}}$ refers to the DOC concentration in the supernatant after the 24 h reaction shown in Fig. 1a. The Methods section already states that 24 h was sufficient for both DOM and Hg fractions to reach equilibrium, based on previous studies and our kinetic measurements (Supplementary Fig. 1), and this information is provided in Lines 530–532.

4. Line 173: "A third reactant was introduced 2 h after their initial reactions." Why was 2 h selected? A longer equilibration time (e.g., 24 h) might be more appropriate to ensure that the initial reactions have reached equilibrium.

Response: Thanks. The 2 h interval was selected because it was sufficient for the initial binary reactions to reach equilibrium or a near-stable state before the third reactant was introduced. In our goethite-Hg(II) system, adsorption equilibrium was reached within 2 h (Supplementary Fig. 1); in the Hg-DOM system, equilibration can be assessed from the stabilization of Hg(0) production (Fig. 1e); and in the goethite-DOM system, previous work has shown that DOM molecular fractionation on goethite had already reached equilibrium after 2 h (Coward et al., 2019). Taken together, these results indicate that 2 h represents a reasonable equilibration time for the relevant binary systems in our sequential addition experiments. A longer pre-reaction time, such as 24 h, was therefore not necessary for establishing the initial binary equilibrium state. We have clarified this point in the Methods section in Lines 541–544.

Reference: Coward, E. K., Ohno, T. & Sparks, D. L. Direct Evidence for Temporal Molecular

Fractionation of Dissolved Organic Matter at the Iron Oxyhydroxide Interface. Environ. Sci. Technol. 53, 642–650 (2019).

5. Line 184: "slows both Hg reduction and re-oxidation, allowing Hg(0) to accumulate over time". This interpretation may be incorrect. The system is closed, and the Hg(II) ⇌ Hg(0) transformation is a reversible reaction. The reduction of Hg(II) depends on electron donors and Hg(II) speciation (e.g., Hg-thiol vs Hg-carboxyl). Meanwhile, DOM may also contain electron-accepting groups capable of oxidizing Hg(0). The oxidation of Hg(0) depends on both the concentration of electron acceptors and the concentration of dissolved Hg(0). Dissolved Hg(0) is governed by Henry's law equilibrium with Hg(0) in the headspace. When Hg(0) accumulates in the headspace of the reaction vial, the concentration of dissolved Hg(0) may increase, potentially promoting re-oxidation to Hg(II).

Response: Thanks. We have revised this discussion in Lines 184–194 to avoid an oversimplified interpretation of Hg redox dynamics in the closed system. Accordingly, we interpret the higher Hg(0) observed at 24 h as net Hg(0) accumulation arising from the evolving balance among these coupled processes, together with time-dependent changes in Hg ligation and DOM mineral interactions.

6. Line 195–197: "a characteristic energy shift toward lower energy indicating more reduced Hg species". If this is correct, it would imply the presence of Hg(0) in the EXAFS samples. Therefore, the authors should provide detailed information on sample preparation and preservation for XAS measurements. For example: Were the samples frozen immediately after collection? Were the XAS measurements conducted at cryogenic temperatures (e.g., liquid nitrogen or liquid helium)? Furthermore, the presence of Hg(0) should be confirmed using XANES analysis or EXAFS fitting.

Response: Thank you for the constructive comment. The Hg L_{III} edge XAS were measured directly as freshly prepared suspensions rather than as frozen or dried samples. Sample preparation followed the same experimental sequence used in the kinetic experiments, and the timing of each preparation step was scheduled in advance according to the beamtime so that the target reaction stage was reached at the time of measurement. All samples were kept under strict dark conditions before analysis to

minimize light-induced Hg transformation. We also clarify that, to improve the Hg signal intensity, a higher Hg concentration was used for the XAS measurements. However, the Hg signal quality remained insufficient for EXAFS fitting. For this reason, we used the Hg-XAS data conservatively and only as qualitative support for differences among treatments. We have now added these details in the Methods in Lines 583–586.

7. Line 211–212: "the mineral-Hg-DOM ternary complex formation controls the fate of Hg in natural environments". This statement seems inconsistent with the earlier claim that thiol groups in DOM are not saturated and that Hg forms $Hg(RS)_2$ complexes. Because Hg has extremely strong affinity for thiol groups, $Hg(RS)_2$ complexes should dominate over Hg–mineral interactions. The stability constants differ by many orders of magnitude. Therefore, the estimation of thiol concentrations in DOM should be carefully evaluated (and the estimation of thiol concentration should be detailed described).

Response: Thank you for raising this point. The original wording may have suggested that mineral interactions generally override the strong binding of Hg to thiol groups in DOM. This was not our intention. To avoid this misunderstanding, we have revised the sentence to "These results indicate that the association of Hg-DOM complexes with mineral surfaces forms mineral-Hg(II)-DOM ternary complexes that are critical for Hg speciation and its dark reduction", following Major comment 1. This revised wording clarifies that our interpretation does not conflict with the strong affinity of Hg to thiol groups. Our point is that after initial Hg-DOM complexation, further association of these Hg-DOM complexes with mineral surfaces can introduce additional effects on Hg partitioning and reduction behavior in the ternary system. The estimation of thiol concentrations in DOM has also been revised in response to Specific Comment 1. The corresponding description has been clarified in the Methods section (Lines 519–527), where we now provide the basis of the calculation and the resulting Hg/RSH molar ratios for the LR and HR systems.

8. Line 230–233: The statement regarding $Hg(OR)_2$ complexes hindering ligand exchange is likely incorrect. $Hg(RS)_2$ complexes are the most stable species and would be expected to inhibit ligand

exchange. In contrast, $Hg(OR)_2$ complexes compete with mineral surface binding. If thiol groups are not saturated, $Hg(DOM\text{-}RS)_2$ complexes should dominate. Only when thiol groups become saturated would excess Hg bind to oxygen-containing groups in DOM or mineral surfaces. A thermodynamic speciation model should be used to estimate the relative abundance of $Hg(DOM\text{-}RS)_2$, Hg–DOM–mineral complexes, and Hg–mineral complexes.

Response: Thank you for the helpful comment. The interpretation we intended here is broadly consistent with your point, but our original wording was not sufficiently precise. We have revised it to make the interpretation more cautious in Lines 233–237 to clarify that, at relatively high Hg concentration, when thiol groups on DOM become saturated, Hg may be competitively bound by oxygen-containing groups in DOM and by reactive sites on the mineral surface. Under these conditions, competition for Hg may weaken the ligand exchange processes involved in DOM adsorption to goethite and may also alter DOM charge neutralization and conformation, resulting in lower DOM adsorption in the HR system than in the LR system.

A thermodynamic speciation model could help quantify Hg partitioning among DOM-bound and mineral-associated species. For the present system, however, this would require better constrained binding site distributions and stability constants for Hg binding to DOM, mineral surfaces, and ternary interfacial species. These parameters remain poorly constrained for this type of ternary system. Including a formal speciation model under these conditions would introduce substantial uncertainty beyond what the current data can support. In this study, we focus on experimental evidence for how mineral-Hg(II)-DOM interactions affect Hg partitioning and dark reduction. Quantitative speciation modeling will be an important direction for future work.

9. Line 375–379: The calculation results are interesting. However, more experimental evidence is required to support the conclusion that mineral-Hg-DOM complexes dominate over $Hg(Cys/SA)_2$-like complexes (in lab experiments and natural environments).

Response: Thank you for the helpful comment. The original wording in this part was not sufficiently precise and may have led to an overinterpretation of the calculation results. Based on the calculated binding energies, the more appropriate conclusion is

that monodentate Hg-ligand complexes such as $[Cys\text{-}Hg]^{+}$ and $[SA\text{-}Hg]^{+}$ are more likely to associate with goethite surface groups than with additional dissolved ligands. We did not intend to imply that mineral-Hg(II)-DOM complexes dominate over $Hg(Cys/SA)_2$-like complexes in laboratory systems or natural environments. This part has been revised accordingly in Lines 379–381.

10. Line 382–384: The statement that bidentate structures such as $Hg(OR)_2$, RSHgOR, and $Hg(SR)_2$ are thermodynamically less stable requires clarification. In addition, the experimental results show enhanced Hg(0) production in the presence of goethite. If $Hg(OR)_2$, RSHgOR, and $Hg(SR)_2$ are less stable than Hg–goethite–DOM ternary complex, this interpretation may appear contradictory.

Response: Thank you for the constructive comment. Based on the calculated binding energies, what we intended to convey is that these bidentate coordination structures may partially reorganize upon mineral association, allowing Hg to interact more closely with reactive goethite surface sites and form mineral-Hg(II)-DOM complexes. From this perspective, the enhanced net Hg(0) production is not contradictory, because it reflects the combined effects of mineral surfaces and DOM rather than a simple comparison of isolated Hg complexes. Specifically, our subsequent calculations indicate that the reduction energy barrier of Hg is lowered in the ternary mineral-Hg(II)-DOM complex, while DOM fractionation retains more electron-donating components in the aqueous phase. Together, these coupled effects promote Hg(II) reduction at both the mineral-water interface and in solution. We have revised this part in Lines 386–390 accordingly to make the interpretation more precise and rigorous.

11. Line 387–391: The discussion should include a more detailed consideration of Hg^0 reoxidation, since the observed Hg^0 production represents a net result of reduction and oxidation processes.

Response: Thanks. We agree that the observed Hg(0) production represents the net balance between reduction and reoxidation. This has been clarified in the revised manuscript in Lines 115–116. As discussed in response to Specific comment 10, the increase in net Hg(0) production in the goethite-Hg(II)-DOM system is interpreted to be mainly driven by enhanced Hg(II) reduction resulting from the combined effects of

mineral surfaces and DOM. Although Hg(0) reoxidation may occur, it is not expected to offset the overall increase in Hg(0) formation under our experimental conditions.

12. Line 482–483: Batch experiments were conducted to examine the sorption of DOM (0.83–8.75 mM) and Hg(II) (Hg(NO3)2, 0.32–13.34 µM) on goethite (2 g/L)." This description is unclear. Does it refer to:

1. DOM adsorption on goethite

2. Hg adsorption on goethite

3. adsorption of the DOM–Hg mixture on goethite?

The concentrations listed here also differ from those described in the following paragraph.

Response: Thanks. This sentence refers specifically to the concentration ranges used in the isotherm adsorption experiments. The same concentration framework was applied across all isotherm adsorption systems in this study, including Hg adsorption on goethite, DOM adsorption on goethite, and the goethite-Hg(II)-DOM ternary system. We have revised the text in Lines 506–509 to make this clearer.

The different concentrations given in the following paragraph correspond to a different purpose of the experiment:

The kinetic adsorption experiments: The lower concentrations were used in the kinetic experiments to better match environmentally relevant levels. Meanwhile, the kinetic experiments required Hg(0) to be purged at selected time points, because this approach offered the best efficiency and the lowest measurement uncertainty in our setup. The detection range of the Hg analyzer (Brooks Rand Model III) did not allow us to use Hg concentrations as high as those used in the isotherm adsorption experiments.

XAS measurement: In our previous XAS tests, the Fe signal from the mineral phase was extremely strong, while the Hg signal remained difficult to detect even at relatively high Hg concentration, even the highest-Hg isotherm sample HR-6 at 13.34 µM Hg. For this reason, we used a higher Hg concentration and lower goethite concentration during Hg-XAS analysis. Nonetheless, the signal of Hg was still insufficient to support the fitting of the extended edges. Thus, we used the Hg-XAS results very cautiously in

the present study, mainly to compare the effect of addition sequence on Hg redox behavior rather than to make broader quantitative interpretations.

13. Line 489–493: More information should be provided regarding the concentration of thiol groups in the PPHA. Table S1 only reports total sulfur concentrations, which do not necessarily represent thiol groups. Without quantitative information on thiol concentration, it is premature to conclude that Hg(II) is primarily complexed with thiol groups.

Response: Thanks. This point has also been addressed in our response to Specific Comment 1, where we clarified the basis used to estimate thiol concentrations in PPHA from the total sulfur content and provided the corresponding Hg/thiol calculations. The relevant description has been revised accordingly in Lines 519–527.

14. Adsorption experiments: more experimental details should be provided, particularly for systems containing DOM. The adsorption behavior of DOM is strongly influenced by pH, ionic strength, and other solution parameters.

Response: Thanks for the suggestion. The pH condition was already given in the Methods section in Lines 513–514. To make the experimental conditions clearer, we have clarified that ultrapure water was used as the background solution. We also added that chloride was avoided during the experiments to minimize possible interference from Hg-Cl complexation. These details have been revised in Lines 514–516.

15. Line 494: Why were Hg(II) and DOM equilibrated for only 8 h before adding goethite? Hg–DOM complexation is often kinetically controlled, and many studies use 24 h equilibration.

The procedure described in this paragraph is not entirely clear. For example, was the sequence: DOM + Hg(II) → 8 h equilibration → addition of goethite? Was purging performed before or after goethite addition?

Response: Thank you for the helpful comment. The experimental sequence has been revised to make the procedure clearer. Specifically, Hg(II) and DOM were first pre-equilibrated for 8 h, followed by goethite addition. The ternary suspensions were then

reacted for 24 h before Hg(0) purging and measurement. This has now been clarified in revised Fig. 1a and in Lines 529–530. The 8 h pre-equilibration used here was intended to allow sufficient association between Hg and DOM before goethite addition. Reported equilibration times in Hg-DOM studies vary, with pre-equilibration periods ranging from 3 h to overnight, depending on the experimental condition. 8 h is within a suitable equilibration range in this study. In the kinetic experiments, equilibration of Hg-DOM association was faster at lower Hg concentrations, typically within about 1 to 2 hours in control experiment iv and related experiments (results not shown here). The adsorption time of the ternary system is 24 h, which has been illustrated in our method.

16. Line 506: low Hg(II) concentrations instead of low Hg(0) concentrations?

Response: Thank you for pointing it out. We aimed to report the Hg(0) generated in the kinetic experiments, rather than to imply that low levels of Hg(0) were initially present. We have revised the sentence to make it much clearer that Hg(0) generated in the kinetic experiments was purged from the reaction system and measured directly using the Hg analyzer in Lines 544–547.

17. Line 546–547: The ratio used in the model system (1 mM DOM with 5 mM Hg) is extremely different from the experimental conditions.

Response: Thanks. This issue has also been addressed in our response to Specific Comment 12. The 1 mM DOM and 5 mM Hg were used only in the model system for XAS-related analysis. These conditions were intentionally adjusted to improve Hg detectability because the Fe signal from the mineral phase was extremely strong, and Hg remained difficult to detect even at the highest Hg concentration used in the isotherm adsorption experiments. We therefore used this model system only for cautious qualitative comparison rather than for direct quantitative comparison with the experimental systems.

18. Line 555–556: SA and cysteine cannot represent natural DOM. Although these molecules contain carboxyl and thiol functional groups, natural DOM has complex three-dimensional

structures and heterogeneous functional group distributions that cannot be mimicked by simple molecules. Is it possible to use big molecules, for instance, proteins?

Response: Thank you for the thoughtful suggestion. This concern has been noted in the manuscript in Lines 367–375. As we stated there, Cys and SA were not used to represent natural DOM, but as simplified model ligands to examine Hg binding and reduction behavior under well-defined conditions. We have clarified that "these small molecules cannot represent the structural complexity of natural DOM, where steric constraints and cooperative interactions among multiple functional groups may influence binding strengths and reaction pathways", so the calculated binding energies should be interpreted only at the functional group level. Larger molecules such as proteins would provide a more structurally complex model, but they also introduce much greater computational difficulty because of their size, conformational flexibility, and multiple possible binding configurations. For this reason, we chose Cys and SA as tractable model compounds for mechanistic comparison, while keeping the interpretation appropriately cautious.
